# Synthesis of highly substituted fluorenones via metal-free TBHP-promoted oxidative cyclization of 2-(aminomethyl)biphenyls. Application to the total synthesis of nobilone

**DOI:** 10.3762/bjoc.17.181

**Published:** 2021-11-02

**Authors:** Ilya A P Jourjine, Lukas Zeisel, Jürgen Krauß, Franz Bracher

**Affiliations:** 1 Department of Pharmacy - Center for Drug Research, Ludwig-Maximilians University of Munich, Butenandtstraße 5–13, 81377 Munich, Germany

**Keywords:** cross-dehydrogenative coupling, cyclization, fluorenones, nobilone, total synthesis

## Abstract

Highly substituted fluorenones are readily prepared in mostly fair to good yields via metal- and additive-free TBHP-promoted cross-dehydrogenative coupling (CDC) of readily accessible *N*-methyl-2-(aminomethyl)biphenyls and 2-(aminomethyl)biphenyls. This methodology is compatible with numerous functional groups (methoxy, cyano, nitro, chloro, and SEM and TBS-protective groups for phenols) and was further utilized in the first total synthesis of the natural product nobilone.

## Introduction

**Scheme 1:**
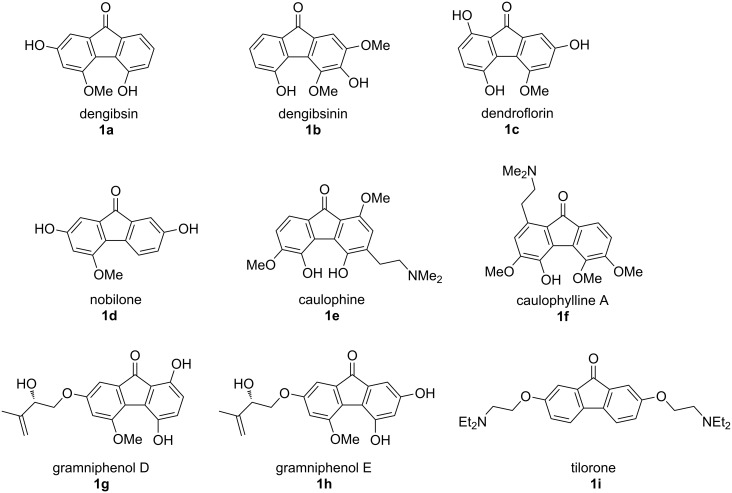
Selected fluorenone-type natural products.

Fluorenones are an important class of aromatic natural products, and since the identification of the first representatives, dengibsin (**1a**) and dengibsinin (**1b**) in 1985 from the orchid *Dendrobium gibsonii* [1], numerous further natural fluorenones, typically bearing hydroxy and methoxy substituents, but also aminoalkyl side chains, as in caulophine (**1e**) [2] and caulophylline A (**1f**) [3], were identified (Scheme 1). However, a couple of structure assignments had to be revised, mostly based on results from total syntheses [4–5].

Numerous biological activities have been reported for natural fluorenones, e.g., antioxidative properties of dendroflorin (**1c**) and nobilone (**1d**) [6], antiischemic activity of caulophine (**1e**) [7], and anti-HIV activity of gramniphenol D (**1g**) and related gramniphenol E (**1h**) [8]. For synthetic fluorenones antitumoral [9], antiviral [10], and trypanocidal [11] activities have been reported, and tilorone (**1i**), an antiviral fluorenone launched about 50 years ago, is presently discussed as a therapeutic option for fighting Ebola and SARS-CoV-2 viruses [12]. Modifications of the tilorone scaffold resulted in compounds having cytokine-inducing [13], antitumor [14], and telomerase-inhibitory effects [15]. Further synthetic fluorenones and polyfluoren(on)es show attractive electronic and optical properties [16–21], utilized in LEDs, semiconducting polymers, and solar cells.

**Scheme 2:**
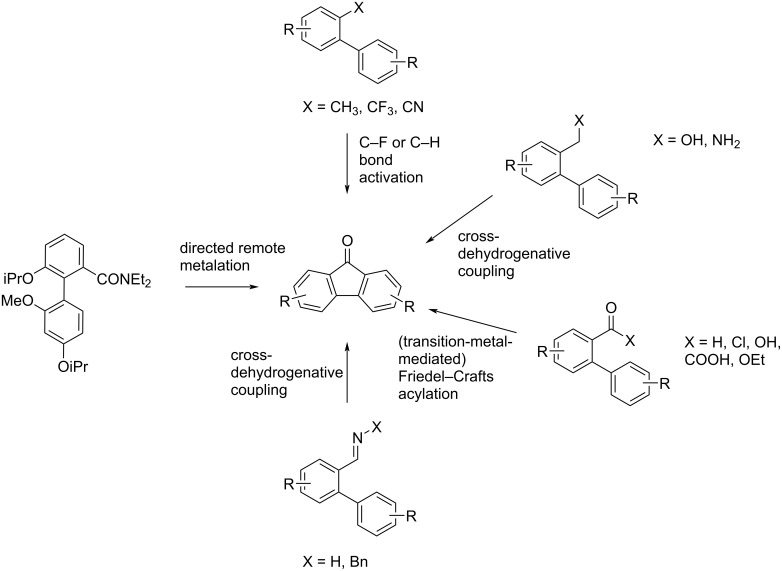
Overview of published cyclization methodologies for the synthesis of fluorenones starting from functionalized biaryls.

Consequently, synthetic approaches to fluorenones have attracted large interest and numerous methodologically diverse approaches have been published in the past decades. Among these are radical cyclizations [22], Pschorr reactions [23], and diverse cycloaddition protocols [24–25]. Especially transition-metal-catalyzed cross-coupling reactions starting from benzophenones, benzoic acids, dihalogenated benzene building blocks and others have emerged as new approaches in recent years [26–28]. Various approaches starting from functionalized biaryls have hereby attracted considerable interest, since the precursors are readily available by established cross-coupling reactions. Beyond transition-metal-catalyzed reactions, acid-mediated cyclizations of biphenylcarboxylic acids and activated derivatives (intramolecular Friedel–Crafts acylation) [29–31] found wide application here. In a different approach, a total synthesis of dengibsin (**1a**) was accomplished by Wang and Snieckus in 15 steps by means of a directed remote metalation [32], using a benzamide residue as the directing group (Scheme 2) [33].

In contrast to transition-metal-mediated approaches [27], metal-free oxidative methods are attractive not only from an ecological point of view, but also due to the typically low cost of the applied oxidants. Biarylcarboxaldehydes were cyclized to fluorenones using K_2_S_2_O_8_ [34], CBr_4_ [22], or peroxides like *tert*-butyl hydroperoxide (TBHP) [35]. Other oxidative cyclizations have been developed starting from biarylglyoxylic acids (using Na_2_S_2_O_8_) [36], and even 2-methylbiphenyls and 2-(hydroxymethyl)biphenyls can be converted into fluorenones by means of TBHP oxidation [37]. In contrast, only very few reports deal with the oxidative cyclization of nitrogen-containing biaryl intermediates. Ravi Kumar and Satyanarayana mentioned two successful control reactions using 2-phenylbenzylamine and 2-iminomethylbiphenyl with 5 mol % Pd(OAc)_2_ and 2 equivalents Ag_2_O in acetic acid at 140 °C, giving fluorenone (**3**) in 62 and 76% yields [38]. Tadigoppula reported a PhI(OAc)_2_–BF_3_·OEt_2_-mediated cyclization of aldimines [39].

This prompted us to perform a systematic investigation of the potential of oxidative cyclizations of aminomethylated biaryls **2** and related compounds to fluorenones. Benzylamines and derivatives thereof have been described in literature to be susceptible to oxidation by diverse reagents (tritylium ion [40], silver [38] and cerium salts [41], peroxides [42–44] and persulfates [45], nitroxyls [46], hypervalent iodine compounds [39,47], or tetrahalomethanes [48]) to give imines, iminium salts, aldehydes and other, in some cases dimeric products [49]. Here, oxidation of the benzylic amino moiety should lead either to iminium ions (or *N*-acyl iminium ions) **4a** as strong electrophiles or to stabilized radicals **4b** which could undergo cyclization to give the fluorenone backbone. Expected 9-aminofluorene intermediates **5** were envisaged to undergo subsequent oxidation by the same oxidant to hopefully provide the fluorenones **3** in a domino reaction.

## Results and Discussion

**Scheme 3:**
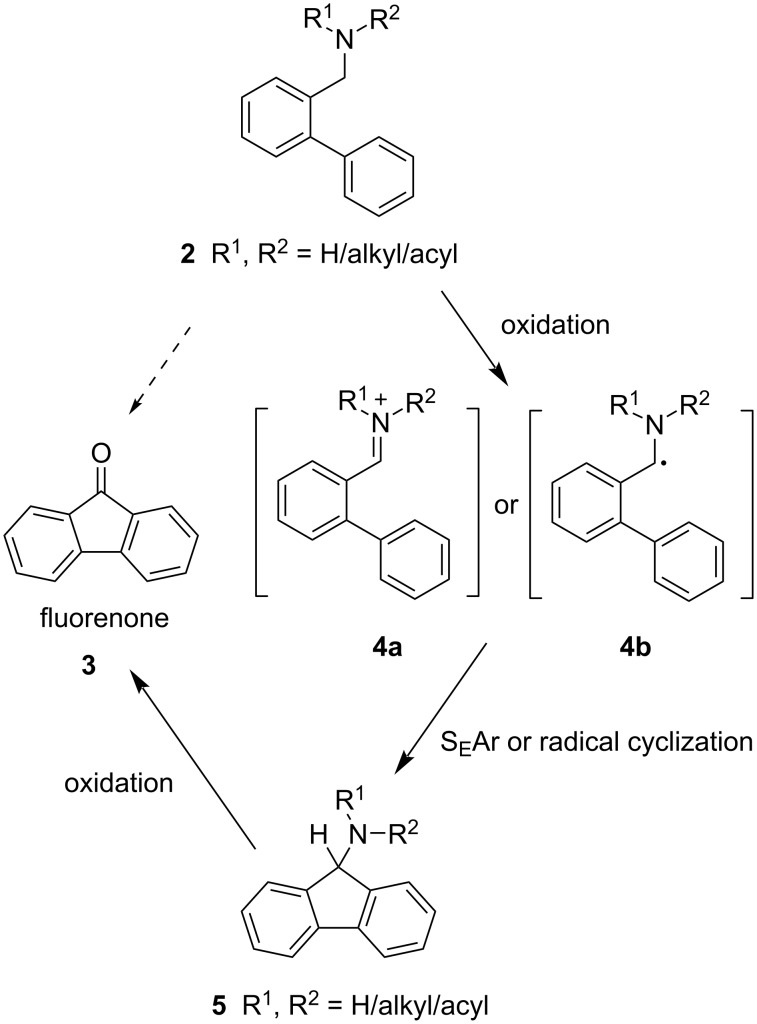
Preliminary considerations for the oxidative cyclization of 2-(aminomethyl)biphenyls to fluorenones.

After comprehensive literature search for successful oxidations of benzylic C–N bonds we tested a variety of oxidizing agents, oxidizing systems, and radical initiators on a set of model molecules **2** (see Table 1) in a preliminary screening for suitable oxidants for the intramolecular ring-closure reaction. The set of model molecules **2** bears different benzylic *N*-containing functional groups, including secondary and tertiary amines, amides/lactams/carbamates, and nitrile. The test reactions were monitored by thin-layer chromatography (TLC) and, where deemed necessary, results were further verified by GC–MS. The reagents employed encompassed tritylium tetrafluoroborate [50], H_2_O_2_/HBr [42], ceric ammonium nitrate (CAN) [41], 2,2,6,6-tetramethylpiperidinyloxyl (TEMPO)/CuCl [51], K_2_S_2_O_8_ [36], dimethyl sulfoxide (DMSO)/O_2_ [52], PhI(OAc)_2_/benzoyl peroxide (BPO) [47], Dess-Martin periodinane, *N*-bromosuccinimide (NBS), *N*-hydroxyphthalimide (NHPI)/Co(OAc)_2_/O_2_ [53], H_2_O_2_/tetrabutylammonium iodide (TBAI) [43], CBr_4_ [22], and *tert*-butyl hydroperoxide [37] (TBHP). Formation of postulated intermediate aminofluorenes of type **5** could not be observed for any of the reactions performed during the initial screening process, and cyclization to the target compound fluorenone (**3**) was confirmed for only two of the substrate/reagent combinations employed. The reaction of the secondary *N*-methylamine **2b** with CBr_4_ [22] gave fluorenone (**3**) in 6% yield (determined by GC–MS), while tertiary amines, amides, and the γ-lactam **2e** did not yield any. Formation of fluorenone (**3**) was also confirmed after applying a modified version of one of the TBHP-cyclization protocols reported by Laha et al. [37] (we used the cheaper aqueous TBHP solution (70%) instead of TBHP in *n*-decane) and gave the most promising results: Reacting *N*-methylamine **2b** with 4 equivalents of aqueous TBHP in 1,2-dichloroethane (1,2-DCE) at 100 °C for 18 h afforded fluorenone (**3**) in 30% yield (determined by GC–MS). TLC analysis further revealed that fluorenone formation does not occur when treating tertiary amides **2h** and **2i**, γ-lactam derivative **2e**, and carbamate **2j** with this reagent (Table 1, entries 5, 8, 9, and 10). The reaction does, however, also work with tertiary amines **2c**, **2d**, and **2f**, albeit in drastically lower yield (Table 1, entries 3, 4, and 6). Initially, our focus was on testing tertiary amines, as we speculated that the electrophilic iminium species **4a** (R^1^, R^2^ = alkyl; Scheme 3) would form more easily with stabilizing electron-donating alkyl substituents through inductive effects, and on amides, which should be more prone to engage in the cyclization owing to the high reactivity of expected *N*-acyliminium ions **4a (**R^1^ = alkyl, R^2^ = acyl) in S_E_Ar reactions. In the second screening round, we determined the isolated yields of the promising reactions identified in the initial screening. The results of both screening rounds are summarized in Table 1. To our delight, fluorenone (**3**) could be isolated with a fair yield of 60% starting from *N*-methylamine **2b**, far exceeding the projected yield determined by GC–MS before. The TBHP-mediated cyclization of primary amine **2a**, which was not part of the initial screening round, afforded fluorenone (**3**) with a similar yield of 62% (Table 1, entry 1). In agreement with our initial screening, tertiary amide **2h** did not yield any fluorenone (**3**), however secondary amide **2g** afforded the product in a poor yield of 20% (Table 1, entries 7 and 8). The primary alcohol **2k** and aldehyde **2l**, both bearing oxygen-containing functional groups instead of nitrogen adjacent to the reactive center, gave 26% and 25% of the target compound **3** under these conditions, respectively (Table 1, entries 11 and 12). The TBHP-mediated cyclization of primary alcohols like **2k** has been successfully utilized in the synthesis of fluorenones and azafluorenones [37], however, the authors used TBHP in *n*-decane. We repeated the reaction for primary amine **2a**, primary alcohol **2k**, as well as aldehyde **2l** with TBHP in *n*-decane and obtained fluorenone (**3**) in 22%, 60% and 26% yield, respectively (Table 1, entries 1, 11 and 12, all under conditions b). This leads us to the conclusion that, different from aldehydes, the success of the cyclization for amines is highly dependent on the solvent in which TBHP is dissolved, indicating that water may have a beneficial effect here. Notably, the reaction of nitrile **2m** did not yield any target compound **3** (Table 1, entry 13). Substituted 2-phenylbenzonitriles have been reported to yield fluorenones in good yields under Pd-Ag-catalysis in previous research efforts [54].

**Table 1:** Reactivity of different functional groups towards TBHP-mediated cyclization to give fluorenone (**3**).

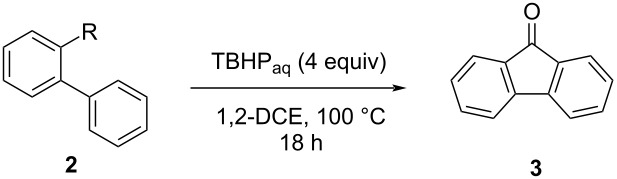								

entry	substrate R =	yield of **3** (%)	entry	substrate R =	yield of **3** (%)	entry	substrate R =	yield of **3** (%)

1	-CH_2_-NH_2_ (**2a**)	62 22^b^	6	-CH_2_-morpholin-4-yl (**2f**)	^a^	11	-CH_2_-OH (**2k**)	26 60^b^

2	-CH_2_-NHCH_3_ (**2b**)	60	7	-CH_2_-NH-COCH_3_ (**2g**)	^a^ 20	12	-CHO (**2l**)	25 26^b^

3	-CH_2_-N(CH_3_)_2_ (**2c**)	^a^	8	-CH_2_-NCH_3_-COCH_3_ (**2h**)	0	13	-CN (**2m**)	^a^

4	-CH_2_-N-cC_4_H_8_ (**2d)**	^a^ 13	9	-CH_2_-NCH_3_-COCF_3_ (**2i**)	^a^			
	
5	-CH_2_-pyrrolidon-1-yl (**2e**)	^a^	10	-CH_2_-NCH_3_-Boc (**2j**)	^a^			

^a^Trace amounts or no product formation determined by TLC monitoring and GC–MS analysis as part of the initial screening; ^b^isolated yields of the reactions carried out with TBHP solution in *n*-decane (80%).

At this point we concluded that out of all nitrogen-containing investigated precursors only primary and secondary benzylamines (see **2a** and **2b**), giving fluorenone (**3**) in yields of 62% and 60%, respectively, are suitable functional groups for the TBHP-mediated oxidative cyclization to give fluorenones. These yields are comparable to those previously published for fluorenone syntheses starting from other functionalized biphenyls with a key oxidative cyclization step. Starting from biphenyl-2-carbaldehyde (**2l**), Shi and Glorius [34] reported a fluorenone yield of 68%, utilizing potassium persulfate/tetraethylammonium bromide, the Singh group [35] reported a yield of 62% employing TBHP_aq_ and the Studer group [55] achieved, with a ring-methylated analogue, up to 72% with TBHP in decane and FeCp_2_ as initiator. Laha et al. [37] cyclized biphenyl-2-methanol (**2k**) in 70% yield using TBHP in decane/tetrabutylammonium iodide.

Primary amine **2a** was chosen as the model compound to further optimize the yield of the cyclization (Table 2). Different molarities of TBHP (Table 2, entries 2–5), reaction times (Table 2, entry 6), solvents (Table 2, entries 7–9) and additives, inspired by published protocols for benzylic oxidations and oxidative cyclizations [37–38,56] (Table 2, entries 10–13), were employed. The yield of aldehyde **2l** as the most prominent side product and possible intermediate involved in the oxidative cyclization was also determined. Unfortunately, the initial conditions could not be improved upon, as all the changes implemented had an adverse effect on the yield of fluorenone (**3**). Addition of TBAI (Table 2, entry 10) in particular looked promising, as TBAI/TBHP-mediated radical cyclizations and cross-dehydrogenative coupling (CDC) reactions are not only well established [57], but addition of TBAI has been shown to increase the yield of fluorenone (**3**) in a TBHP-mediated cyclization of alcohol **2k** under otherwise very similar reaction conditions in previously published research [37]. The reaction was also performed under alkaline conditions (addition of KOH), however, the yield of fluorenone (20%) decreased significantly in favor of more aldehyde (33%) being generated (Table 2, entry 11). Similar results were obtained when adding iodine to promote benzylic oxidation [56] (Table 2, entry 12). Finally, Pd(OAc)_2_ was added in hopes of improving the mediation of C–C bond formation [38] (Table 2, entry 13). Interestingly, here the yield of fluorenone (59%) is only slightly lower compared to the standard reaction while the yield of the aldehyde has increased notably (23%). Unable to improve the initial reactions conditions within the framework of this optimization study, we continued our studies with the standard conditions described in entry 1 (Table 2).

**Table 2:** Optimization study for the oxidative cyclization of primary amine **2a** with aqueous TBHP.

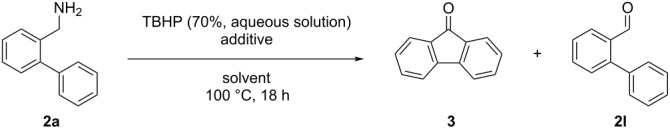					

entry	equivalents of TBHP	equivalents of additive	solvent	yield of **3** (%)	yield of **2l** (%)

1	4		1,2-DCE	62	3
2	1		1,2-DCE	11	23
3	3		1,2-DCE	40	9
4	5		1,2-DCE	59	trace
5	10		1,2-DCE	42	trace
6^a^	4		1,2-DCE	61	22
7	4		ACN	trace	15
8	4		dioxane^b^	trace	36
9	4		DMSO	0	34
10	4	TBAI (0.05)	1,2-DCE	33	16
11	4	KOH (1)	1,2-DCE	20	33
12	4	I_2_ (0.05)	1,2-DCE	17	21
13	4	Pd(OAc)_2_ (0.05)	1,2-DCE	59	23

^a^Reaction time = 30 h; ^b^stabilized with 2 to 5 ppm BHT.

Next, we sought to further characterize the substrate scope by reacting various ring-substituted 2-(aminomethyl)biphenyls and *N*-methyl-2-(aminomethyl)biphenyls to the corresponding fluorenones. Our focus was strongly (but not exclusively) on oxygen-containing residues, since these occur frequently and at various positions in fluorenones from nature.

**Scheme 4:**
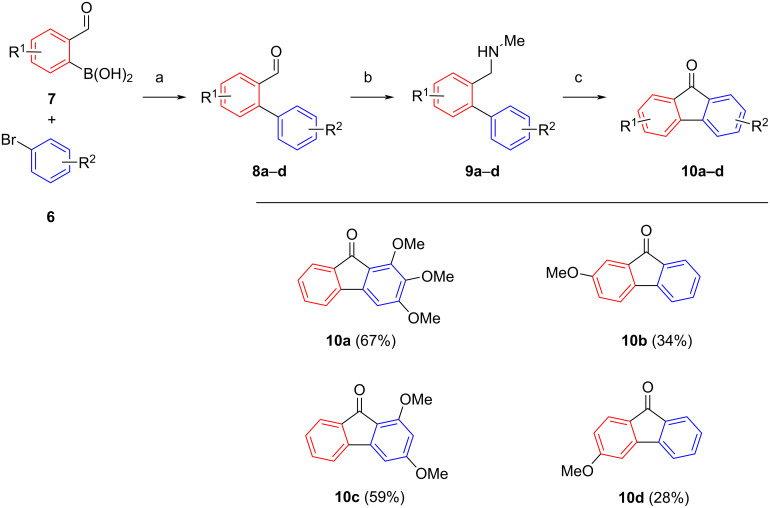
Substrate scope and yields for oxidative cyclizations of *N*-methyl-2-(aminomethyl)biphenyls **9a–d** bearing methoxy residues. Conditions: a) Pd(PPh_3_)_4_ (5 mol %), Na_2_CO_3_, DMF/H_2_O, 18 h, 100 °C, 76–99%; b) MeNH_2_, NaBH_4_, DCM, rt, 62–78%; c) TBHP_aq_, 1,2-DCE, 100 °C, 18 h (yields in parentheses).

**Scheme 5:**
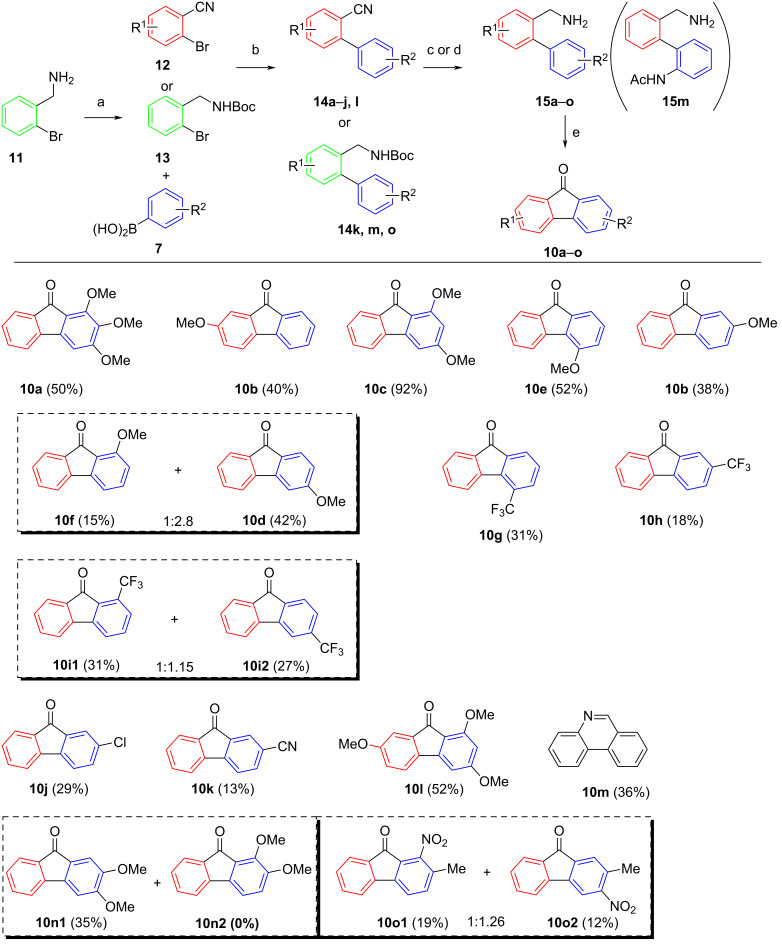
Substrate scope for the oxidative cyclization of 2-(aminomethyl)biphenyls. Conditions: a) Boc_2_O, NEt_3_, DCM, rt, 18 h, 89%; b) Pd(PPh_3_)_4_ (5 mol %), Na_2_CO_3_, DMF/H_2_O, 18 h, 100 °C, 83–99%; c) LAH, AlCl_3_, THF, 18 h, 43–78%; d) TFA, DCM, rt, 6 h, 76–98%; e) TBHP_aq_, 1,2-DCE, 100 °C, 18 h (yields in parentheses).

**Scheme 6:**
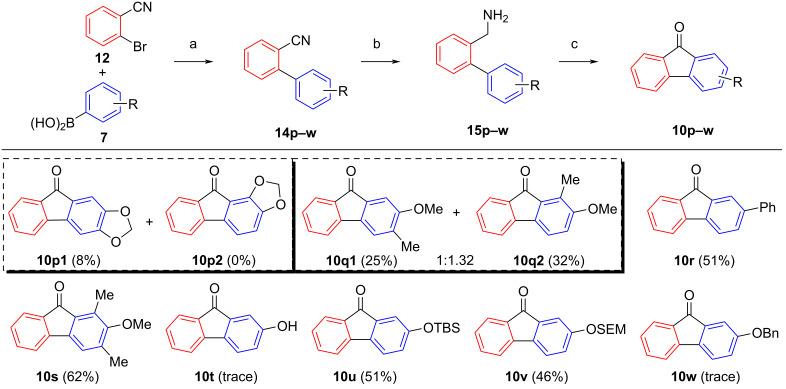
Substrate scope for the oxidative cyclization of 2-(aminomethyl)biphenyls with main focus on protected phenols. Conditions: a) Pd(PPh_3_)_4_ (5 mol %), Na_2_CO_3_, DMF/H_2_O, 18 h, 100 °C, 76–99%; b) LAH, AlCl_3_, THF, 18 h, rt; c) TBHP_aq_, 1,2-DCE, 100 °C, 18 h (yields in parentheses).

**Scheme 7:**
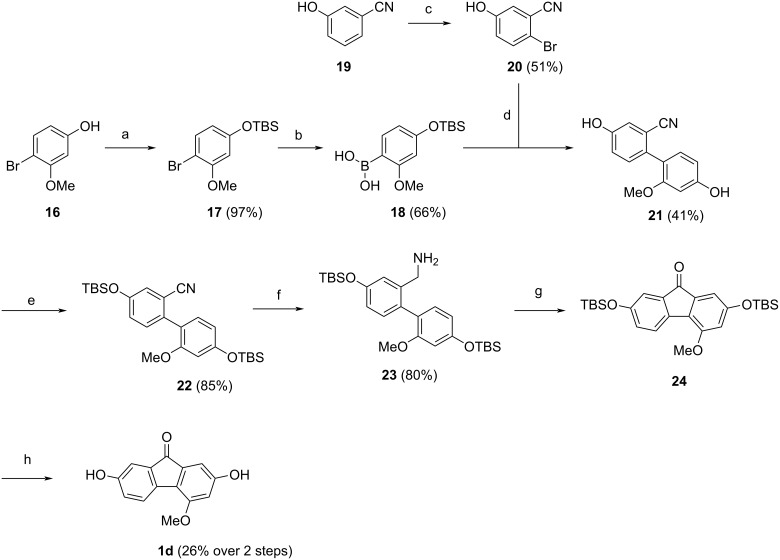
Total synthesis of nobilone (**1d**). Conditions: a) TBS-Cl, imidazole, DMF, 50 °C, 18 h; b) *n*-BuLi, B(OiPr)_3_, THF, −78 °C to rt, 16 h; c) BF_3_·OEt_2_, NBS, −20 °C to rt, 24 h; d) Pd(PPh_3_)_4_ (5 mol %), Na_2_CO_3_, DMF/H_2_O, 100 °C, 12 h; e) TBS-Cl, imidazole, DMF, 50 °C, 18 h; f) LAH, AlCl_3_, THF, rt, 12 h; g) TBHP_aq_, DCE, 100 °C, 18 h; h) pyridine, HF·pyridine, EtOAc, rt, 14 h.

The model cyclization precursors were prepared in two step syntheses by Suzuki coupling [58] of commercially available *ortho*-substituted areneboronic acids **7** and bromobenzenes **6**, **12**, and **13** followed by either reductive amination [59] in the case of biaryl aldehyde intermediates **8**, reduction [60] in the case of nitriles **14**, or Boc deprotection [61] for **14k**, **m**, **o** according to standard protocols (Schemes 4–7; for details, see Supporting Information File 1). Although it may seem counterintuitive to first convert aldehydes **8** and nitriles **14** into the corresponding amines **9** and **15** when fluorenones can be obtained directly from aldehydes [35,37] and, to a lesser extent, nitriles [54], it was our goal to characterize amine cyclization furnishing fluorenones specifically. Amine cyclization of this nature is only punctiformly described in the existing literature [38] and further investigations may thus give way to new synthetic possibilities in natural products synthesis in cases access to or cyclization of benzylamine moieties appears practical. Moreover, aldehyde precursors may not only be less readily available than nitrile precursors but also less desirable as reaction intermediates, owing to the difference in their respective reactivity.

The results for the route going through *N*-methyl-2-(aminomethyl)biphenyls **9** are summarized in Scheme 4. We found that substrates bearing electron-donating groups at the aminomethyl carrying arene (**9b** and **9d**) afforded significantly lower yields (28 and 34%) than substrates with electron-donating substituents at the other arene (**9a** and **9c**; 59–67%), which were in the same range as the model reaction with unsubstituted amine **2b**.

Next, reactions with primary 2-(aminomethyl)biphenyls were investigated (Scheme 5 and Scheme 6). First, the three primary amines **15a–c** with the same substitution patterns as reported for the secondary amines **9a–c** (Scheme 4) were reacted under the standard conditions. While the yield for fluorenone **10b** starting from primary amine **15b1** (40%) is slightly higher compared to its counterpart starting from secondary amine **9b** (34%), the trend of electron-donating groups at the aminomethyl carrying arene adversely affecting the yield was also observed for primary amines. The negligible difference in yields for fluorenone **10b** starting from amine **15b1** (34%) and **15b2** (38%) respectively, lead us to believe that the substitution pattern is what determines the efficiency of the reaction rather than which of both rings the substituents are connected to. Surprisingly, fluorenone **10c** was isolated in an excellent yield (92%). Based on these datapoints, no clear conclusion can be drawn as to whether primary or secondary benzylic amines are more suited for this cyclization. Nevertheless, we decided to further characterize the TBHP-mediated cyclization using primary amines, as these typically are more readily accessible. Comparing the yields for methoxyfluorenones **10e** (52%) and **10b** (obtained from amine **15b2**; 38%), it seems that congeners with electron-donating substituents in the *para*-position at the (supposedly) radical accepting arene moiety are unfavorable in comparison to the *ortho*-substituted isomers. As expected, amines with substituents in *meta* positions gave mixtures of regioisomers. Methoxyfluorenone **10f** was afforded in 15% yield and methoxyfluorenone **10d** starting from amine **15f** in 42% yield. Notably, only **10d** was obtained upon cyclization of amine **9d** (Scheme 4). Interestingly, the combined yield of the two regioisomers (57%), stemming from one educt exceeds the yields of both **10e** and **10b** starting from amine **15b2**. The same trend concerning relative yields between regioisomers can be observed for fluorenones bearing the electron-withdrawing trifluoromethyl group in the same positions (see **10g**, **10h**, **10i1**, and **10i2**). This is rather surprising, as electronic effects exerted by the radical accepting moiety in similar TBHP-promoted reactions leading to xanthones have been observed to have little impact on cyclizations of this nature [55]. The series of fluorenones **10h** (18%), **10j** (29%), and **10k** (13%) with electron-withdrawing groups of varying electronegativity in the *para* position indicates an inversely proportional relation between the strength of the −*I* effect exerted by substituents and the efficiency of the cyclization. Reaction of amine **15m** (see Scheme 5) did not give the corresponding fluorenone, instead phenanthridine **10m** was isolated in 36% yield. Most likely, radical accepting arene moieties with *N*-acylamino residues in the *ortho* position are incompatible with the reaction conditions.

To our surprise, only one regioisomer was found for some of the cyclizations performed with *meta*-substituted amines (**10n1** and **10p1**). Comparing the isomer pairs **10o1/2** (31% combined yield) and **10q1/2** (57% combined yield) further illustrates, how electron-withdrawing substituents negatively impact the yield of the reaction.

As evident from Scheme 5, the cyclization protocol is compatible with several functional groups (chloro, nitro, cyano, trifluoromethyl). In contrast, the reaction to give the hydroxyfluorenone **10t** (Scheme 6) was unsuccessful, suggesting that here TBHP chemoselectively reacts with the phenolic group to generate non-identifiable products. In order to provide an access to phenolic fluorenones as well, some commonly used phenol protecting groups were tested. Both TBS and SEM protecting groups were tolerated, as demonstrated by the syntheses of the fluorenones **10u** and **10v** (52 and 46% yields). As expected, the *O*-benzyl group was not tolerated, giving only trace amounts of product **10w**, as benzyl ethers are well known to undergo side reactions with free-radical reagents [62]. An extremely poor yield was further obtained with methylenedioxy substrate **15p**.

Our application of this new protocol to the first total synthesis of the natural product nobilone (**1d**) is depicted in Scheme 7. The commercially available phenol **16** was TBS-protected to give compound **17** in nearly quantitative yield according to known protocols [63]. Although *O*-unprotected hydroxyphenylboronic acids similar to **18** have been reported in the literature [64], we found the synthesis of the more lipophilic *O*-TBS-protected boronic acid **18** to proceed more facile in a yield of 66% via an aryllithium intermediate. For the synthesis of the benzylamine unit, commercially available nitrile **19** was reacted with NBS to regioselectively introduce a bromo substituent to give bromobenzonitrile **20** in a yield of 51% [65]. Biaryl **21** was constructed via a Suzuki coupling [57] of **18** and **20** in 41% yield. As expected, the TBS ether was cleaved under the Suzuki conditions and had to be reapplied [63] to give bis-TBS-protected cyanobiaryl **22** in 85% yield. Next, the nitrile group was reduced to the corresponding primary amine **23** in 80% yield, using LAH and AlCl_3_ [60]. The target compound nobilone (**1d**) was obtained via TBHP-mediated cyclization of **23** and subsequent TBS-deprotection of intermediate **24** with pyridine and HF·pyridine complex [66] in a total yield of 26% over the two steps. The longest linear sequence was 7 steps, with an overall yield of 5%.

**Scheme 8:**
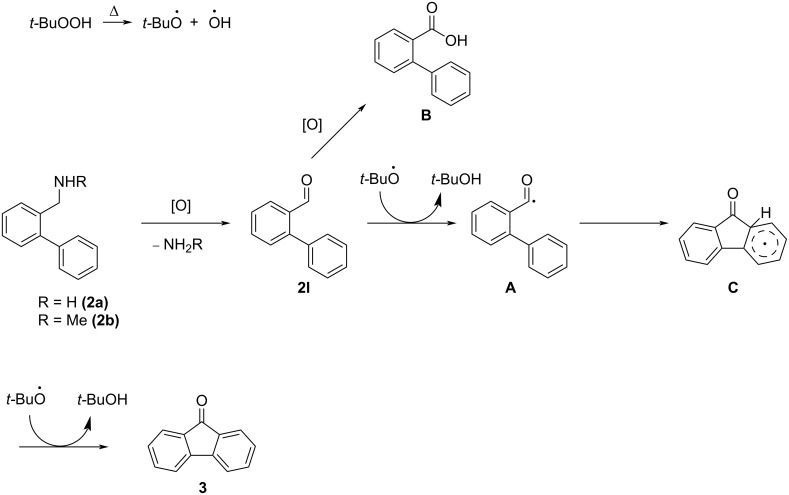
Proposed mechanism for the oxidative cyclization of amines **2a** and **2b** to fluorenone (**3**).

Finally, the reaction mechanism of the oxidative cyclization was explored. As a radical mechanism appeared very likely, the standard reaction with aqueous TBHP was run in the presence of the radical quenchers TEMPO and BHT (4 equivalents respectively) in separate experiments. While trapping a radical species of interest in form of a TEMPO ester was unsuccessful, fluorenone (**3**) was not formed under either of these conditions, suggesting the involvement of radicals in the cyclization reaction rather than an S_E_Ar mechanism as proposed in Scheme 3. A more detailed investigation might be required to fully understand the mechanism of this oxidative benzylamine cyclization, however, a tentative mechanism is proposed in Scheme 8, based on our observations as well as mechanistic investigations detailed in previous reports concerning similar TBHP-promoted cyclizations of aromatic aldehydes [26,35]. Notably, the two major side products of this reaction (detected by TLC using authentic references) are the aldehyde **2l** and, to a lesser extent, the carboxylic acid **B**, whereas formation of proposed intermediate aminofluorene **5** or other stable reaction intermediates was not observed. This suggests that benzylamine **2** is first oxidized by TBHP to aldehyde **2l** prior to cyclization. From aldehyde **2l** an acyl radical should subsequently be formed, for which cyclization reactions with heteroarenes and benzenoids are well documented [67–68]. Aldehyde **2l** may also be further oxidized to give carboxylic acid **B** instead. With the oxidant of choice, aqueous TBHP, however, aldehyde **2l** gives only a low cyclization yield (Table 1, entry 12). It is unclear, if water is directly involved with this mechanism, but studies indicate that water may lower the activation barrier for the radical cyclization owing to its solvent effect, as has been previously reported for the radical synthesis of cyclic lactones [69]. The intramolecular cyclization of the acyl radical **A** gives the tricyclic radical species **C**, which then forms fluorenone (**3**) after abstraction of a hydrogen radical by an additional equivalent of the *tert*-butoxy radical.

## Conclusion

In summary, we have demonstrated that the metal-free, TBHP-mediated radical cyclization is applicable to a wide variety of primary and secondary benzylamines bearing electron-donating and electron-withdrawing groups to synthesize fluorenones in poor to good yields, and utilized this method for the first total synthesis of the fluorenone natural product nobilone (**25**) in 8 steps in an overall yield of 2%. This protocol allows the synthesis of variously substituted fluorenones, since the required 2-(aminomethyl)biphenyls are readily available from commercially available building blocks (substituted benzonitriles or benzaldehydes, areneboronic acids) via Suzuki cross-coupling reactions, followed by reduction or reductive amination. The oxidative cyclization conditions are compatible with many functional groups on the aromatic rings (methoxy, chloro, cyano, nitro, and phenol protecting groups like TBS and SEM – but not benzyl and methylenedioxy). Electronic effects propagated by substituents were observed to have an influence on the reaction. In general, electron-withdrawing groups on the radical accepting arene had adverse effects, while electron donating groups, depending on the substitution pattern, had either a positive or negative effect of varying degree on the overall yield. This new protocol was utilized for the first total synthesis of the natural fluorenone nobilone (**1d**).

## Supporting Information

File 1 Additional experimental details (synthesis of the intermediates) and copies of NMR spectra.
