# Correction: Synthesis of highly substituted fluorenones via metal-free TBHP-promoted oxidative cyclization of 2-(aminomethyl)biphenyls. Application to the total synthesis of nobilone

**DOI:** 10.3762/bjoc.20.16

**Published:** 2024-01-30

**Authors:** Ilya A P Jourjine, Lukas Zeisel, Jürgen Krauß, Franz Bracher

**Affiliations:** 1 Department of Pharmacy - Center for Drug Research, Ludwig-Maximilians University of Munich, Butenandtstraße 5–13, 81377 Munich, Germany

**Keywords:** cross-dehydrogenative coupling, cyclization, fluorenones, nobilone, total synthesis

We noticed a number of minor errors in our original publication, including in Supporting Information File 1, that mostly concern the percent yield given for some of the precursors. In addition, there was a larger error related to the yield for the key step of the total synthesis of the title compound nobilone (**1d**). Finally, it should be noted that the sesquihydrate of K_2_CO_3_ rather than anhydrous K_2_CO_3_ was used.

In the original publication, we stated that the TBHP-mediated cyclization of compound **23** (929 mg, 1.96 mmol) to give compound **24** and the subsequent deprotection of crude compound **24** to give the title compound **1d** (275 mg, 1.14 mmol) was achieved in a percent yield of 26% over two steps. However, this does not match the amounts of substance given for amine **23** and nobilone (**1d**), respectively. The yield should be 58% instead of 26%.

All required corrections for the original publication are listed in detail below.

## Main Article

The required corrections for the original main article are listed in Table 1.

**Table 1:** Required corrections for the original main article.

instance	original	correction	reason

caption of Scheme 4	[…] a) Pd(PPh_3_)_4_ (5 mol %), Na_2_CO_3_, DMF/H_2_O, 18 h, 100 °C, 76–99% […].	[…] a) Pd(PPh_3_)_4_ (5 mol %), Na_2_CO_3_, DMF/H_2_O, 18 h, 100 °C, 76–97% […].	The percent yields stated for **8b** and **8d** were erroneous. They should be 96% and 97%, respectively, instead of 99%.
caption of Scheme 6	[…] a) Pd(PPh_3_)_4_ (5 mol %), Na_2_CO_3_, DMF/H_2_O, 18 h, 100 °C, 76–99%; b) LAH, AlCl_3_, THF, 18 h, rt […].	a) Pd(PPh_3_)_4_ (5 mol %), Na_2_CO_3_, DMF/H_2_O, 18 h, 100 °C, 70–93%; b) LAH, AlCl_3_, THF, 18 h, rt, 56–92% […].	The percent yield range of 76–99% for step a was mistakenly copied from the caption of Scheme 4 (step a) without adjustment to compounds **14p**–**w**. Further, the percent yield range for step b was mistakenly not provided.
page 2676	The target compound nobilone (**1d**) was obtained via TBHP-mediated cyclization of **23** and subsequent TBS deprotection of intermediate **24** with pyridine and HF·pyridine complex [66] in a total yield of 26% over the two steps.	The target compound nobilone (**1d**) was obtained via TBHP-mediated cyclization of **23** and subsequent TBS deprotection of intermediate **24** with pyridine and HF·pyridine complex [66] in a yield of 58% over the two steps.	—
page 2676	The longest linear sequence was 7 steps, with an overall yield of 5%.	The longest linear sequence was 7 steps. However, the overall yield of 5% for **1d** is based on a total of 8 steps required for the synthesis.	—
note in Scheme 7	**1d** (26% over 2 steps)	**1d** (58% over 2 steps)	—
Page 2677	[…] and utilized this method for the first total synthesis of the fluorenone natural product nobilone (**25**) in 8 steps in an overall yield of 2%.	[…] and utilized this method for the first total synthesis of the fluorenone natural product nobilone (**25**) in an overall yield of 5% in 8 steps.	—

## Supporting Information File 1

### 1.3 Compounds

The required corrections for the original Supporting Information File 1 are listed in Table 2.

**Table 2:** Required corrections for the original Supporting Information File 1.

instance	original	correction

several (for example, page S25)	example: K_2_CO_3_ (256 mg, 1.55 mmol, 1.00 equiv) and […] were added.	example: K_2_CO_3_·1.5H_2_O (256 mg, 1.55 mmol, 1.00 equiv) and […] were added.
2,7-dihydroxy-4-methoxy-9*H*-fluorenone (nobilone, **1d**)	A solution of crude ketone **24** (777 mg, 1.65 mmol) […]. and pyridine (0.61 mL, 7.59 mmol) […]. Purification by FCC afforded the product as a red solid (275 mg, 1.14 mmol, 26%).	A solution of crude ketone **24** (777 mg) […] and pyridine (0.610 mL, 7.59 mmol) […]. Purification by FCC afforded the product as a red solid (275 mg, 1.14 mmol, 58% over two steps).
4-methoxy-2-phenylbenzaldehyde (**8b**)	Purification by FCC afforded the product as a red solid (421 mg, 1.98 mmol, 99%).	Purification by FCC afforded the product as a red solid (421 mg, 1.98 mmol, 96%).
4-methoxy-2-phenylbenzaldehyde (**8d**)	Purification by FCC afforded the product as a red oil (423 mg, 1.99 mmol, 99%).	Purification by FCC afforded the product as a red oil (423 mg, 1.99 mmol, 97%).
5-methoxy-2-phenyl-*N*-methylbenzyl- amine (**9b**)	Purification by FCC afforded the product as a yellow oil (178 mg, 0.780 mmol, 72%).	Purification by FCC afforded the product as a yellow oil (178 mg, 0.780 mmol, 75%).
2-(3’,5’-dimethoxyphenyl)-*N*-methyl- benzylamine (**9c**)	Purification by FCC afforded the product as a yellow oil (139 mg, 0.696 mmol, 87%).	Purification by FCC afforded the product as a yellow oil (139 mg, 0.541 mmol, 87%).
2-(3’,5’-dimethoxyphenyl)benzonitrile (**14c**)	Purification by FCC afforded the product as a white solid (187 mg, 0.88 mmol, 88%).	Purification by FCC afforded the product as a white solid (1.39 g, 5.81 mmol, 88%).
2-(4’-methyl-3’-nitrophenyl)-*N*-(*tert*- butoxycarbonyl)benzylamine (**14o**)	Purification by FCC afforded the product as a white solid (416 mg, 1.21 mmol, 67%)	Purification by FCC afforded the product as a white solid (416 mg, 1.21 mmol, 61%)
2-(benzo[*d*][1,3]dioxol-5-yl)benzonitrile (**14p1**)	Purification by FCC afforded the product as a white solid (357 mg, 1.74 mmol, 80%)	Purification by FCC afforded the product as a white solid (357 mg, 1.61 mmol, 80%)

