# Supplementary material for: Synthesis of highly substituted fluorenones via metal-free TBHP-promoted oxidative cyclization of 2-(aminomethyl)biphenyls. Application to the total synthesis of nobilone
Source: Beilstein J Org Chem. 2021 Nov 2;17:2668–79. doi: 10.3762/bjoc.17.181 (PMC8576822; doi:10.3762/bjoc.17.181)
Supplement: File 1 — Additional experimental details (synthesis of the intermediates) and copies of NMR spectra. [file Beilstein_J_Org_Chem-17-2668-s001.pdf]

BEILSTEIN JOURNAL OF ORGANIC CHEMISTRY

Supporting Information

for

Synthesis of highly substituted fluorenones via metal-free TBHP-promoted oxidative cyclization of 2-(aminomethyl)biphenyls. Application to the total synthesis of nobilone

Ilya A. P. Jourjine, Lukas Zeisel, Jürgen Krauß and Franz Bracher

Beilstein J. Org. Chem. **2021,** *17,* 2668–2679. doi:10.3762/bjoc.17.181

Additional experimental details (synthesis of the intermediates) and copies of NMR spectra

License and Terms: This is a supporting information file under the terms of the Creative Commons Attribution License (https://creativecommons.org/licenses/by/4.0). Please note that the reuse, redistribution and reproduction in particular requires that the author(s) and source are credited and that individual graphics may be subject to special legal provisions.
The license is subject to the *Beilstein Journal of Organic Chemistry* terms and conditions: (https://www.beilstein-journals.org/bjoc/terms)

Table of contents

1. Experimental section page S2

2. ^{1}H and ^{13}C-NMR spectra page S49

3. References page S75

1. Experimental section

1.1 General reagent and analytical information

All solvents used were purified according to standard procedures or of HPLC or p.a. grade purchased from commercial sources. Chemical reagents were purchased from Sigma Aldrich (Schnelldorf, Germany) and TCI (Eschborn, Germany). IR spectra were recorded on a Perkin Elmer FTIR Paragon 1000 spectrometer. Melting points were determined on a Büchi melting point B-450 apparatus. NMR spectra were recorded on Jeol JNMR-GX 400 (400 MHz), Jeol JNMR-GX 500 (500 MHz), Avance III HD Bruker BioSpin (400 MHz) and Avance III HD Bruker BioSpin (500 MHz) spectrometers. Spectra were recorded in deuterated solvents and signal assignments were carried out based on ^{1}H, ^{13}C, DEPT, HMQC, HMBC, and COSY spectra. Chemical shifts are reported in parts per million (ppm) and J values are reported in hertz. Gas chromatography (GC) was performed on a Varian 3800 gas chromatograph coupled to a Saturn 2200 ion trap from Varian (Darmstadt, Germany). The autosampler was from CTC Analytics (Zwingen, Switzerland) and the split/splitless injector was a Varian 1177 (Darmstadt, Germany). Instrument control and data analysis were carried out with Varian Workstation 6.9 SP1 software (Darmstadt, Germany). High-resolution mass spectra were performed by electrospray ionization (ESI) using a Thermo Finnigan LTQ FT Ultra spectrometer or electron impact (EI) at 70 eV on a Jeol GCmate II or on a Finnigan MAT 95 spectrometer. All reactions were monitored by GC/MS or thin-layer chromatography (TLC) using precoated plastic sheets POLYGRAM®SIL G/UV254 from Macherey-Nagel (Düren, Germany). Compounds on TLC plates were detected under UV light at 254 and 366 nm. Chromatographic purification of products was performed by using a flash column chromatography (FCC) system from Büchi with a C-620 control unit combined with a C-660 fraction collector and a C-630 UV monitor. This system was controlled by the chromatography software SpeacoreControl 1.2.4000.0, standard edition. Separations were performed on Merck silica gel 60 as stationary phase.

1.2. General procedures

1.2.1. General procedure 1: Synthesis of (substituted) biphenyl-2-carboxaldehydes by Suzuki cross-coupling

To a mixture of boronic acid **7** (1.5 equiv) and bromobenzaldehyde **6** (1.0 equiv) a solution of $Pd(PPh_3)_4$ (0.050 equiv) in DMF/H_2O 1:1 (10 mL per mmol bromobenzaldehyde) was added under N_2 atmosphere, followed by an aqueous solution of Na_2CO_3 (0.3 M, 3.0 equiv). The resulting heterogeneous mixture was refluxed for 16 h before it was diluted with H_2O and extracted with EtOAc. The combined organic layers were separated, washed with brine, dried over $MgSO_4$, and evaporated *in vacuo*. The crude product was purified by FCC.

1.2.2. General procedure 2: Synthesis of *N*-methyl-2-(aminomethyl)biphenyls by reductive amination

Methylamine (10 equiv) was added to a solution of biphenyl-2-carboxaldehyde **8** (1.0 equiv) in DCM (10 mL per mmol carboxaldehyde). The solution was stirred at room temperature for 3 h after which $NaBH_4$ (2.0 equiv) was added and the mixture was stirred for an additional 6 h. After completion of the reaction

H_2O was added and the mixture was extracted with DCM. The combined organic layers were washed with brine, dried over $MgSO_4$ and the solvent was evaporated *in vacuo*. The crude product was purified by FCC.

1.2.3. General procedure 3: Synthesis of 9-fluorenones

Aqueous TBHP (70%, 4.0 equiv) was added to a solution of primary amine **15** or secondary amine **9** (1.0 equiv) in 1,2-DCE (4 mL per mmol amine) in a vial lined with a teflon cap and the resulting mixture was heated to 100 °C for 18 h. The solvent was evaporated *in vacuo* and the crude product purified by FCC without further workup.

1.2.4. General procedure 4: Synthesis of biphenyl-2-carbonitriles and biphenyl-2-ylmethylaminoformic acid *tert*-butyl esters by Suzuki cross-coupling

Boronic acid **7** (1.5 equiv), 2-bromobenzonitrile **12** or *N*-Boc-benzylamine **13** (1.0 equiv), $Pd(PPh_3)_4$ (0.050 equiv), and Na_2CO_3 (3.0 equiv) were dissolved in a mixture of H_2O/DMF 1:1 (10 mL per mmol *o*-bromobenzonitrile or *o*-bromo-*N*-Boc-benzylamine under a N_2 atmosphere. The resulting heterogeneous mixture was refluxed for 18 h, before it was diluted with H_2O. The mixture was extracted with EtOAc, the combined organic layers washed with brine, dried over $MgSO_4$, and concentrated *in vacuo*. The crude product was purified by FCC.

1.2.5. General procedure 5: Synthesis of 2-phenylbenzylamines by reduction of nitriles

$AlCl_3$ (1.5–4.0 equiv) was carefully added to a stirred suspension of LAH (1.5–4.0 equiv) in anhydrous THF at 0 °C. The reaction mixture was then stirred vigorously for 20 min before dropwise adding a solution of biphenyl-2-carbonitrile **14** (1.0 equiv) in THF. After complete addition, stirring was continued for 16 h. In case the reaction was not complete (TLC control), additional LAH and $AlCl_3$ were added. After completion of the reaction it was quenched by slowly adding H_2O. The mixture was concentrated *in vacuo*. 1 M NaOH was added to the residue and stirred until a white solid formed. Then, the mixture was filtered through a pad of silica gel, and the filtrate was extracted with EtOAc and washed with H_2O. The combined organic layers were dried over $MgSO_4$ and concentrated *in vacuo*. The crude product was purified by FCC.

1.2.6. General procedure 6: Synthesis of *tert*-butyldimethylsilyl ethers of phenols

Imidazole (2.5 equiv) and TBS-Cl (1.2 equiv) were added to a solution of phenol **14t**, **16** or **21** (1.0 equiv) in DMF at 50 °C and stirred for 16 h. The progress of the reaction was monitored by TLC. After completion of the reaction the mixture was diluted with Et_2O and the organic layer was separated from the aqueous layer. The aqueous layer was extracted with Et_2O and the combined organic layers were washed with H_2O, LiCl solution (5%) and brine, dried over $MgSO_4$ and concentrated *in vacuo*. The crude product was purified by FCC.

1.2.7. General procedure 7: Deprotection of the *N*-Boc protecting group

Trifluoroacetic acid (TFA, 26–32 equiv) was added to a solution of Boc-protected amine **14k**, **14m** or **14o** (1.0 equiv) dissolved in DCM and the mixture was stirred for 6 h at room temperature. After completion of the reaction the solvent was evaporated *in vacuo*. Aqueous NaOH (1 M) was added to the residue, followed by extraction with EtOAc. The combined organic layers were washed with H_2O, brine, dried over $MgSO_4$ and the solvent removed *in vacuo*. The crude product was purified by FCC.

1.3 Compounds

2,7-Dihydroxy-4-methoxy-9*H*-fluorenone (nobilone) (**1d**)

A solution of crude ketone **24** (777 mg, 1.65 mmol), Olah's reagent (pyridine·HF, 0.650 mL, 7.26 mmol) and pyridine (0.61 mL, 7.59 mmol) in EtOAc (20 mL) was stirred at room temperature overnight. The reaction was quenched with methoxytrimethylsilane (7.93 mL, 57.8 mmol) and stirred for 40 min. The solvent was evaporated *in vacuo*. Purification by FCC afforded the product as a red solid (275 mg, 1.14 mmol, 26%).

R_f = 0.48 (hexanes/EtOAc 2:1); Mp = 263 °C; IR (ATR): 3295, 2922, 2852, 1715, 1670, 1599, 1463, 1444, 1313, 1235, 1196, 1155, 1131, 1064, 1027, 959, 908, 880, 832, 790, 779 cm^{-1}; δ_H(400 MHz, DMSO-d_6): 9.83 (2H, s, OH), 7.37 (1H, d, *J* = 8.0 Hz, 5-H), 6.87 (1H, d, *J* = 2.3 Hz, 8-H), 6.82 (1H, dd, *J* = 8.0, 2.5 Hz, 6-H), 6.59 – 6.54 (2H, m, 3-H and 1-H), 3.85 (3H, s, OCH_3). δ_C(101 MHz, DMSO-d_6): 193.6 (CO), 159.4 (2-COH), 156.8 (7-COH), 155.2 (C-4), 135.8 (C-9a), 135.0 (C-5a), 134.5 (C-8a), 123.5 (C-5), 122.2 (C-4a'), 120.8 (C-6), 111.3 (C-8), 105.4 (C-3), 103.5 (C-1), 55.6 (OCH_3); HRMS (EI): m/z $[M]^{•+}$ calcd for $C_{14}H_{10}O_4^{•+}$ 242.0579; found 242.0566. Data in accordance with literature values.[1]

2-Phenylbenzylamine (**2a**)[2]

This compound was prepared in accordance with **General procedure 5** from nitrile **2m** (1.32 g, 7.36 mmol), LAH (838 mg, 22.1 mmol) and $AlCl_3$ (2.94 g, 22.1 mmol). Purification by FCC afforded the product as a yellow oil (1.19 g, 6.49 mmol, 88%).

R_f = 0.35 (hexanes/EtOAc 1:1 + NEt_3 1%); IR (ATR): 3353, 3286, 3057, 3020, 2929, 2869, 1593, 1476, 1449, 1435, 1343, 1333, 1069, 1008, 914, 891, 774, 759, 749, 700 cm^{-1}; δ_H(400 MHz, DMSO-d_6): 7.58 (1H, dd, *J* = 7.7, 1.4 Hz, 6-H), 7.47 – 7.40 (2H, m, 2'-H and 6'-H or 3'-H and 6'-H), 7.39 – 7.32 (4H, m, 2'-H and 6'-H and H_{arom} or 3'-H and 6'-H and H_{arom}), 7.28 (1H, td, *J* = 7.4, 1.5 Hz, 4-H), 7.17 (1H, dd, *J* = 7.5, 1.5 Hz, 3-H), 3.62 (2H, s, $C\underline{H_2}NH_2$); δ_C(101 MHz, DMSO-d_6): 141.1 (C-2), 140.9 (C-1 or C-1'), 140.4 (C-1 or C-1'), 129.4 (C-3), 129.0 (C-2' and C-6' or C-3' and C-5'), 128.2 (C-2' and C-6' or C-3' and C-5'), 128.2 (C-6), 127.4 (C-5), 127.0 (C-4'), 126.2 (C-4), 43.2 (CH_2NH_2); HRMS (EI): m/z $[M]^{•+}$ calcd for $C_{13}H_{13}N^{•+}$ 183.1048; found 183.1049.

2-Phenyl-*N*-methylbenzylamine (**2b**)[3]

This compound was prepared in accordance with **General procedure 2** from biphenyl-2-carboxaldehyde **2l** (0.581 mL, 3.60 mmol), methylamine (5.68 mL, 128 mmol) and $NaBH_4$ (272 mg, 7.20 mmol). Purification by FCC afforded the product as a white solid (555 mg, 2.81 mmol, 78%).

R_f = 0.41 (hexanes/EtOAc 1:1 + NEt_3 1%); Mp = 55-57 °C; IR = 3277, 3058, 3023, 2843, 2788, 1597, 1476, 1435, 1353, 1130, 1096, 1009, 745, 700 cm^{-1}; δ_H(500 MHz, DMSO-*d*$_6$): 7.53 (1H, dd, *J* = 7.4, 1.5 Hz, 6-H), 7.46 – 7.35 (5H, m, H_{arom}), 7.35 – 7.32 (1H, m, 5-H), 7.30 (1H, td, *J* = 7.4, 1.6 Hz, 4-H), 7.20 (1H, dd, *J* = 7.5, 1.5 Hz, 3-H), 3.53 (2H, s,$C\underline{H}_2N$), 2.19 (3H, s, $NHC\underline{H}_3$); δ_C(126 MHz, DMSO-*d*$_6$): 141.1 (C-2), 140.8 (C-1'), 137.7 (C-1), 129.5 (C-3), 129.0 (C-2' and C-6' or C-3' and C-5'), 128.8 (C-6), 128.1 (C-2' and C-6' or C-3' and C-5'), 127.2 (C-5 or C-4'), 127.0 (C-4), 126.6 (C-5 or C-4'), 52.7 ($\underline{C}H_2NH$), 35.9 ($NH\underline{C}H_3$); HRMS (EI): m/z [M - H]$^-$ calcd for $C_{14}H_{14}N^-$ 196.1132; found 196.1121.

1-([1,1'-Biphenyl]-2-ylmethyl)pyrrolidine **(2d)**

To a solution of 2-phenylbenzyl bromide (0.183 mL, 1.00 mmol, 1.00 equiv) in DCM (5 mL) pyrrolidine (0.167 mL, 2.0 mmol, 2.00 equiv) and triethylamine (0.279 mL, 2.00 mmol, 2.00 equiv) were added. After 3 h aqueous NaOH (5%, 5 mL) was added and the aqueous layer was extracted with DCM (3 x 5 mL). The combined organic layers were washed with brine (3 x 5 mL), dried over $MgSO_4$ and the solvent removed *in vacuo*. Purification afforded the product as a colorless oil (186 mg, 0.780 mmol, 78%).

R_f = 0.16 (hexanes/EtOAc 4:1); IR = 3058, 3022, 2965, 2906, 2873, 2786, 2734, 2362, 2342, 1965, 1598, 1478, 1452, 1375, 1348, 1324, 1290, 1249, 1200, 1141, 1010, 941, 883, 752, 703 cm^{-1}; δ_H(400 MHz, CD_2Cl_2): 7.56 (dd, *J* = 7.3, 1.8 Hz, 1H, 6-H), 7.47 – 7.41 (m, 4H, H_{arom}), 7.41 – 7.39 (m, 1H, H_{arom}), 7.38 – 7.35 (m, 1H, H_{arom}), 7.35 – 7.30 (m, 1H, H_{arom}), 7.29 – 7.24 (m, 1H, H_{arom}), 3.54 (s, 2H, CH_2N), 2.47 – 2.40 (m, 4H, $N(C\underline{H}_2)_2(CH_2)_2$), 1.72 (dq, *J* = 6.4, 3.2 Hz, 4H, $N(CH_2)_2(C\underline{H}_2)_2$); δ_C(101 MHz, CD_2Cl_2): 142.6 (C-2), 142.14 (C-1‘), 137.8 (C-1), 130.5 (C-3), 130.3 (C-6), 130.2 (C-3‘ and C-5‘), 128.4 (C-2‘ and C-6‘), 127.7 (C_{arom}), 127.3 (C_{arom}), 127.1 (C_{arom}), 57.94 (CH_2N), 54.4 ($N(\underline{C}H_2)_2(CH_2)_2$), 24.1 ($NH(CH_2)_2(\underline{C}H_2)_2$); HRMS (EI): m/z [M-H]$^{\bullet+}$ calcd for $C_{17}H_{19}N$ 237.1517; found 237.1533.

2-Phenylbenzonitrile (**2m**)[4]

R_f = 0.43 (hexanes/EtOAc 10:1); This compound was prepared in accordance with **General procedure 4** from 2-bromobenzonitrile **12** (1.46 g, 8.00 mmol), phenylboronic acid **7** (1.46 g, 12.0 mmol), $Pd(PPh_3)_4$ (462 mg, 0.400 mmol) and Na_2CO_3 (2.54 g, 24.0 mmol). Purification by FCC afforded the product as a white solid (1.32 mg, 7.36 mmol, 92%).

Mp = 37 °C; IR (ATR): 3062, 3051, 3031, 3017, 2220, 1595, 1475, 1450, 1432, 1270, 1188, 1166, 1075, 1008, 922, 777, 753, 732, 700 cm^{-1}; δ_H(400 MHz, $CDCl_3$): 7.77 (1H, dd, *J* = 7.8, 1.4 Hz, 6-H), 7.65 (1H, td, *J* = 7.7, 1.4 Hz, 4-H), 7.57 (2H, dd, *J* = 8.2, 1.6 Hz, 2'-H and 6'-H or 3'-H and 5'-H), 7.54 – 7.48 (3H, m, 2'-H and 6'-H and H_{arom} or 3'-H and 5'-H and H_{arom}), 7.48 – 7.42 (2H, m, H_{arom}); δ_C(101 MHz, $CDCl_3$): 145.65 (C-2), 138.28 (C-1'), 133.89 (C-6), 132.94 (C-4), 130.22 (C-5 or C-3 or C-4'), 128.95 – 128.79 (m,

C-2’ and C-3’ and C-5’ and C-6’ and C_{arom}), 127.67 (C-5 or C-3 or C-4’), 118.85 (CN), 111.45 (C-1); HRMS (EI): m/z $[M]^{•+}$ calcd for $C_{13}H_9N^{•+}$ 179.0735; found 179.0727.

9*H*-Fluoren-9-one (**3**)

This compound was prepared in accordance with **General procedure 3** from different starting materials:

a) from amine **2a** (134 mg, 0.730 mmol) and aqueous TBHP (70%, 0.404 mL, 2.92 mmol). Purification by FCC afforded the product as a yellow solid (83.0 mg, 0.461 mmol, 62%).
b) from amine **2a** (119 mg, 0.650 mmol) and TBHP in decane (80%, 0.315 mL, 2.60 mmol). Purification by FCC afforded the product as a yellow solid (26.3 mg, 0.146 mmol, 22%).
c) from amine **2b** (90.7 mg, 0.460 mmol) and aqueous TBHP (70%, 0.255 mL, 1.84 mmol). Purification by FCC afforded the product as a yellow solid (50.0 mg, 0.277 mmol, 60%).
d) from amine **2d** (133 mg, 0.560 mmol) and aqueous TBHP (70%, 0.310 mL, 2.24 mmol). Purification by FCC afforded the product as a yellow solid (13.5 mg, 0.075 mmol, 13%).
e) from alcohol **2k** (92.1 mg, 0.500 mmol) and aqueous TBHP (70%, 0.277 mL, 2.00 mmol). Purification by FCC afforded the product as a yellow solid (23.2 mg, 0.128 mmol, 26%).
f) from alcohol **2k** (97.6 mg, 0.530 mmol) and TBHP in decane (80%, 0.257 mL, 2.12 mmol). Purification by FCC afforded the product as a yellow solid (57.0 mg, 0.316 mmol, 60%).
g) from aldehyde **2l** (87.5 mg, 0.480 mmol) and aqueous TBHP (70%, 0.266 mL, 1.92 mmol). Purification by FCC afforded the product as a yellow solid (21.9 mg, 0.122 mmol, 25%).
h) from aldehyde **2l** (95 mg, 0.520 mmol) and TBHP in nonane (5.5 M, 0.378 mL, 2.08 mmol). Purification by FCC afforded the product as a yellow solid (24 mg, 0.133 mmol, 26%).

R_f = 0.46 (hexanes/EtOAc 12:1); Mp = 81-82 °C; IR (ATR): 2365, 2343, 1715, 1611, 1599, 1450, 1299, 1193, 1151, 1098, 919, 736, 671 cm^{-1}; δ_H (400 MHz, CD_2Cl_2): δ = 7.63 (ddd, J = 7.3, 1.2, 0.8 Hz, 2H, 1-H and 8-H), 7.57 (ddd, J = 7.5, 1.2, 0.8 Hz, 2H, 4-H and 5-H), 7.51 (td, J = 7.4, 1.2 Hz, 2H, 3-H and 6-H), 7.31 (dd, J = 7.4, 1.2 Hz, 2H, 2-H and 7-H); δ_C (101 MHz, CD_2Cl_2): δ = 194.0 (C-9), 144.8 (C-4a and C-5a), 135.1 (C-3 and C-6), 134.5 (C-8a and C-9a), 129.5 (C-2 and C-7), 124.4 (C-1 and C-8), 120.8 (C-4 and C-5); HRMS (EI): m/z $[M]^{•+}$ calcd for $C_{13}H_8O$ 180.0575$^{•+}$; found 180.0569. Data in accordance with literature values.[5]

2-(3’,4’,5’-Trimethoxyphenyl)benzaldehyde (**8a**)[6]

This compound was prepared in accordance with **General procedure 1** from 2-formylphenylboronic acid **7** (225 mg, 1.50 mmol), 5-bromo-1,2,3-trimethoxybenzene **6** (247 mg, 1.00 mmol), $Pd(PPh_3)_4$ (58 mg, 0.050 mmol) and Na_2CO_3 (318 mg, 3.00 mmol). Purification by FCC afforded the product as a white solid (226 mg, 0.830 mmol, 83%).

R_f = 0.34 (hexanes/EtOAc 4:1); Mp = 92-93 °C; IR (ATR): 2967, 2942, 2369, 2346, 1683, 1454, 1410, 1346, 1295, 1237, 1123, 997, 840, 760 cm^{-1}; δ_H (400 MHz, CD_2Cl_2): δ = 9.99 (d, J = 0.9 Hz, 1H, CHO), 7.97 (ddd, J = 7.7, 1.5, 0.9 Hz, 1H, 6-H), 7.64 (td, J = 7.5, 1.5 Hz, 1H, 4-H), 7.53 – 7.45 (m, 2H, 5-H and

3-H), 6.59 (s, 2H, 2‘-H and 6‘-H), 3.85 (s, 6H, 3’-OCH_3 and 5’-OCH_3), 3.83 (s, 3H, 4’-OCH_3); δ_C (101 MHz, CD_2Cl_2): δ = 192.1 (CHO), 153.17 (C-3‘ and C-5‘), 146.0 (C-2), 138.0 (C-4‘), 133.9 (C-1 and C-1‘), 133.4 (C-4), 130.6 (C-3), 127.7 (C-5), 127.1 (C-6), 108.1 (C-2‘ and C-6‘), 60.5 (C-4’-OCH_3), 56.2 (3’-OCH_3 and 5’-OCH_3); HRMS (ESI): m/z $[M]^{\bullet+}$ calcd for $C_{16}H_{16}O_4{}^{\bullet+}$ 272.1049; found 272.1044.

4-Methoxy-2-phenylbenzaldehyde (**8b**)[7]

R_f = 0.73 (hexanes/EtOAc 4:1); This compound was prepared in accordance with **General procedure 1** from phenylboronic acid **7** (377 mg, 3.09 mmol), 2-bromo-5-methoxybenzaldehyde **6** (443 mg, 2.06 mmol), $Pd(PPh_3)_4$ (116 mg, 0.103 mmol) and Na_2CO_3 (655 mg, 6.18 mmol). Purification by FCC afforded the product as a red solid (421 mg, 1.98 mmol, 99%).

Mp = 79-81 °C; IR (ATR): 3014, 2979, 2936, 2845, 2753, 1679, 1607, 1482, 1286, 1233, 1050, 938, 776 cm^{-1}; δ_H (400 MHz, CD_2Cl_2) δ = 9.82 (s, 1H, CHO), 7.99 (d, J = 8.7 Hz, 1H, 3-H), 7.51 – 7.44 (m, 3H, 3‘-H and 4‘-H and 5‘-H), 7.43 – 7.38 (m, 2H, 2‘-H and 6‘-H), 7.02 (ddd, J = 8.7, 2.6 Hz, 1H, 4-H), 6.90 (d, J = 2.6 Hz, 1H, 6-H), 3.90 (s, 3H, OCH_3); δ_C (101 MHz, CD_2Cl_2) δ = 191.1 (CHO), 164.1 (C-5), 149.0 (C-1), 138.5 (C-2), 130.5 (C-3‘ and C-5‘), 130.3 (C-3), 128.8 (C-2‘ and C-6‘), 128.7 (C-4‘), 127.9 (C-1‘), 115.8 (C-6), 114.5 (C-4), 56.3 (CHO); HRMS (EI): m/z $[M]^{\bullet+}$ calcd for $C_{14}H_{12}O_2{}^{\bullet+}$ 212.0837; found 212.0831.

2-(3’,5’-Dimethoxyphenyl)benzaldehyde (**8c**)[6]

This compound was prepared in accordance with **General procedure 1** from 2-formylphenylboronic acid **7** (225 mg, 1.50 mmol), 5-bromo-1,3-dimethoxybenzene **6** (217 mg, 1.00 mmol), $Pd(PPh_3)_4$ (58 mg, 0.050 mmol) and Na_2CO_3 (318 mg, 3.00 mmol). Purification by FCC afforded the product as a white solid (185 mg, 0.764 mmol, 76%).

R_f = 0.52 (hexanes/EtOAc 4:1); Mp = 77-78 °C; IR (ATR): 3000, 2963, 2940, 1965, 1596, 1463, 1206, 1159, 1065, 769 cm^{-1}; δ_H (400 MHz, CD_2Cl_2): δ = 9.99 (d, J = 0.8 Hz, 1H, 7-H), 7.97 (ddd, J = 7.5 Hz, 1.5 Hz, 0.6 Hz, 1H, 6-H), 7.64 (td, J = 7.5, 1.5 Hz, 1H, 4-H), 7.53 – 7.48 (m, 1H, 5-H), 7.46 (ddd, J = 7.5, 1.3, 0.6 Hz, 1H, 3-H), 6.54 (dd, J = 2.2 Hz, 1H, 4‘-H), 6.52 (d, J = 2.2 Hz, 2H, 2‘-H and 6‘-H), 3.82 (s, 6H, OCH_3); δ_C (101 MHz, CD_2Cl_2): δ = 192.6 (CHO), 161.3 (C-3’ and C-5’), 146.4 (C-2), 140.5 (C-4’), 134.4 (C-5), 134.0 (C-1), 131.0 (C-1’), 128.4 (C-4), 127.7 (C-3), 109.0 (C-3 and C-4), 100.4 (C-2’ and C’-6’), 56.0 (3’-OCH_3 and 5’-OCH_3); HRMS (EI): m/z $[M]^{\bullet+}$ calcd for $C_{15}H_{14}O_3{}^{\bullet+}$ 242.0943; found 242.0936.

4-Methoxy-2-phenylbenzaldehyde (**8d**)[7]

This compound was prepared in accordance with **General procedure 1** from phenylboronic acid **7** (377 mg, 3.09 mmol), 2-bromo-4-methoxybenzaldehyde **6** (443 mg, 2.06 mmol), $Pd(PPh_3)_4$ (116 mg, 0.100 mmol) and Na_2CO_3 (655 mg, 6.18 mmol). Purification by FCC afforded the product as a red oil (423 mg, 1.99 mmol, 99%).

R_f = 0.67 (hexanes/EtOAc 4:1); IR (ATR): 2942, 2841, 2759, 1688, 1601, 1486, 1300, 1254, 1227, 1178, 1123, 1034, 883, 770, 706 cm^{-1}; δ_H (400 MHz, CD_2Cl_2): δ = 9.93 (s, 1H, CHO), 7.49 (d, J = 2.8 Hz, 1H, 6-H), 7.47 – 7.43 (m, 2H, 2‘-H and 6‘-H), 7.41 (d, J = 6.4 Hz, 1H, 3-H), 7.39 – 7.37 (m, 2H, 3‘-H and 5‘-H),

7.36 (dd, J = 1.9, 1.3 Hz, 1H, 4'-H), 7.22 (dd, J = 8.5, 2.8 Hz, 1H, 5-H), 3.90 (s, 3H, OCH_3); δ_C (101 MHz, CD_2Cl_2) δ = 192.5 (CHO), 159.8 (C-4), 139.5 (C-2), 138.2 (C-1'), 135.2 (C-1), 132.7 (C-6), 130.8 (C-3' and C-5'), 128.9 (C-2' and C-6'), 128.3 (C-4'), 121.5 (C-5), 110.6 (C-3), 56.2 (CHO); HRMS (ESI): m/z [M - H]$^-$ calcd for $C_{14}H_{11}O_2^-$ 211.0756; found 211.0752.

2-(3',4',5'-Trimethoxyphenyl)-*N*-methylbenzylamine (**9a**)

This compound was prepared in accordance with **General procedure 2** from aldehyde **8a** (196 mg, 0.720 mmol), methylamine (4.00 mL, 36.0 mmol) and $NaBH_4$ (41.0 mg, 1.08 mmol). Purification by FCC afforded the product as a yellow oil (179 mg, 0.623 mmol, 87%).

R_f = 0.55 (hexanes/EtOAc 1:1); IR (ATR): 2970, 2834, 2789, 2362, 2342, 1584, 1512, 1484, 1463, 1410, 1344, 1240, 1129, 468 cm^{-1}; δ_H (400 MHz, CD_2Cl_2): δ = 7.45 – 7.42 (m, 1H, 6-H), 7.35 – 7.31 (m, 1H, 5-H), 7.31 – 7.28 (m, 1H, 4-H), 7.28 – 7.27 (m, 1H, 3-H), 6.69 (s, 2H, 2'-H and 6'-H), 3.84 (s, 6H,3'-OCH_3 and 5'-OCH_3), 3.82 (s, 3H, 4'-OCH_3), 3.66 (d, J = 0.6 Hz, 2H, $C\underline{H}_2NH$), 2.36 (s, 3H, $NHC\underline{H}_3$); δ_C (101 MHz, CD_2Cl_2): δ = 153.5 (C-3' and C-5'), 142.5 (C-2), 138.3 (C-1), 137.7 (C-4'), 137.4 (C-1'), 130.4 (C-3), 129.9 (C-6), 127.9 (C-5), 127.4 (C-4), 107.0 (C-2' and C-6'), 61.0 (4'-OCH_3), 56.6 (3'-OCH_3 and 5'-OCH_3), 54.2 (CH_2NH), 36.6 ($CH_2NH\underline{C}H_3$); HRMS (ESI): m/z [M - H]$^-$ calcd for $C_{17}H_{18}NO_3^-$ 286.1449; found 286.1456.

5-Methoxy-2-phenyl-*N*-methylbenzylamine (**9b**)

This compound was prepared in accordance with **General procedure 2** from aldehyde **8b** (221 mg, 1.04 mmol), methylamine (5.77 mL, 52.0 mmol) and $NaBH_4$ (79.0 mg, 2.08 mmol). Purification by FCC afforded the product as a yellow oil (178 mg, 0.780 mmol, 72%).

R_f = 0.18 (hexanes/EtOAc 1:1); IR (ATR): 3056, 3027, 2965, 2836, 2790, 1608, 1484, 1444, 1274, 1231, 1165, 1052, 769, 706 cm^{-1}; δ_H (500 MHz, CD_2Cl_2): δ = 7.40 – 7.39 (m, 1H, 2'-H or 6'-H), 7.39 – 7.38 (m, 1H, 2'-H or 6'-H), 7.35 – 7.34 (m, 2H, 3'-H and 5'-H), 7.34 – 7.32 (m, 1H, 4'-H), 7.16 (d, J = 8.4 Hz, 1H, 3-H), 7.05 (d, J = 2.7 Hz, 1H, 6-H), 6.84 (dd, J = 8.4, 2.7 Hz, 1H, 4-H), 3.84 (s, 3H, OCH_3), 3.64 (s, 2H, $C\underline{H}_2N$), 2.30 (s, 3H, $NHC\underline{H}_3$); δ_C (126 MHz, CD_2Cl_2): δ = 159.6 (C-5), 141.7 (C-1'), 139.7 (C-1), 134.8 (C-2), 131.6 (C-3), 129.9 (C-3' and C-5'), 128.6 (C-2' and C-6'), 127.2 (C-4'), 114.7 (C-6), 112.7 (C-4), 55.8 (OCH_3), 53.8 (CH_2NH), 36.4 ($NHCH_3$); HRMS (ESI): m/z [M - H]$^-$ calcd for $C_{15}H_{14}NO^-$ 226.1237; found 226.1248.

2-(3',5'-Dimethoxyphenyl)-*N*-methylbenzylamine (**9c**)

This compound was prepared in accordance with **General procedure 2** from aldehyde **8c** (150 mg, 0.619 mmol), methylamine (3.44 mL, 31.0 mmol) and $NaBH_4$ (47.0 mg, 1.24 mmol). Purification by FCC afforded the product as a yellow oil (139 mg, 0.696 mmol, 87%).

R_f = 0.30 (hexanes/EtOAc 1:1); IR (ATR): 2970, 2838, 2789, 2360, 1596, 1462, 1420, 1351, 1205, 1157, 1065, 1030, 847, 766 cm^{-1}; δ_H (500 MHz, CD_2Cl_2): δ = 7.45 (dd, J = 7.3, 1.4 Hz, 1H, 6-H), 7.33 (td, J = 7.3, 1.7 Hz, 1H, 5-H), 7.28 (td, J = 7.3, 1.4 Hz, 1H, 4-H), 7.25 (dd, J = 7.3, 1.7 Hz, 1H, 3-H), 6.55 (d, J =

2.3 Hz, 2H, 2‘-H and 6‘-H), 6.46 (t, J = 2.3 Hz, 1H, 4‘-H), 3.80 (s, 6H, OCH_3), 3.66 (s, 2H, C$\underline{H}_2$NH), 2.33 (s, 3H, NHC$\underline{H}_3$); δ_C (126 MHz, CD_2Cl_2): δ = 161.1 (OCH_3), 143.9 (C-1‘), 142.4 (C-2), 138.3 (C-1), 130.2 (C-3), 129.7 (C-6), 128.0 (C-5), 127.3 (C-4), 107.8 (C-2‘ and C-6‘), 99.6 (C-4‘), 55.9 (OCH_3), 54.0 (CH_2NH), 36.5 (NH$\underline{C}H_3$); HRMS (ESI): m/z $[M - H]^-$ calcd for $C_{16}H_{16}NO_2^-$ 256.1343; found 256.1324.

4-Methoxy-2-phenyl-*N*-methylbenzylamine (**9d**)

This compound was prepared in accordance with **General procedure 2** from aldehyde **8d** (221 mg, 1.04 mmol), methylamine (5.77 mL, 52.0 mmol) and $NaBH_4$ (79.0 mg, 2.08 mmol). Purification by FCC afforded the product as a yellow oil (185 mg, 0.814 mmol, 78%).

R_f = 0.24 (hexanes/EtOAc 1:1); IR (ATR): 2953, 2836, 2789, 1607, 1569, 1486, 1296, 1221, 1176, 1041, 769, 705 cm^{-1}; δ_H (400 MHz, CD_2Cl_2): δ = 7.47 – 7.28 (m, 6H, 2‘-H and 3‘-H and 6-H and 4‘-H and 5‘-H and 6‘-H), 6.88 (dd, J = 8.4, 2.8 Hz, 1H, 5-H), 6.79 (d, J = 2.8 Hz, 1H, 3-H), 3.80 (s, 3H, OCH_3), 3.60 (s, 2H, C$\underline{H}_2$NH), 2.27 (s, 3H, NHC$\underline{H}_3$); δ_C (101 MHz, CD_2Cl_2): δ = 158.8 (C-4), 143.7 (C-1), 141.8 (C-2), 131.1 (C_{arom}), 129.9 (C_{arom}), 129.6 (C_{arom} and C_{arom}), 128.7 (C_{arom} and C_{arom}), 127.7 (C_{arom}), 115.8 (C-3), 113.5 (C-5), 55.8 (OCH_3), 53.0 (CH_2NH), 36.0 (NHCH_3); HRMS (ESI): m/z $[M - H]^-$ calcd for $C_{15}H_{16}NO^-$ 226.1237; found 226.1224.

1,2,3-Trimethoxy-9*H*-fluoren-9-one (**10a**)

This compound was prepared in accordance with **General procedure 3** from

a) primary amine **15a** (120 mg, 0.440 mmol) and aqueous TBHP (70%, 0.244 mL, 1.76 mmol). Purification by FCC afforded the product as a yellow solid (61.0 mg,0.226 mmol, 51%).
b) secondary amine **9a** (90.2 mg, 0.314 mmol) and aqueous TBHP (70%, 0.174 mL, 1.26 mmol). Purification by FCC afforded the product as a yellow solid (58.0 mg, 0.215 mmol, 68%).

R_f = 0.27 (hexanes/EtOAc 4:1); Mp = 100-101 °C; IR (ATR): 3006, 2980, 2944, 2838, 1705, 1607, 1590, 1485, 1465, 1412, 1377, 1254, 1208, 1136, 975, 760 cm^{-1}; δ_H (500 MHz, CD_2Cl_2): δ = 7.54 (dt, J = 7.3, 0.9 Hz, 1H, 8-H), 7.48 – 7.45 (m, 2H, 5-H and 6-H), 7.27 (ddd, J = 7.3, 5.6, 2.7 Hz, 1H, 7-H), 6.89 (s, 1H, 4-H), 4.07 (s, 3H, 1-OCH_3), 3.98 (s, 3H, 3-OCH_3), 3.81 (s, 3H, 2-OCH_3); δ_C (126 MHz, CD_2Cl_2): δ = 190.8 (C-9), 160.0 (C-3), 154.0 (C-1), 143.5 (C-5a), 142.7 (C-4a), 142.5 (C-2), 135.8 (C-13), 134.4 (C-6), 129.3 (C-7), 123.9 (C-8), 120.0 (C-5), 118.5 (C-9a), 100.7 (C-4), 62.5 (1-OCH_3), 61.7 (2-OCH_3), 57.0 (3-OCH_3); HRMS (EI): m/z $[M]^{\bullet+}$ calcd for $C_{16}H_{14}O_4^{\bullet+}$ 270.0892; found 270.0885. Data in accordance with literature values.[8]

2-Methoxy-9*H*-fluoren-9-one (**10b)**

This compound was prepared in accordance with **General procedure 3** from

a) primary amine **15b-1** (113 mg, 0.530 mmol) and aqueous TBHP (70%, 0.293 mL, 2.12 mmol). Purification by FCC afforded the product a yellow solid (45.0 mg, 0.214 mmol, 40%).
b) primary amine **15b-2** (102 mg, 0.480 mmol) and aqueous TBHP (70%, 0.266 mL, 1.92 mmol). Purification by FCC afforded the product as a yellow solid (38.0 mg, 0.181 mmol, 38%).

c) secondary amine **9b** (132 mg, 0.581 mmol) and aqueous TBHP (0.322 mL, 2.33 mmol). Purification by FCC afforded the product as a yellow solid (41.0 mg, 0.195 mmol, 34%).

R_f = 0.38 (hexanes/EtOAc 10:1); Mp = 72-73 °C; IR (ATR): 2998, 2972, 2837, 1717, 1604, 1491, 1465, 1289, 1233, 1038, 953, 927, 735 cm^{-1}; δ_H (400 MHz, CD_2Cl_2): 7.56 (1H, dt, *J* = 7.4, 1.0 Hz, 8-H), 7.48 – 7.42 (3H, m, 4-H and 5-H and 6-H), 7.21 (1H, ddd, *J* = 7.4, 6.0, 2.6 Hz, 7-H), 7.17 (1H, d, *J* = 2.5 Hz, 1-H), 7.00 (1H, dd, *J* = 8.2, 2.5 Hz, 4-H), 3.85 (3H, s, OCH_3); δ_C (101 MHz, CD_2Cl_2): 194.1 (C-9), 161.5 (C-2), 145.4 (C-5a), 137.4 (C-4a), 136.5 (C-9a), 135.4 (C-6), 134.9 (C-8a), 128.4 (C-7), 124.6 (C-8), 122.0 (C-4), 120.5 (C-3), 120.2 (C-5), 109.9 (C-1), 56.3 (OCH_3); HRMS (EI): m/z $[M]^{\bullet+}$ calcd for $C_{14}H_{10}O_2^{\bullet+}$ 210.0681; found 210.0674. Data in accordance with literature values.[9]

1,3-Dimethoxy-9*H*-fluoren-9-one (**10c**)

This compound was prepared in accordance with **General procedure 3** from

a) primary amine **15c** (77.9 mg, 0.320 mmol) and aqueous TBHP (70 %, 0.177 mL, 1.28 mmol). Purification by FCC afforded the product as a yellow solid (70.4 mg, 0.290 mmol, 92%).
b) secondary amine **9c** (43.0 mg, 0.167 mmol) and aqueous TBHP (70%, 0.0925 mL, 0.668 mmol). Purification by FCC afforded the product as a yellow solid (24.0 mg, 0.100 mmol, 60%).

R_f = 0.29 (hexanes/EtOAc 2:1); Mp = 141-142 °C; IR (ATR): 2972, 2874, 1697, 1615, 1602, 1466, 1380, 1308, 1211, 1147, 1029, 768 cm^{-1}; δ_H (500 MHz, CD_2Cl_2): 7.55 (dt, *J* = 7.3, 1.1 Hz, 1H, 8-H), 7.52 – 7.49 (m, 1H, 5-H), 7.46 (td, *J* = 7.4, 1.1 Hz, 1H, 6-H), 7.31 (td, *J* = 7.3, 1.1 Hz, 1H, 7-H), 6.74 (d, *J* = 1.9 Hz, 1H, 4-H), 6.31 (d, *J* = 1.9 Hz, 1H, 2-H), 3.94 (s, 3H, 3-OCH_3), 3.92 (s, 3H, 1-OCH_3); δ_C (126 MHz, CD_2Cl_2): 190.5 (C-9), 168.0 (C-3), 160.6 (C-1), 149.0 (C-4a), 142.9 (C-5a), 136.2 (C-8a), 133.9 (C-6), 129.9 (C-7), 123.6 (C-8), 120.6 (C-5), 114.4 (C-9a), 100.2 (C-4), 98.5 (C-2), 56.5 (1-OCH_3), 56.4 (3-OCH_3); HRMS (EI): m/z $[M]^{\bullet+}$ calcd for $C_{15}H_{12}O_3^{\bullet+}$ 240.0786; found 240.0782. Data in accordance with literature values.[10]

3-Methoxy-9*H*-fluoren-9-one (**10d**)

This compound was prepared in accordance with **General procedure 3** from

a) amine **9d** (132 mg, 0.581 mmol) and aqueous TBHP (0.322 mL, 2.33 mmol). Purification by FCC afforded the product as a yellow solid (41.0 mg, 0.195 mmol, 34%).
b) from amine **15f** (177 mg, 0.830 mmol) and aqueous TBHP (70%, 0.460 mL, 3.32 mmol). Purification by FCC (eluent: isohexane/ethyl acetate 8:1) afforded the product a yellow solid (73.0 mg, 0.347 mmol, 42%). Here **10d** was accompanied by its regioisomer 1-methoxy-9*H*-fluoren-9-one **(10f**), obtained as a yellow solid (26.0 mg, 0.124 mmol, 15%).

10d: R_f = 0.32 (hexanes/EtOAc 4:1); Mp = 93-95 °C; IR (ATR): $\tilde{\nu}$ = 3060, 2926, 2842, 2362, 2342, 1704, 1608, 1598, 1488, 1440, 1299, 1237, 1182, 1097, 1021, 920, 832, 768, 736 cm^{-1}; δ_H (400 MHz, CD_2Cl_2): 7.61 – 7.56 (m, 2H, 1-H and 8-H), 7.55 – 7.52 (m, 1H, 5-H), 7.49 (td, *J* = 7.3, 1.2 Hz, 1H, 6-H), 7.31 (td, *J*

= 7.3, 1.2 Hz, 1H, 7-H), 7.08 (d, *J* = 2.3 Hz, 1H, 4-H), 6.77 (dd, *J* = 8.3, 2.3 Hz, 1H, 2-H), 3.91 (s, 3H, OCH_3); δ_C (101 MHz, CD_2Cl_2): 192.7 (C-9), 166.1 (C-3), 147.6 (C-4a), 143.9 (C-5a), 135.9 (C-8a), 134.7 (C-7), 129.9 (C-6), 127.6 (C-9a), 126.5 (C-8), 124.1 (C-1), 120.8 (C-5), 113.7 (C-2), 107.6 (C-4), 56.4 (OCH_3); HRMS (EI): m/z $[M]^{•+}$ calcd for $C_{14}H_{10}O_2^{•+}$ 210.0681; found 210.0676. Data in accordance with literature values.[11]

10f: R_f = 0.29 (hexanes/EtOAc 10:1); Mp = 132-134 °C, IR (ATR): 3060, 2923, 2842, 1698, 1607, 1590, 1487, 1454, 1438, 1362, 1292, 1232, 1180, 1152, 1094, 1019, 915, 873, 831, 765, 732, 673 cm^{-1}; δ_H(400 MHz, CD_2Cl_2): 7.58 (1H, ddd, *J* = 7.3, 1.2, 0.7 Hz, 8-H), 7.54 (1H, dt, *J* = 7.4, 0.9 Hz, 5-H), 7.52 – 7.44 (2H, m, 3-H and 6-H), 7.31 (1H, td, *J* = 7.4, 1.1 Hz, 7-H), 7.17 (1H, dd, *J* = 7.3, 0.6 Hz, 4-H), 6.87 (1H, dd, *J* = 8.5, 0.7 Hz, 2-H), 3.96 (3H, s, OCH_3); δ_C(101 MHz, CD_2Cl_2): 191.8 (C-9), 158.7 (C-1), 146.8 (C-4a), 143.6 (C-5a), 137.2 (C-3), 134.9 (C-8a), 134.3 (C-6), 129.6 (C-7), 123.9 (C-8), 120.7 (C-5), 120.4 (C-9a), 113.6 (C-2), 113.3 (C-4), 56.2 (OCH_3); HRMS (EI): m/z $[M]^{•+}$ calcd for $C_{14}H_{10}O_2^{•+}$ 210.0681; found 210.0674. Data in accordance with literature values.[12]

4-Methoxy-9*H*-fluoren-9-one (**10e**)

This compound was prepared in accordance with **General procedure 3** from amine **15e** (75.0 mg, 0.350 mmol) and aqueous TBHP (70%, 0.194 mL, 1.40 mmol). Purification by FCC afforded the product an orange solid (38.0 mg, 0.181 mmol, 52%).

R_f = 0.36 (hexanes/EtOAc 10:1); Mp = 100-103 °C; IR (ATR): 3010, 2924, 2854, 1716, 1602, 1490, 1454, 1424, 1298, 1272, 1246, 1200, 1141, 1034, 1017, 964, 863, 834, 761, 728, 212, cm^{-1}; δ_H(400 MHz, CD_2Cl_2): 7.56 (1H, dt, *J* = 7.3, 1.0 Hz, 1-H), 7.47 – 7.42 (3H, m, 3-H and 4-H and 7-H), 7.21 (1H, ddd, *J* = 7.3, 5.9, 2.5 Hz, 2-H), 7.17 (1H, d, *J* = 2.5 Hz, 8-H), 7.00 (1H, dd, *J* = 8.2, 2.5 Hz, 6-H), 3.85 (3H, s, OCH_3); δ_C(101 MHz, CD_2Cl_2): 194.0 (C-9), 161.5 (C-5), 145.4 (C-4a), 137.4 (C-5a), 136.5 (C-8a or C-9a), 135.4 (C-3), 134.9 (C-8a or C-9a), 128.4 (C-2), 124.6 (C-1), 122.0 (C-4 or C-7), 120.5 (C-6), 120.2 (C-4 or C-7), 109.9 (C-8), 56.3 (OCH_3); HRMS (EI): m/z $[M]^{•+}$ calcd for $C_{14}H_{10}O_2^{•+}$ 210.0681; found 210.0674. Data in accordance with literature values.[13]

4-(Trifluoromethyl)-9*H*-fluoren-9-one (**10g**)

This compound was prepared in accordance with **General procedure 3** from amine **15g** (50.3 mg, 0.200 mmol) and aqueous TBHP (70%, 0.111 mL, 0.800 mmol). Purification by FCC afforded the product as a yellow solid (14.0 mg, 0.0564 mmol, 28%).

R_f = 0.52 (hexanes/EtOAc 10:1); Mp = 122-124 °C; IR (ATR): 2923, 2853, 1716, 1607, 1578, 1466, 1424, 1327, 1303, 1258, 1171, 1156, 1114, 1089, 1070, 932, 886, 829, 737, 718 cm^{-1}; δ_H(500 MHz, CD_2Cl_2): 7.90 – 7.84 (2H, m, 2-H and 1-H), 7.80 (1H, dd, *J* = 7.9, 1.0 Hz, 3-H), 7.73 (1H, dt, *J* = 7.5, 1.0 Hz, 8-H), 7.58 (1H, td, *J* = 7.7, 1.4 Hz, 6-H), 7.45 (1H, ddd, *J* = 8.0, 7.3, 0.9 Hz, 5-H), 7.42 (1H, td, *J* = 7.4, 0.9 Hz, 7-H); δ_C(126 MHz, CD_2Cl_2): 192.6 (C-9), 142.7 (t, *J* = 2.2 Hz, C-4a), 142.0 (C-5a), 136.3 (C-9a), 135.9 (C-6), 134.6 (C-8a), 131.8 (q, *J* = 5.7 Hz, C-3), 130.7 (C-7), 129.7 (C-5), 127.7 (C-1), 125.1 (q, *J* = 5.6 Hz, C-

2), 124.8 (C-8), 124.6 (q, *J* = 32.8 Hz, C-4), 124.3 (q, *J* = 272.3, CF_3); HRMS (EI): m/z $[M]^{•+}$ calcd for $C_{14}H_7F_3O^{•+}$ 248.0449; found 248.0442. Data in accordance with literature values.[14]

2-(Trifluoromethyl)-9*H*-fluoren-9-one (**10h**)

This compound was prepared in accordance with **General procedure 3** from amine **15h** (146 mg, 0.580 mmol) and aqueous TBHP (70%, 0.321 mL, 2.32 mmol). Purification by FCC afforded the product as a yellow solid (26.0 mg, 0.105 mmol, 18%).

R_f = 0.40 (hexanes/EtOAc 10:1); Mp = 130-132 °C; IR (ATR): 2922, 2853, 1716, 1622, 1606, 1466, 1458, 1325, 1266, 1146, 1107, 1054, 907, 849, 815, 769, 736, 656 cm^{-1}.; δ_H(500 MHz, CD_2Cl_2): 7.89 – 7.87 (1H, m, 1-H), 7.80 (1H, ddd, *J* = 7.8, 1.8, 0.9 Hz, 3-H), 7.72 – 7.68 (2H, m, 8-H and 4-H), 7.66 (1H, dt, *J* = 7.5, 0.9 Hz, 5-H), 7.59 (1H, td, *J* = 7.5, 1.2 Hz, 6-H), 7.41 (1H, td, *J* = 7.5, 1.1 Hz, 7-H); δ_C(126 MHz, CD_2Cl_2): 192.3 (C-9), 148.0 (C-4a), 143.5 (C-5a), 135.6 (C-6), 135.0 (C-8a or C-9a), 134.7 (C-8a or C-9a), 132.0 (q, *J* = 3.9 Hz, C-3), 131.4 (q, *J* = 32.8 Hz, C-2), 130.7 (C-7), 124.9 (C-8 or C-4), 124.3 (q, *J* = 272.2 Hz, CF_3), 121.7 (C-5), 121.4 (q, *J* = 3.8 Hz, C-1), 121.1 (C-8 or C-4); HRMS (EI): m/z $[M]^{•+}$ calcd for $C_{14}H_7F_3O^{•+}$ 248.0449; found 248.0443. Data in accordance with literature values.[15]

1-(Trifluoromethyl)-9*H*-fluoren-9-one (**10i1**) and 3-(trifluoromethyl)-9*H*-fluoren-9-one (**10i2**)

These compounds were prepared in accordance with **General procedure 3** from amine **10i** (130 mg, 0.516 mmol) and aqueous TBHP (70%, 0.200 mL, 2.06 mmol). Purification by FCC (elution with isohexane/ethyl acetate 8:1) afforded **10i2** as a yellow solid (35.0 mg, 0.141 mmol, 27%), followed by **10i1**, a yellow solid (40.0 mg, 0.161 mmol, 31%).

10i1: R_f = 0.57 (hexanes/EtOAc 10:1); Mp = 124 °C; IR (ATR): 1718, 1608, 1592, 1318, 1294, 1267, 1172, 1136, 1116, 1108, 1083, 1065, 1038, 918, 800, 754, 717, 677 cm^{-1}; δ_H(500 MHz, CD_2Cl_2): 7.79 (1H, d, *J* = 7.4 Hz, 4-H), 7.67 (1H, dt, *J* = 7.4, 1.0 Hz, 8-H), 7.65 – 7.59 (2H, m, 3-H and 5-H), 7.58 – 7.54 (2H, m, 2-H and 6-H), 7.38 (1H, td, *J* = 7.4, 1.1 Hz, 7-H); δ_C(126 MHz, CD_2Cl_2): 190.3 (C-9), 146.9 (C-4a), 143.3 (C-5a), 135.4 (C-6), 135.0 (C-3), 133.7 (C-8a), 131.2 (C-9a), 130.4 (C-7), 127.6 (q, *J* = 35.2 Hz, C-1), 126.3 (q, *J* = 5.7 Hz, C-2), 124.9 (C-8), 124.2 (C-4), 123.5 (q, *J* = 274.6 Hz, CF_3), 120.9 (C-5); HRMS (EI): m/z $[M]^{•+}$ calcd for $C_{14}H_7F_3O^{•+}$ 248.0449; found 248.0444. Data in accordance with literature values.[14]

10i2: R_f = 0.31 (hexanes/EtOAc 10:1); Mp = 133-135 °C; IR (ATR): 3058, 2922, 1712, 1624, 1600, 1478, 1423, 1319, 1303, 1268, 1165, 1115, 1053, 919, 844, 762, 744, 682, 663 cm^{-1}; δ_H(500 MHz, CD_2Cl_2): 7.80 (1H, t, *J* = 0.8 Hz, 4-H), 7.74 (1H, dt, *J* = 7.8, 0.8 Hz, 1-H), 7.69 (1H, dt, *J* = 7.4, 1.0 Hz, 8-H), 7.64 (1H, dt, *J* = 7.5, 1.0 Hz, 5-H), 7.60 (1H, ddd, *J* = 7.7, 1.6, 0.8 Hz, 2-H), 7.58 (1H, td, *J* = 7.4, 1.1 Hz, 6-H), 7.39 (1H, td, *J* = 7.4, 1.1 Hz, 7-H); δ_C(126 MHz, CD_2Cl_2): 192.7 (C-9), 145.4 (C-4a), 143.6 (C-5a), 137.2 (C-9a), 136.3 (q, *J* = 32.6 Hz, C-3), 135.7 (C-6), 134.4 (C-8a), 130.4 (C-7), 126.7 (q, *J* = 4.1 Hz, C-2), 124.9 (C-1), 124.6 (C-8), 124.1 (q, *J* = 273.2 Hz, CF_3), 121.3 (C-5), 117.7 (q, *J* = 3.8 Hz, C-4); HRMS (EI): m/z $[M]^{•+}$ calcd for $C_{14}H_7F_3O^{•+}$ 248.0449; found 248.0443. Data in accordance with literature values.[16]

2-Chloro-9*H*-fluoren-9-one (**10j**)

This compound was prepared in accordance with **General procedure 3** from amine **15j** (107 mg, 0.490 mmol) and aqueous TBHP (70%, 0.271 mL, 1.96 mmol). Purification by FCC afforded the product as a yellow solid (27.0 mg, 0.126 mmol, 26%).

R_f = 0.49 (hexanes/EtOAc 10:1); Mp = 119-121 °C; IR (ATR): 3049, 2923, 2853, 2360, 2341, 1706, 1615, 1599, 1449, 1417, 1299, 1258, 1190, 1113, 1063, 947, 879, 832, 800, 759, 728, 669 cm^{-1}; δ_H(400 MHz, CD_2Cl_2): 7.64 (1H, dt, *J* = 7.4, 1.0 Hz, 8-H), 7.58 (1H, dd, *J* = 1.9, 0.7 Hz, 1-H), 7.55 – 7.52 (2H, m, 5-H and 6-H), 7.50 (1H, d, *J* = 0.7 Hz, 3-H), 7.48 (1H, d, *J* = 1.8 Hz, 4-H), 7.33 (1H, ddd, *J* = 7.3, 6.6, 2.0 Hz, 7-H); δ_C(101 MHz, CD_2Cl_2): 192.2 (C-9), 143.6 (C-5a), 142.6 (C-4a), 135.7 (C-2 or C-9a), 135.1 (C-6), 134.9 (C-8a), 134.2 (C-3), 133.9 (C-2 or C-9a), 129.3 (C-7), 124.3 (C-1 and C-8), 121.6 (C-4), 120.6 (C-5); HRMS (EI): m/z $[M]^{•+}$ calcd for $C_{13}H_7{}^{35}ClO^{•+}$ 214.0185; found 214.0179. Data in accordance with literature values.[8]

9-Oxo-9*H*-fluorene-2-carbonitrile (**10k**)

R_f = 0.35 (hexanes/EtOAc 4:1); This compound was prepared in accordance with **General procedure 3** from amine **15k** (154 mg, 0.740 mmol) and aqueous TBHP (70%, 0.410 mL, 2.96 mmol). Purification by FCC afforded the product as a yellow solid (20.0 mg, 0.0974 mmol, 13%).

Mp = 170-171 °C; IR (ATR): 3089, 3049, 2922, 2853, 2233, 1713, 1604, 1457, 1292, 1178, 1105, 963, 928, 854, 765, 725, 689 cm^{-1}; δ_H(400 MHz, CD_2Cl_2): 7.88 (1H, dd, *J* = 1.5, 0.7 Hz, 1-H), 7.82 (1H, dd, *J* = 7.7, 1.5 Hz, 3-H), 7.72 (1H, dt, *J* = 7.4, 1.0 Hz, 8-H), 7.70 – 7.65 (2H, m, 4-H and 5-H), 7.60 (1H, td, *J* = 7.5, 1.2 Hz, 6-H), 7.44 (1H, td, *J* = 7.4, 1.1 Hz, 7-H); δ_C(101 MHz, CD_2Cl_2): 191.7 (C-9), 148.4 (C-4a), 143.2 (C-5a), 139.0 (C-3), 135.7 (C-6), 134.9 (C-9a), 134.6 (C-8a), 131.1 (C-7), 127.7 (C-1), 125.0 (C-8), 122.0 (C-5), 121.5 (C-4), 118.5 (CN), 113.0 (C-2); HRMS (EI): m/z $[M]^{•+}$ calcd for $C_{14}H_7NO^{•+}$ 205.0528; found 205.0520. Data in accordance with literature values.[14]

1,3,7-Trimethoxy-9*H*-fluoren-9-one (**10l**)

This compound was prepared in accordance with **General procedure 3** from amine **15l** (136 mg, 0.499 mmol) and aqueous TBHP (70%, 0.276 mL, 2.0 mmol). Purification by FCC afforded the product as a yellow solid (70.0 mg, 0.259 mmol, 52%).

R_f = 0.23 (hexanes/EtOAc 2:1); Mp = 174 °C; IR (ATR): 1696, 1595, 1430, 1297, 1207, 1015, 936, 824, 788 cm^{-1}; δ_H(400 MHz, CD_2Cl_2): 7.38 (1H, d, *J* = 8.2 Hz, 5-H), 7.10 (1H, d, *J* = 2.4 Hz, 8-H), 6.94 (1H, dd, *J* = 8.2, 2.4 Hz, 6-H), 6.62 (1H, d, *J* = 1.9 Hz, 4-H), 6.22 (1H, d, *J* = 1.9 Hz, 2-H), 3.92 (3H, s, 1-OCH_3), 3.90 (3H, s, 3-OCH_3), 3.84 (3H, s, 7-OCH_3); δ_C(101 MHz, CD_2Cl_2): 190.1 (C-9), 168.0 (C-3), 161.7 (C-7), 160.5 (C-1), 149.2 (C-4a or C-8a), 138.0 (C-4a or C-8a), 135.0 (C-5a), 121.5 (C-5), 118.9 (C-6), 114.3 (C-9a), 108.9 (C-8), 99.5 (C-4), 97.2 (C-2), 56.3 (1-OCH_3 or 3-OCH_3 or 7-OCH_3), 56.2 (1-OCH_3 or 3-OCH_3 or 7-OCH_3), 56.1 (1-OCH_3 or 3-OCH_3 or 7-OCH_3); HRMS (EI): m/z $[M]^{•+}$ calcd for $C_{16}H_{14}O_4{}^{•+}$ 270.0892; found 270.0887.

Phenanthridine (**10m**)

This compound was prepared accidentally in accordance with **General procedure 3** from amine **10m** (97.3 mg, 0.405 mmol) and aqueous TBHP (70%, 0.224 mL, 1.62 mmol). Purification by FCC gave phenanthridine as a white solid (26.0 mg, 0.145 mmol, 36%).

R_f = 0.30 (hexanes/EtOAc 4:1); Mp = 103-105 °C; IR (ATR): 2922, 2852, 1725, 1615, 1587, 1574, 1525, 1489, 1457, 1443, 1401, 1342, 1288, 1243, 1193, 1145, 1134, 1033, 969, 890, 861, 773, 746, 720 cm^{-1}; δ_H(400 MHz, CD_2Cl_2): 9.28 (1H, s, 6-H), 8.65 (1H, dd, *J* = 8.4, 1.1 Hz, 10-H), 8.62 (1H, dd, *J* = 8.0, 1.6 Hz, 1-H), 8.17 (1H, dd, *J* = 7.9, 1.5 Hz, 4-H), 8.08 (1H, dt, *J* = 7.9, 1.0 Hz, 7-H), 7.89 (1H, ddd, *J* = 8.4, 7.1, 1.4 Hz, 9-H), 7.79 – 7.68 (3H, m, 2-H and 3-H and 8-H); δ_C(101 MHz, CD_2Cl_2): 154.0 (C-6), 145.0 (C-4a), 132.8 (C-10a), 131.4 (C-9), 130.5 (C-4), 129.1 (C-2 or C-3 or C-8), 129.0 (C-7), 127.9 (C-2 or C-3 or C-8), 127.4 (C-2 or C-3 or C-8), 126.9 (C-6a), 124.4 (C-1a), 122.7 (C-1), 122.3 (C-10); HRMS (EI): m/z $[M]^{\bullet+}$ calcd for $C_{13}H_9N$ 179.0735; found 179.0727. Data in accordance with literature values.[17]

2,3-Dimethoxy-9*H*-fluoren-9-one (**10n1**)

This compound was prepared in accordance with **General procedure 3** from amine **15n** (122 mg, 0.500 mmol) and aqueous TBHP (70%, 0.277 mL, 2.00 mmol). Purification by FCC afforded the product as an orange solid (42.0 mg, 0.175 mmol, 35%).

R_f = 0.23 (hexanes/EtOAc 4:1); Mp = 158-160 °C; IR (ATR): 1703, 1587, 1499, 1461, 1413, 1365, 1324, 1287, 1262, 1242, 1208, 1160, 1113, 1023, 857, 763, 735, 713 cm^{-1}; δ_H(500 MHz, CD_2Cl_2): 7.50 (1H, dt, *J* = 7.3, 1.0 Hz, 8-H), 7.42 (1H, td, *J* = 7.3, 1.1 Hz, 6-H), 7.41 – 7.38 (1H, m, 5-H), 7.20 (1H, td, *J* = 7.3, 1.3 Hz, 7-H), 7.15 (1H, s, 1-H), 7.03 (1H, s, 4-H), 3.97 (3H, s, 2-OCH_3 or 3-OCH_3), 3.88 (3H, s, 2-OCH_3 or 3-OCH_3); δ_C(126 MHz, CD_2Cl_2): 193.3 (C-9), 155.4 (C-3), 150.6 (C-2), 144.6 (C-5a), 139.9 (C-4a), 135.4 (C-8a), 134.8 (C-6), 128.7 (C-7), 127.6 (C-9a), 123.9 (C-8), 119.7 (C-5), 107.6 (C-1), 104.2 (C-4), 56.8 (2-OCH_3 or 3-OCH_3), 56.7 (2-OCH_3 or 3-OCH_3); HRMS (EI): m/z $[M]^{\bullet+}$ calcd for $C_{15}H_{12}O_3^{\bullet+}$ 240.0786; found 240.0784. Data in accordance with literature values.[2]

2-Methyl-1-nitro-9*H*-fluoren-9-one (**10o1**) and 2-methyl-3-nitro-9*H*-fluoren-9-one (**10o2**)

These compounds were prepared in accordance with **General procedure 3** from amine **15o** (124 mg, 0.510 mmol) and aqueous TBHP (70%, 0.282 mL, 2.04 mmol). Purification by FCC (eluent: isohexane/ethyl acetate 8:1) afforded **10o2** as a yellow solid (15.0 mg, 0.0627 mmol, 12%), followed by **10o1** as a yellow solid (23.0 mg, 0.0961 mmol, 19%).

10o1: R_f = 0.30 (hexanes/EtOAc 4:1); Mp = 199 °C; IR (ATR): 2922, 2853, 1709, 1618, 1604, 1528, 1457, 1377, 1354, 1294, 1268, 1183, 1154, 968, 836, 812, 760, 750, 692 cm^{-1}, δ_H(400 MHz, CD_2Cl_2): 7.64 (1H, dt, *J* = 7.3, 1.0 Hz, 8-H), 7.60 – 7.52 (3H, m, 4-H, 5-H and 6-H), 7.47 (1H, dq, *J* = 7.6, 0.8 Hz, 3-H), 7.36 (1H, ddd, *J* = 7.4, 6.6, 1.9 Hz, 7-H), 2.30 (3H, d, *J* = 0.7 Hz, CH_3), δ_C(101 MHz, CD_2Cl_2): 189.1 (C-9), 143.8 (C-4a), 143.2 (C-5a), 138.2 (C-3), 135.7 (C-6), 133.8 (C-8a), 130.3 (C-7), 130.2 (C-1), 125.2 (C-8),

124.1 (C-9a and C-2), 122.4 (C-4), 121.2 (C-5), 16.4 (CH_3), HRMS (EI): m/z $[M]^{•+}$ calcd for $C_{14}H_9NO_3^{•+}$ 239.0582; found 239.0574.

10o2: R_f = 0.38 (hexanes/EtOAc 4:1); Mp = 209 °C; IR (ATR): 2922, 2853, 1714, 1602, 1519, 1449, 1339, 1297, 1179, 1120, 880, 816, 755, 734, 721 cm^{-1}; δ_H(400 MHz, CD_2Cl_2): 8.02 (1H, s, 4-H), 7.69 (1H, dt, *J* = 7.4, 1.0 Hz, 8-H), 7.63 – 7.56 (4H, m, 1-H and 5-H and 6-H), 7.39 (1H, td, *J* = 7.2, 1.7 Hz, 7-H), 2.58 (3H, s, CH_3); δ_C(101 MHz, CD_2Cl_2): 192.2 (C-9), 153.6 (C-3), 143.3 (C-4a or C-5a), 143.1 (C-4a or C-5a), 137.1 (C-9a), 136.0 (C-6), 135.2 (C-2), 134.5 (C-8a), 130.4 (C-7), 128.5 (C-1), 124.9 (C-8), 121.5 (C-5), 116.8 (C-4), 20.5 (CH_3); HRMS (EI): m/z $[M]^{•+}$ calcd for $C_{14}H_9NO_3^{•+}$ 239.0582; found 239.0575.

9*H*-Fluoreno[2,3-d][1,3]dioxol-9-one (**10p1**)

This compound was prepared in accordance with **General procedure 3** from amine **15p** (107 mg, 0.470 mmol) and aqueous TBHP (70%, 0.260 mL, 1.88 mmol). Purification by FCC afforded the product an orange solid (8.50 mg, 0.0379 mmol, 8%).

R_f = 0.30 (hexanes/EtOAc 10:1); Mp = 146-148 °C; IR (ATR): 2922, 2853, 1699, 1605, 1591, 1477, 1456, 1366, 1341, 1259, 1220, 1179, 1024, 926, 888, 866, 759, 728, 702 cm^{-1}; δ_H(400 MHz, CD_2Cl_2): 7.50 (1H, dt, *J* = 7.3, 1.0 Hz, 8-H), 7.43 (1H, td, *J* = 7.4, 1.2 Hz, 6-H), 7.36 (1H, dt, *J* = 7.3, 0.9 Hz, 5-H), 7.22 (1H, td, *J* = 7.4, 1.1 Hz, 7-H), 7.05 (1H, d, *J* = 0.5 Hz, 1-H), 7.00 (1H, d, *J* = 0.5 Hz, 4-H), 6.06 (2H, s, OCH_2O); δ_C(101 MHz, CD_2Cl_2): 192.4 (C-9), 153.8 (C-4), 149.0 (C-2), 144.0 (C-5a), 142.0 (C-4a), 135.1 (C-8a), 134.7 (C-6), 129.0 (C-9a), 128.7 (C-7), 123.8 (C-8), 119.7 (C-5), 105.1 (C-1), 102.9 (OCH_2O), 102.1 (C-5); HRMS (EI): m/z $[M]^{•+}$ calcd for $C_{14}H_8O_3^{•+}$ 224.0473; found 224.0467. Data in accordance with literature values.[18]

2-Methoxy-1-methyl-9*H*-fluoren-9-one (**10q1**) and 2-methoxy-3-methyl-9*H*-fluoren-9-one (**10q2**)

These compounds were prepared in accordance with **General procedure 3** from amine **15q** (111 mg, 0.490 mmol) and aqueous TBHP (70%, 0.271 mL, 1.96 mmol). Purification by FCC (eluent: isohexane/ethyl acetate 8:1) afforded **10q1** as a yellow solid (27.0 mg, 0.120 mmol, 25%) and **10q2** as a yellow solid (35 mg, 0.156 mmol, 32%).

10q1: R_f = 0.44 (hexanes/EtOAc 10:1); Mp = 144 °C; IR (ATR): 2922, 2853, 1706, 1597, 1458, 1378, 1364, 1300, 1261, 1197, 1110, 1077, 1041, 1018, 880, 860, 765, 738, 697 cm^{-1}; δ_H(400 MHz, CD_2Cl_2): 7.52 (1H, dt, *J* = 7.3, 1.0 Hz, C-8), 7.45 – 7.37 (2H, m, 5-H and 6-H), 7.31 (1H, s, 4-H), 7.19 (1H, td, *J* = 7.1, 1.5 Hz,7-H), 7.12 (1H, s, 1-H), 3.88 (3H, s, OCH_3), 2.27 (3H, d, *J* = 0.8 Hz, CH_3); δ_C(101 MHz, CD_2Cl_2): 194.0 (C-9), 159.3 (C-2), 145.3 (C-5a), 137.5 (C-4a), 135.0 (C-6), 134.9 (C-8a), 134.4 (C-3), 134.0 (C-9a), 128.1 (C-7), 124.1 (C-8), 123.1 (C-4), 119.8 (C-5), 106.0 (C-1), 56.1 (C-OCH_3), 17.4 (CH_3); HRMS (EI): m/z $[M]^{•+}$ calcd for $C_{15}H_{12}O_2$ 224.0837; found 224.0832.

10q2: R_f = 0.46 (hexanes/EtOAc 10:1); Mp = 129 °C; IR (ATR): 2957, 2922, 2853, 1693, 1598, 1460, 1436, 1376, 1297, 1262, 1230, 1190, 1111, 1067, 1014, 932, 820, 795, 762, 740, 719, 680 cm^{-1}; δ_H(400

MHz, CD_2Cl_2): 7.55 (1H, dt, *J* = 7.4, 1.0 Hz, 8-H), 7.46 – 7.43 (2H, m, 5-H and 6-H), 7.32 (1H, dd, *J* = 8.1, 4-H), 7.24 – 7.17 (1H, m, 7-H), 6.87 (1H, d, *J* = 8.1 Hz, 3-H), 3.86 (3H, s, OCH_3), 2.50 (3H, s, CH_3); δ_C(101 MHz, CD_2Cl_2): 195.3 (C-9), 159.8 (C-2), 144.6 (C-5a), 136.6 (C-4a), 135.1 (C-8a), 134.9 (C-6), 132.6 (C-9a), 129.8 (C-1), 128.0 (C-7), 124.0 (C-8), 119.8 (C-5), 118.7 (C-4), 114.2 (C-3), 56.4 (OCH_3), 10.2 (CH_3); HRMS (EI): m/z $[M]^{\bullet+}$ calcd for $C_{15}H_{12}O_2^{\bullet+}$ 224.0837; found 224.0832.

2-Phenyl-9*H*-fluoren-9-one (**10r**)

This compound was prepared in accordance with **General procedure 3** from amine **15r** (207 mg, 0.800 mmol) and aqueous TBHP (70%, 0.310 mL, 3.20 mmol). Purification by FCC afforded the product a yellow solid (105 mg, 0.410 mmol, 51%).

R_f = 0.44 (hexanes/EtOAc 10:1); Mp = 140-141 °C; IR (ATR): 3055, 2922, 2853, 1710, 1617, 1600, 1455, 1422, 1399, 1188, 1146, 1110, 942, 847, 756, 736, 698 cm^{-1}; δ_H(400 MHz, CD_2Cl_2): 7.88 (1H, d, *J* = 1.7 Hz, 1-H), 7.76 (1H, dd, *J* = 7.8, 1.8 Hz, 3-H), 7.67 – 7.61 (4H, m, 8-H and H_{arom}), 7.59 (1H, dt, *J* = 7.7, 1.0 Hz, H_{arom}), 7.53 (1H, td, *J* = 7.5, 1.2 Hz, 6-H), 7.50 – 7.44 (2H, m, 3'-H and 5'-H), 7.42 – 7.36 (1H, m, H_{arom}), 7.32 (1H, td, *J* = 7.4, 1.1 Hz, H_{arom}); δ_C(101 MHz, CD_2Cl_2): 193.9 (C-9), 144.6 (C-5), 143.6 (C-1), 142.6 (C_{arom}), 140.2 (C-1'), 135.3 (C_{arom}), 135.2 (C-6), 134.9 (C_{arom}), 133.6 (C-3), 129.4 (C_{arom}), 129.3 (C-3' and C-5'), 128.3 (C_{arom}), 127.2 (C_{arom}), 124.5 (C_{arom}), 123.0 (C-8), 121.2 (C_{arom}), 120.9 (C_{arom}); HRMS (EI): m/z $[M]^{\bullet+}$ calcd for $C_{19}H_{12}O^{\bullet+}$ 256.0888; found 256.0883. Data in accordance with literature values.[19]

2-Methoxy-1,3-dimethyl-9*H*-fluoren-9-one (**10s**)

This compound was prepared in accordance with **General procedure 3** from amine **15s** (106 mg, 0.440 mmol) and aqueous TBHP (70%, 0.244 mL, 1.76 mmol). Purification by FCC afforded the product as a yellow solid (65.0 mg, 0.273 mmol, 62%).

R_f = 0.49 (hexanes/EtOAc 10:1); Mp = 83 °C; IR (ATR): 2922, 2853, 1700, 1602, 1454, 1403, 1374, 1296, 1230, 1197, 1128, 1078, 1000, 917, 887, 862, 760, 746, 716, 701 cm^{-1}; δ_H(400 MHz, CD_2Cl_2): 7.54 (1H, dt, *J* = 7.3, 1.0 Hz, 8-H), 7.46 – 7.45 (1H, m, 6-H), 7.44 (1H, t, *J* = 1.0 Hz, 5-H), 7.26 – 7.22 (1H, m, 7-H), 7.22 (1H, d, *J* = 0.7 Hz, 4-H), 3.72 (3H, s, OCH_3), 2.53 (3H, s, 1-CH_3), 2.33 (3H, d, *J* = 0.7 Hz, 3-CH_3); δ_C(101 MHz, CD_2Cl_2): 194.3 (C-9), 158.3 (C-2), 143.6 (C-5a), 140.2 (C-4a), 137.6 (C-3), 135.0 (C-8a), 134.2 (C-6), 133.0 (C-1), 130.6 (C-9a), 128.2 (C-7), 123.5 (C-8), 120.5 (C-4), 119.6 (C-5), 60.0 (OCH_3), 16.8 (3-CH_3), 10.6 (1-CH_3); HRMS (EI): m/z $[M]^{\bullet+}$ calcd for $C_{16}H_{14}O_2^{\bullet+}$ 238.0994; found 238.0995.

2-((*tert*-Butyldimethylsilyl)oxy)-9*H*-fluoren-9-one (**10u**)

This compound was prepared in accordance with **General procedure 3** from amine **15u** (119 mg, 0.380 mmol) and aqueous TBHP (70%, 0.210 mL, 1.52 mmol). Purification by FCC afforded the product as a yellow oil (60.0 mg, 0.193 mmol, 51%).

R_f = 0.58 (hexanes/EtOAc 10:1); IR = 2954, 2929, 2886, 2857, 2360, 1715, 1602, 1486, 1472, 1454, 1269, 1238, 1190, 1133, 1076, 1005, 975, 885, 826, 780, 763, 733, 693, 661 cm^{-1}; δ_H(400 MHz, CD_2Cl_2): 7.57

(1H, dt, *J* = 7.3, 1.0 Hz, 8-H), 7.47 – 7.44 (2H, m, 5-H and 6-H), 7.41 (1H, dd, *J* = 8.0, 0.5 Hz, 4-H), 7.22 (1H, ddd, *J* = 7.3, 5.5, 3.0 Hz, 7-H), 7.09 (1H, dd, *J* = 2.3, 0.5 Hz, 1-H), 6.95 (1H, dd, *J* = 8.0, 2.3 Hz, 3-H), 1.00 (9H, s, $C(CH_3)_3$), 0.24 (6H, s, $Si(CH_3)_2$); δ_C(101 MHz, CD_2Cl_2): 193.8 (C-9), 157.4 (C-2), 145.2 (C-5a), 137.9 (C-4a), 136.4 (C-9a), 135.2 (C-6), 134.8 (C-8a), 128.3 (C-7), 126.0 (C-3), 124.4 (C-8), 121.8 (C-5), 120.1 (C-4), 116.4 (C-1), 25.8 ($C(\underline{C}H_3)_3$), 18.5 ($\underline{C}(CH_3)_3$), -4.3 ($Si(CH_3)_2$); HRMS (EI): m/z $[M]^{\bullet+}$ calcd for $C_{19}H_{22}O_2Si^{\bullet+}$ 310.1389; found 310.1383.

2-((2-(Trimethylsilyl)ethoxy)methoxy)-9*H*-fluoren-9-one (**10v**)

This compound was prepared in accordance with **General procedure 3** from amine **15v** (98.9 mg, 0.300 mmol) and aqueous TBHP (70%, 0.166 mL, 1.20 mmol). Purification by FCC afforded the product as a yellow oil (24.0 mg, 0.0735 mmol, 25%).

R_f = 0.47 (hexanes/EtOAc 10:1); IR (ATR): 2952, 2924, 2898, 1716, 1603, 1488, 1456, 1295, 1271, 1235, 1070, 1008, 988, 947, 830, 763, 733 cm^{-1}; δ_H(400 MHz, $CDCl_3$): 7.61 (1H, dt, *J* = 7.3, 1.0 Hz, 1-8), 7.45 – 7.42 (2H, m, 5-H and 6-H), 7.41 (1H, dd, *J* = 8.2, 0.5 Hz, 4-H), 7.34 (1H, dd, *J* = 2.4, 0.5 Hz, 1-H), 7.21 (1H, ddd, *J* = 7.3, 6.5, 2.0 Hz, 7-H), 7.12 (1H, dd, *J* = 8.1, 2.4 Hz, 3-H), 5.25 (2H, s, OCH_2O), 3.79 – 3.74 (2H, m, $OC\underline{H}_2CH_2$), 1.00 – 0.93 (2H, m, $OCH_2C\underline{H}_2$), 0.00 (9H, s, $Si(CH_3)_3$); δ_C(101 MHz, $CDCl_3$): 193.8 (C-9), 158.8 (C-2), 144.9 (C-5a), 137.9 (C-4a), 136.0 (C-9a), 134.9 (C-6), 134.5 (C-8a), 128.2 (C-7), 124.5 (C-8), 122.0 (C-3), 121.4 (C-4), 119.8 (C-5), 112.6 (C-1), 93.1 (OCH_2), 66.6 ($O\underline{C}H_2CH_2$), 18.2 ($OCH_2\underline{C}H_2$), -1.3 ($Si(\underline{C}H_3)_3$); HRMS (EI): m/z $[M]^{\bullet+}$ calcd for $C_{19}H_{22}O_3Si$ $326.1338^{\bullet+}$; found 326.1327.

N-(*tert*-Butoxycarbonyl)-2-bromobenzylamine (**13**)[20]

To a solution of 2-bromophenylmethanamine (3.00 g, 16.1 mmol, 1.00 equiv) in DCM (50 mL) were added di-*tert*-butyldicarbonate (4.57 g, 21.0 mmol, 1.30 equiv) and trimethylamine (6.74 mL, 48.4 mmol, 3.00 equiv). The resulting mixture was stirred at room temperature overnight. Upon completion of the reaction, the reaction mixture was washed with H_2O (3 x 20 mL), brine (3 x 20 mL) and dried over $MgSO_4$. The solvent was removed *in vacuo*. Purification by FCC afforded the product as a clear oil (4.08 g, 14.3 mmol, 89%).

R_f = 0.37 (hexanes/EtOAc 10:1); IR = 3347, 2977, 2929, 2360, 2339, 1658, 1522, 1440, 1363, 1281, 1248, 1159, 1107, 1051, 1025, 950, 873, 752 cm^{-1}; δ_H(400 MHz, CD_2Cl_2): 7.55 (1H, dd, *J* = 7.6, 1.2 Hz, 3-H), 7.37 (1H, dd, *J* = 7.7, 1.9 Hz, 6-H), 7.31 (1H, td, *J* = 7.7, 1.2 Hz, 5-H), 7.16 (1H, td, *J* = 7.7, 1.9 Hz, 4-H), 5.09 (1H, s, $CH_2N\underline{H}$), 4.35 (2H, d, *J* = 6.3 Hz, $C\underline{H}_2NH$), 1.43 (9H, s, $C(C\underline{H}_3)_3$); δ_C(101 MHz, CD_2Cl_2): 156.0 (NHCOO), 138.7 (C-1), 133.1 (C-3), 129.8 (C-6), 129.3 (C-4), 128.0 (C-5), 123.7 (C-2), 79.7 ($\underline{C}(CH_3)_3$), 45.2 ($\underline{C}H_2NH$), 28.5 ($C(\underline{C}H_3)_3$); HRMS (EI): m/z $[M – (CH_3)_3C]^{\bullet+}$ calcd for $C_8H_7{}^{79}BrNO_2{}^{\bullet+}$ 227.9660; found 227.9650.

2-(3’,4’,5’-Trimethoxyphenyl)benzonitrile (**14a**)[8]

This compound was prepared in accordance with **General procedure 4** from 2-bromobenzonitrile **12** (278 mg, 1.53 mmol), 3,4,5-trimethoxyphenylboronic acid **7** (357 mg, 1.68 mmol), $Pd(PPh_3)_4$ (88.4 mg,

0.0765 mmol) and Na_2CO_3 (486 mg, 4.59 mmol). Purification by FCC afforded the product as a white solid (396 mg, 1.47 mmol, 96%).

R_f = 0.24 (hexanes/EtOAc 4:1); Mp = 121-122 °C; IR (ATR): 2224, 1585, 1479, 1410, 1343, 1247, 1126, 1111, 993, 847, 772 cm^{-1}; δ_H(400 MHz, CD_2Cl_2): 7.77 (1H, ddd, *J* = 7.7, 1.4, 0.6 Hz, 6-H), 7.66 (1H, ddd, *J* = 7.9, 7.5, 1.4 Hz, 4-H), 7.55 (1H, ddd, *J* = 7.9, 1.2, 0.6 Hz, 3-H), 7.45 (1H, td, *J* = 7.7, 1.4 Hz, 5-H), 6.79 (2H, s, 2'-H and 6'-H), 3.90 (6H, s, 3'-OCH_3 and 5'-OCH_3), 3.84 (3H, s,4'-OCH_3); δ_C(101 MHz, CD_2Cl_2): 154.0 (C-3' and C-5'), 145.9 (C-2), 139.2 (C-4'), 134.3 (C-6), 134.2 (C-1'), 133.3 (C-4), 130.5 (C-3), 128.1 (C-5), 119.3 (CN), 111.8 (C-1), 106.9 (C-2' and C-6'), 61.1 (4'-OCH_3), 56.8 (3'-OCH_3 and 5'-OCH_3); HRMS (EI): m/z $[M]^{\bullet+}$ calcd for $C_{16}H_{15}NO_3^{\bullet+}$ 269.1052; found 269.1046.

5-Methoxy-2-phenylbenzonitrile (**14b1**)

This compound was prepared in accordance with **General procedure 4** from 2-bromo-5-methoxybenzonitrile **12** (848 mg, 4.00 mmol), phenylboronic acid **7** (732 mg, 6.00 mmol), $Pd(PPh_3)_4$ (231 mg, 0.200 mmol) and Na_2CO_3 (1.27 g, 12.0 mmol). Purification by FCC afforded the product as an off-white solid (803 mg, 3.84 mmol, 96%).

R_f = 0.31 (hexanes/EtOAc 10:1); Mp = 85 °C; IR (ATR): 2227, 1609, 1562, 1510, 1480, 1440, 1409, 1284, 1268, 1232, 1159, 1116, 1044, 1032, 1003, 913, 875, 825, 761, 726, 692 cm^{-1}; δ_H(400 MHz, CD_2Cl_2): 7.56 – 7.52 (2H, m, 2'-H and 6'-H), 7.51 – 7.44 (3H, m, 3'-H and 5'-H), 7.44 (1H, d, *J* = 8.6 Hz, 3-H), 7.26 (1H, d, *J* = 2.7 Hz, 6-H), 7.21 (1H, dd, *J* = 8.7, 2.7 Hz, 4-H), 3.87 (3H, s, OCH_3); δ_C(101 MHz, CD_2Cl_2): 159.1 (C-5), 138.5 (C-1'), 138.3 (C-2), 131.7 (C-3), 129.2 (C-2' and C-6' or C-3' and C-5'), 129.0 (C-2' and C-6' or C-3' and C-5'), 128.6 (C-4'), 119.9 (C-4), 119.0 (CN), 118.3 (C-6), 112.2 (C-1), 56.2 (OCH_3); HRMS (EI): m/z $[M]^{\bullet+}$ calcd for $C_{14}H_{11}NO^{\bullet+}$ 209.0841; found 209.0834.

2-(4'-Methoxyphenyl)benzonitrile (**14b2**)[21]

This compound was prepared in accordance with **General procedure 4** from 2-bromobenzonitrile **12** (364 mg, 2.00 mmol), 4-methoxyphenylboronic acid **7** (319 mg, 2.10 mmol), $Pd(PPh_3)_4$ (116 mg, 0.100 mmol) and Na_2CO_3 (636 mg, 6.00 mmol). Purification by FCC afforded the product as a white solid (404 mg, 1.93 mmol, 97%).

R_f = 0.36 (hexanes/EtOAc 10:1); Mp = 84-85 °C; IR (ATR): 3066, 2962, 2937, 2837, 2226, 1601, 1498, 1477, 1462, 1431, 1280, 1253, 1235, 1180, 1162, 1124, 1099, 1053, 1024, 1003, 807, 750 cm^{-1}; δ_H(400 MHz, CD_2Cl_2): 7.75 (1H, ddd, *J* = 7.6, 1.4, 0.6 Hz, 6-H), 7.64 (1H, td, *J* = 7.7, 1.4 Hz, 4-H), 7.55 – 7.48 (3H, m, 3-H and 2'-H and 6'-H), 7.42 (1H, td, *J* = 7.6, 1.2 Hz, 5-H), 7.07 – 7.00 (2H, m, 3'-H and 5'-H), 3.87 (3H, s, OCH_3); δ_C(101 MHz, CD_2Cl_2): 160.7 (C-4'), 145.7 (C-2), 134.2 (C-6), 133.3 (C-4), 131.2 (C-1'), 130.6 (C-2' and C-6'), 130.5 (C-3), 127.7 (C-5), 119.4 (CN), 114.6 (C-3' and C-5'), 111.6 (C-1), 55.9 (OCH_3); HRMS (EI): m/z $[M]^{\bullet+}$ calcd for $C_{14}H_{11}NO^{\bullet+}$ 209.0841; found 209.0835.

2-(3',5'-Dimethoxyphenyl)benzonitrile (**14c**)

This compound was prepared in accordance with **General procedure 4** from 2-bromobenzonitrile **12** (1.20 g, 6.60 mmol), 3,5-dimethoxyphenylboronic acid **7** (1.80 g, 9.90 mmol), $Pd(PPh_3)_4$ (381 mg, 0.330 mmol) and Na_2CO_3 (1.40 g, 13.2 mmol). Purification by FCC afforded the product as a white solid (187 mg, 0.88 mmol, 88%).

R_f = 0.26 (hexanes/EtOAc 10:1); Mp = 93 °C; IR (ATR): 2220, 1607, 1599, 1472, 1420, 1337, 1198, 1160, 1150, 1064, 847, 769, 695 cm^{-1}; δ_H(500 MHz, CD_2Cl_2): 7.76 (1H, ddd, *J* = 7.8, 1.4, 0.6 Hz, 6-H), 7.65 (1H, td, *J* = 7.7, 1.4 Hz, 4-H), 7.54 (1H, ddd, *J* = 7.7, 1.2, 0.5 Hz, 3-H), 7.46 (1H, td, *J* = 7.6, 1.3 Hz, 5-H), 6.68 (2H, d, *J* = 2.2 Hz, 2'-H and 6'-H), 6.56 (1H, t, *J* = 2.3 Hz, 4'-H), 3.84 (7H, s, OCH_3); δ_C(126 MHz, CD_2Cl_2): 161.5 (C-3' and C-5'), 145.8 (C-2), 140.8 (C-1'), 134.2 (C-6), 133.3 (C-4), 130.5 (C-3), 128.3 (C-5), 119.1 (CN), 111.8 (C-1), 107.5 (C-2' and C-6'), 101.0 (C-4'), 56.1 (OCH_3); HRMS (EI): m/z $[M]^{•+}$ calcd for $C_{15}H_{13}NO_2^{•+}$ 239.0946; found 239.0930.

2-(2'-Methoxyphenyl)benzonitrile (**14e**)[22]

This compound was prepared in accordance with **General procedure 4** from 2-bromobenzonitrile **12** (382 mg, 2.10 mmol), 2-methoxyphenylboronic acid **7** (319 mg, 2.10 mmol), $Pd(PPh_3)_4$ (121 mg, 0.105 mmol) and Na_2CO_3 (668 mg, 6.30 mmol). Purification by FCC afforded the product as a white solid (391 mg, 1.87 mmol, 89%).

R_f = 0.32 (hexanes/EtOAc 10:1); Mp = 77 °C; IR (ATR): 2993, 2939, 2835, 2223, 1610, 1515, 1479, 1442, 1435, 1299, 1269, 1247, 1183, 1159, 1034, 832, 819, 749 cm^{-1}; δ_H(400 MHz, CD_2Cl_2): 7.73 (1H, ddd, *J* = 7.7, 1.4, 0.7 Hz, 6-H), 7.67 – 7.62 (1H, m, 4-H), 7.48 – 7.42 (4H, m, 5-H and 4'-H and 3-H), 7.29 – 7.25 (1H, m, 6'-H), 7.11 – 7.04 (2H, m, 3'-H and 5'-H), 3.83 (3H, s, OCH_3); δ_C(101 MHz, CD_2Cl_2): 157.1 (C-2'), 143.1 (C-2), 133.2 (C-3), 132.9 (C-5), 131.5 (C-5 or C-4'), 131.4 (C-6'), 130.9 (C-4'), 128.0 (C-3 or C-4' or C-5), 128.0 (C-1'), 121.2 (C-3 or C-4' or C-5), 119.0 (CN), 113.9 (C-1), 111.8 (C-3' or C-5'), 56.0 (OCH_3); HRMS (EI): m/z $[M]^{•+}$ calcd for $C_{14}H_{11}NO^{•+}$ 209.0841; found 209.0834.

2-(3'-Methoxyphenyl)benzonitrile (**14f**)[23]

This compound was prepared in accordance with **General procedure 4** from 2-bromobenzonitrile **12** (750 mg, 4.12 mmol), 3-methoxyphenylboronic acid **7** (939 mg, 6.18 mmol), $Pd(PPh_3)_4$ (238 mg, 0.206 mmol) and Na_2CO_3 (1.31 g, 12.4 mmol). Purification by FCC afforded the product as a white solid (787 mg, 3.76 mmol, 91%).

R_f = 0.33 (hexanes/EtOAc 10:1); Mp = 63-64 °C; IR (ATR): 2938, 2835, 2218, 1603, 1590, 1564, 1470, 1438, 1420, 1308, 1251, 1219, 1188, 1166, 1040, 1019, 994, 864, 780, 760, 741, 693 cm^{-1}; δ_H(400 MHz, CD_2Cl_2): 7.77 (1H, ddd, *J* = 7.7, 1.4, 0.6 Hz, 6-H), 7.66 (1H, td, *J* = 7.7, 1.4 Hz, 4-H), 7.54 (1H, ddd, *J* = 7.9, 1.3, 0.6 Hz, 3-H), 7.47 (1H, td, *J* = 7.6, 1.3 Hz, 5-H), 7.42 (1H, dd, *J* = 8.2, 7.7 Hz, 5'-H), 7.14 (1H, ddd, *J* = 7.6, 1.7, 1.0 Hz,6-H), 7.09 (1H, dd, *J* = 2.6, 1.7 Hz. 2'-H), 7.01 (1H, ddd, *J* = 8.3, 2.6, 0.9 Hz, 4'-H), 3.86 (3H, s, OCH_3); δ_C(101 MHz, CD_2Cl_2): 160.1 (C-3'), 145.6 (C-2), 140.0 (C-1'), 134.1 (C-6), 133.2

(C-4), 130.4 (C-3), 130.1 (C-5'), 128.1 (C-5), 121.5 (C-6'), 119.0 (CN), 114.8 (C-2'), 114.5 (C-4'), 111.7 (C-1), 55.8 (OCH_3); HRMS (EI): m/z $[M]^{\bullet+}$ calcd for $C_{14}H_{11}NO^{\bullet+}$ 209.0841; found 209.0835.

2-[2'-(Trifluoromethyl)phenyl]benzonitrile (**14g**)

This compound was prepared in accordance with **General procedure 4** from 2-bromobenzonitrile **12** (370 mg, 2.03 mmol), 2-(trifluoromethyl)phenylboronic acid **7** (501 mg, 2.64 mmol), $Pd(PPh_3)_4$ (117 mg, 0.101 mmol) and Na_2CO_3 (645 mg, 6.09 mmol). Purification by FCC afforded the product as a white solid (400 mg, 1.62 mmol, 80%).

R_f = 0.30 (hexanes/EtOAc 10:1); Mp = 64 °C; IR (ATR): 2227, 1595, 1581, 1473, 1439, 1314, 1262, 1173, 1160, 1107, 1069, 1033, 768, 720 cm^{-1}; δ_H(400 MHz, CD_2Cl_2): 7.83 (1H, ddt, *J* = 7.8, 1.3, 0.7 Hz, 3'-H), 7.77 (1H, ddd, *J* = 7.7, 1.4, 0.6 Hz, 6-H), 7.71 – 7.60 (3H, m, 4-H and 4'-H and 5'-H), 7.54 (1H, td, *J* = 7.7, 1.2 Hz, 5-H), 7.43 (1H, ddt, *J* = 7.8, 1.2, 0.7 Hz, 3-H), 7.39 (1H, ddt, *J* = 7.6, 1.5, 0.7 Hz, 6'-H); δ_C(101 MHz, CD_2Cl_2): 143.3 (C-2), 137.5 (d, *J* = 1.9 Hz, C-1'), 132.8 (C-6), 132.3 (C-4), 132.2 (C-5'), 131.9 (C-6'), 130.8 (d, *J* = 1.6 Hz, C-3), 129.4 (C-4' or C-5'), 128.9 (C-4' or C-5'), 126.7 (q, *J* = 5.2 Hz, C-3'), 125.7 (C-2' or CF_3), 122.9 (C-2' or CF_3), 117.8 (CN), 113.4 (C-1); HRMS (EI): m/z $[M]^{\bullet+}$ calcd for $C_{14}H_8F_3N^{\bullet+}$ 247.0609; found 247.0602.

2-[4'-(Trifluoromethyl)phenyl]benzonitrile (**14h**)[24]

This compound was prepared in accordance with **General procedure 4** from 2-bromobenzonitrile **12** (364 mg, 2.00 mmol), 4-(trifluoromethyl)phenylboronic acid **7** (570 mg, 3.00 mmol), $Pd(PPh_3)_4$ (116 mg, 0.100 mmol) and Na_2CO_3 (636 mg, 6.00 mmol). Purification by FCC afforded the product as a white solid (465 mg, 1.88 mmol, 94%).

R_f = 0.30 (hexanes/EtOAc 10:1); Mp = 100-101 °C; IR (ATR): 2225, 1618, 1565, 1480, 1407, 1327, 1269, 1196, 1164, 1102, 1069, 1019, 1006, 842, 765, 733, 708 cm^{-1}; δ_H(400 MHz, CD_2Cl_2): 7.8 (1H, ddd, *J* = 7.8, 1.4, 0.7 Hz, 6-H), 7.8 – 7.8 (2H, m, 3'-H and 5'-H), 7.7 – 7.7 (3H, m, 4-H and 2'-H and 6'-H), 7.6 – 7.5 (2H, m, 5-H and 3-H); δ_C(101 MHz, CD_2Cl_2): 143.8 (C-2), 141.9 (C-1'), 133.8 (C-5), 133.1 (C-3), 130.5 (d, *J* = 32.6 Hz, C-4'), 130.1 (C-3), 129.3 (C-2' and C-6'), 128.4 (C-5), 125.6 (q, *J* = 3.9 Hz, C-3' and C-5'), 122.8 (CF_3), 118.2 (CN), 111.3 (C-1); HRMS (EI): m/z $[M]^{\bullet+}$ calcd for $C_{14}H_8F_3N^{\bullet+}$ 247.0609; found 247.0602.

2-[3'-(Trifluoromethyl)phenyl]benzonitrile (**14i**)[8]

This compound was prepared in accordance with **General procedure 4** from 2-bromobenzonitrile **12** (382 mg, 2.10 mmol), 3-(trifluoromethyl)phenylboronic acid **7** (598 mg, 3.15 mmol), $Pd(PPh_3)_4$ (121 mg, 0.105 mmol) and Na_2CO_3 (668 mg, 6.30 mmol). Purification by FCC afforded the product as a solid (493 mg, 1.99 mmol, 95%).

R_f = 0.32 (hexanes/EtOAc 10:1); Mp = 56-57 °C; IR (ATR): 2961, 2936, 1581, 1508, 1483, 1449, 1408, 1343, 1233, 1171, 1117, 1002, 954, 830, 766, 732 cm^{-1}; δ_H(400 MHz, CD_2Cl_2): 7.84 – 7.79 (3H, m, C_{arom}), 7.77 – 7.73 (1H, m, C_{arom}), 7.70 (1H, dd, *J* = 7.7, 1.4 Hz, C_{arom}), 7.69 – 7.64 (1H, m, C_{arom}), 7.57 – 7.50

(2H, m, C_{arom}); δ_C(101 MHz, CD_2Cl_2): 144.1 (C_{arom}), 139.5 (C_{arom}), 134.2 (C_{arom}), 133.5 (C_{arom}), 132.8 (C_{arom}), 131.5 (C_{arom}), 131.1 (C_{arom}), 130.5 (C_{arom}), 129.7 (C_{arom}), 128.8 (C_{arom}), 126.1 (q, *J* = 3.9 Hz, C_{arom}), 125.8 (q, *J* = 3.9 Hz, C_{arom}), 123.1 (C_{arom}), 118.6 (C_{arom}), 111.8 (C_{arom}); HRMS (EI): m/z $[M]^{•+}$ calcd for $C_{14}H_8F_3N^{•+}$ 247.0609; found 247.0605.

2-(4'-Chlorophenyl)benzonitrile (**14j**)[25]

This compound was prepared in accordance with **General procedure 4** from 2-bromobenzonitrile **12** (482 mg, 2.65 mmol), 4-chlorophenylboronic acid **7** (622 mg, 3.97 mmol), $Pd(PPh_3)_4$ (306 mg, 0.265 mmol) and K_2CO_3 (1.31 g, 7.95 mmol). Purification by FCC afforded the product as a white solid (540 mg, 2.53 mmol, 95%).

R_f = 0.37 (hexanes/EtOAc 10:1); Mp = 114-115 °C; IR (ATR): 3058, 2923, 2852, 2224, 1593, 1497, 1473, 1440, 1397, 1281, 1090, 1017, 1005, 884, 829, 818, 764, 749, 734 cm^{-1}; ^1H NMR (400 MHz, CD_2Cl_2) δ = 7.78 (ddd, *J* = 7.8, 1.4, 0.6 Hz, 1H, 6-H), 7.68 (td, *J* = 7.7, 1.4 Hz, 1H, 4-H), 7.54 – 7.49 (m, 5H, H_{arom}), 7.49 – 7.46 (m, 1H, H_{arom}); δ_C(101 MHz, CD_2Cl_2): 144.5 (C-2), 137.2 (C-1' or C-4'), 135.2 (C-1' or C-4'), 134.1 (C-6), 133.4 (C-4), 130.6 (C-2' and C-6' or C-3' and C-5'), 130.4 (C-3 or C-5), 129.3 (C-2' and C-6' or C-3' and C-5'), 128.4 (C-3 or C-5), 111.6 (C-1); HRMS (EI): m/z $[M]^{•+}$ calcd for $C_{13}H_8{}^{35}ClN^{•+}$ 213.0345; found 213.0340.

2-(4'-Cyanophenyl)-*N*-(*tert*-butoxycarbonyl)benzylamine (**14k**)

This compound was prepared in accordance with **General procedure 4** from bromide **13** (572 mg, 2.00 mmol), 4-cyanophenylboronic acid **7** (441 mg, 3.00 mmol), $Pd(PPh_3)_4$ (231 mg, 0.200 mmol) and K_2CO_3 (991 mg, 6.00 mmol). Purification by FCC afforded the product as a white solid (415 mg, 1.35 mmol, 67%).

R_f = 0.40 (hexanes/EtOAc 4:1); Mp = 125 °C; IR (ATR): 3327, 2961, 2923, 2229, 1687, 1608, 1532, 1477, 1424, 1390, 1365, 1294, 1277, 1254, 1169, 1156, 1050, 961, 942, 885, 849, 771 cm^{-1}; δ_H(400 MHz, DMSO-*d*$_6$): 7.91 (2H, d, *J* = 8.3 Hz, 3'-H and 5'-H), 7.57 (2H, d, *J* = 8.2 Hz, 2'-H and 6'-H), 7.45 – 7.40 (2H, m, 4-H and 5-H), 7.38 – 7.31 (2H, m, 6-H and $CH_2N\underline{H}$), 7.24 – 7.19 (1H, m, 6-H), 4.05 (2H, d, *J* = 6.0 Hz, $C\underline{H}_2NH$), 1.36 (9H, s, $C(C\underline{H}_3)_3$); δ_C(101 MHz, DMSO-*d*$_6$): 155.62 (NHCOO), 145.30 (C-1'), 138.83 (C-1), 137.02 (C-2), 132.18 (C-3' and C-5'), 130.15 (C-2' and C-6'), 129.40 (C-3), 128.33 (C-5), 127.67 (C-4), 126.94 (C-6), 118.84 (CN), 110.00 (C-4'), 77.83 ($\underline{C}(CH_3)_3$), 41.16 ($\underline{C}H_2NH$), 28.20 ($C(\underline{C}H_3)_3$); HRMS (EI): m/z $[M]^{•+}$ calcd for $C_{19}H_{20}N_2O_2{}^{•+}$ 308.1525; found 308.1525.

2-(3',5'-Dimethoxyphenyl)-5-methoxybenzonitrile (**14l**)

This compound was prepared in accordance with **General procedure 4** from 2-bromo-5-methoxybenzonitrile **12** (999 mg, 4.71 mmol), 3,5-dimethoxyphenylboronic acid **7** (1.03 g, 5.65 mmol), $Pd(PPh_3)_4$ (272 mg, 0.236 mmol) and Na_2CO_3 (998 mg, 9.42 mmol). Purification by FCC afforded the product as a white solid (1.03 g, 3.82 mmol, 81%).

R_f = 0.36 (hexanes/EtOAc 4:1); Mp = 83 - 91 °C; IR (ATR): 2222, 1738, 1600, 1563, 1500, 1455, 1426, 1407, 1450, 1297, 1276, 1258, 1236, 1205, 1156, 1119, 1064, 1022, 927, 840, 806, 693 cm^{-1}; δ_H(400 MHz, CD_2Cl_2): 7.45 (1H, dd, *J* = 8.6, 0.5 Hz, 3-H), 7.25 (1H, m, 6-H), 7.19 (1H, dd, *J* = 8.7, 2.8 Hz, 4-H), 6.65 (2H, d, *J* = 2.2 Hz, 2'-H and 6'-H), 6.52 (1H, t, *J* = 2.3 Hz, 4'-H), 3.87 (3H, s, 4-OCH_3), 3.83 (6H, s, 3'-OCH_3 and 5'-OCH_3); δ_C(101 MHz, CD_2Cl_2): 160.9 (C-3' and C-5'), 158.8 (C-5), 139.9 (C-1'), 137.7 (C-2), 131.1 (C-3), 119.4 (C-4), 118.5 (C-1), 117.9 (C-6), 111.8 (CN), 106.9 (C-2' and C-6'), 100.0 (C-4'), 55.8 (4-OCH_3), 55.5 (3'-OCH_3 and 5'-OCH_3); HRMS (EI): m/z $[M]^{•+}$ calcd for $C_{16}H_{15}NO_3^{•+}$ 269.1052; found 269.1043.

2-(2'-Acetamidophenyl)-*N*-(*tert*-butoxycarbonyl)benzylamine (**14m**)

This compound was prepared in accordance with **General procedure 4** from bromide **13** (572 mg, 2.00 mmol), 2-acetamidophenylboronic acid **7** (537 mg, 3.00 mmol), $Pd(PPh_3)_4$ (231 mg, 0.200 mmol) and K_2CO_3 (991 mg, 6.00 mmol). Purification by FCC afforded the product as a white solid (467 mg, 1.37 mmol, 69%).

R_f = 0.38 (hexanes/EtOAc 2:1); Mp = 62 °C; IR (ATR): 3287, 2931, 2360, 1668, 1622, 1582, 1518, 1442, 1364, 1290, 1250, 1170, 1045, 1007, 933, 860, 755 cm^{-1}; δ_H(400 MHz, DMSO-d_6): 8.86 (1H, s, N$\underline{H}$$COCH_3$), 7.59 (1H, dd, *J* = 8.5, 6.2 Hz, H_{arom}), 7.39 – 7.33 (3H, m, $CH_2N\underline{H}$ and H_{arom}), 7.28 (1H, ddd, *J* = 7.6, 5.5, 3.1 Hz, H_{arom}), 7.25 – 7.20 (1H, m, H_{arom}), 7.16 (1H, dd, *J* = 7.6, 1.7 Hz, H_{arom}), 7.04 (1H, d, *J* = 7.5 Hz, H_{arom}), 3.95 – 3.76 (2H, m, $C\underline{H_2}NH$), 1.81 (3H, s, $NHCOC\underline{H_3}$), 1.36 (9H, s, $C(C\underline{H_3})_3$); δ_C(101 MHz, DMSO-d_6): 168.3 ($NH\underline{C}OCH_3$), 155.9 ($\underline{C}OO$), 137.7 (C_{arom}), 137.3 (C-2), 134.6 (C_{arom}), 130.3 (C_{arom}), 129.7 (C_{arom}), 127.7 (C_{arom}), 126.7 (C_{arom}), 126.4 (C_{arom}), 125.5 (C_{arom}), 125.0 (C_{arom}), 77.9 ($\underline{C}(CH_3)_3$), 41.0 (C-7), 28.2 ($C(\underline{C}H_3)_3$), 23.1 ($NHCO\underline{C}H_3$); HRMS (EI): m/z $[M]^{•+}$ calcd for $C_{20}H_{24}N_2O_3^{•+}$ 340.1787; found 340.1772.

2-(3',4'-Dimethoxyphenyl)benzonitrile (**14n**)[26]

This compound was prepared in accordance with **General procedure 4** from 2-bromobenzonitrile **12** (1.10 g, 6.05 mmol), 3,4-dimethoxyphenylboronic acid **7** (1.32 g, 7.26 mmol), $Pd(PPh_3)_4$ (350 mg, 0.302 mmol) and Na_2CO_3 (1.28 g, 12.1 mmol). Purification by FCC afforded the product as a white-yellow solid (1.43 g, 5.98 mmol, 99%).

R_f = 0.27 (hexanes/EtOAc 4:1); Mp = 141-142 °C; IR (ATR): 2219, 1601, 1521, 1481, 1463, 1439, 1333, 1263, 1246, 1217, 1145, 1021, 873, 815, 757 cm^{-1}; δ_H(400 MHz, CD_2Cl_2): 7.75 (1H, ddd, *J* = 7.7, 1.4, 0.6 Hz, 6-H), 7.64 (1H, td, *J* = 7.7, 1.4 Hz, 4-H), 7.53 (1H, ddd, *J* = 7.9, 1.2, 0.6 Hz, 3-H), 7.43 (1H, td, *J* = 7.7, 1.2 Hz, 5-H), 7.14 (1H, dd, *J* = 8.2, 2.1 Hz, 6'-H), 7.10 (1H, d, *J* = 2.2 Hz, 2'-H), 7.00 (1H, d, *J* = 8.3 Hz, 5'-H), 3.90 (6H, s, OCH_3); δ_C(101 MHz, CD_2Cl_2): 150.4 (C-4'), 149.7 (C-3'), 145.8 (C-2), 134.3 (C-6), 133.3 (C-4), 131.4 (C-1'), 130.5 (C-3), 127.7 (C-5), 121.9 (C-6'), 119.5 (CN), 112.9 (C-2'), 112.0 (C-5'), 111.6 (C-1), 56.5 (OCH_3), 56.4 (OCH_3); HRMS (EI): m/z $[M]^{•+}$ calcd for $C_{15}H_{13}NO_2^{•+}$ 239.0946; found 239.0943.

2-(4'-Methyl-3'-nitrophenyl)-*N*-(*tert*-butoxycarbonyl)benzylamine (**14o**)

This compound was prepared in accordance with **General procedure 4** from bromide **13** (572 mg, 2.00 mmol), 4-methyl-3-nitrophenylboronic acid **7** (543 mg, 3.00 mmol), $Pd(PPh_3)_4$ (231 mg, 0.200 mmol) and K_2CO_3 (991 mg, 6.00 mmol). Purification by FCC afforded the product as a white solid (416 mg, 1.21 mmol, 67%).

R_f = 0.46 (hexanes/EtOAc 4:1); Mp = 106 °C; IR (ATR): 3309, 2979, 2914, 1698, 1681, 1520, 1498, 1452, 1364, 1341, 1294, 1276, 1249, 1158, 1049, 1030, 935, 859, 836, 794, 754 cm^{-1}; δ_H(500 MHz, CD_2Cl_2): 7.92 (1H, d, *J* = 1.8 Hz, 2'-H), 7.49 (1H, dd, *J* = 7.8, 1.9 Hz, 6'-H), 7.45 (1H, dd, *J* = 7.7, 1.6 Hz, 3-H), 7.42 (1H, d, *J* = 7.8 Hz, 5'-H), 7.40 (1H, td, *J* = 7.5, 1.5 Hz, 5-H), 7.36 (1H, td, *J* = 7.4, 1.7 Hz, 4-H), 7.25 (1H, dd, *J* = 7.4, 1.5 Hz, 6-H), 4.22 (2H, d, *J* = 5.9 Hz, $C\underline{H}_2NH$), 2.63 (3H, s, CH_3), 1.39 (9H, s, $C(C\underline{H}_3)_3$); δ_C(126 MHz, CD_2Cl_2): 156.0 ($\underline{C}OO$), 149.7 (C-3'), 140.4 (C-1), 139.6 (C-1'), 137.0 (C-2), 134.2 (C-6'), 133.3 (C-5'), 132.9 (C-4'), 130.6 (C-6), 129.0 (C-4 or C-5), 128.9 (C-4 or C-5), 128.0 (C-3), 125.5 (C-2'), 79.8 ($\underline{C}(CH_3)_3$), 42.9 ($\underline{C}H_2NH$), 28.6 ($C(\underline{C}H_3)_3$), 20.4 (CH_3); HRMS (ESI): m/z $[M - H]^-$ calcd for $C_{19}H_{21}N_2O_4^-$ 341.1507; found 341.1509.

2-(Benzo[d][1,3]dioxol-5-yl)benzonitrile (**14p1**) [27]

This compound was prepared in accordance with **General procedure 4** from 2-bromobenzonitrile **12** (366 mg, 2.01 mmol), 4-methoxy-3-methylphenylboronic acid **7** (511 mg, 3.01 mmol), $Pd(PPh_3)_4$ (232 mg, 0.201 mmol) and K_2CO_3 (996 mg, 6.03 mmol). Purification by FCC afforded the product as a white solid (357 mg, 1.74 mmol, 80%).

R_f = 0.26 (hexanes/EtOAc 10:1); Mp = 96 °C; IR (ATR): 3066, 2920, 2223, 1596, 1506, 1478, 1435, 1348, 1285, 1246, 1231, 1108, 1038, 952, 928, 891, 867, 809, 756, 745 cm^{-1}; δ_H(500 MHz, CD_2Cl_2): 7.74 (1H, ddd, *J* = 7.8, 1.4, 0.6 Hz, 6-H), 7.63 (1H, td, *J* = 7.7, 1.5 Hz, 4-H), 7.48 (1H, ddd, *J* = 7.9, 1.4, 0.6 Hz, 3-H), 7.43 (1H, td, *J* = 7.6, 1.2 Hz, 5-H), 7.05 – 7.03 (1H, m, 6'-H), 7.03 (1H, s, 2'-H), 6.97 – 6.91 (1H, m, 5'-H), 6.05 (3H, s, OCH_2O); δ_C(126 MHz, CD_2Cl_2): 148.6 (C-3' or C-4'), 148.4 (C-3' or C-4'), 145.4 (C-2), 134.1 (C-6), 133.2 (C-4), 132.6 (C-1'), 130.4 (C-3), 127.8 (C-5), 123.2 (C-2'), 119.1 (CN), 111.6 (C-2), 109.5 (C-6'), 108.8 (C-5'), 102.1 (OCH_2O); HRMS (EI): m/z $[M]^{\bullet+}$ calcd for $C_{14}H_9NO_2^{\bullet+}$ 223.0633; found 223.0627.

2-(4'-Methoxy-3'-methylphenyl)benzonitrile (**14q**)

This compound was prepared in accordance with **General procedure 4** from 2-bromobenzonitrile **12** (370 mg, 2.03 mmol), 4-methoxy-3-methylphenylboronic acid **7** (521 mg, 3.04 mmol), $Pd(PPh_3)_4$ (235 mg, 0.200 mmol) and K_2CO_3 (1006 mg, 6.09 mmol). Purification by FCC afforded the product as a white solid (389 mg, 1.74 mmol, 85%).

R_f = 0.39 (hexanes/EtOAc 10:1); Mp = 87 °C; IR (ATR): 2220, 1611, 1597, 1512, 1478, 1440, 1304, 1273, 1248, 1140, 1109, 1025, 806, 760 cm^{-1}; δ_H(400 MHz, CD_2Cl_2): 7.74 (1H, ddd, *J* = 7.8, 1.4, 0.6 Hz, 6-H), 7.63 (1H, td, *J* = 7.7, 1.4 Hz, 4-H), 7.50 (1H, ddd, *J* = 7.9, 1.3, 0.6 Hz, 3-H), 7.45 – 7.36 (2H, m, 5-H and 6'-H), 7.34 – 7.33 (1H, m, 2'-H), 6.96 (1H, d, *J* = 8.4 Hz, 4-H), 3.89 (3H, s, OCH_3), 2.28 (3H, d, *J* = 0.7

Hz, CH_3); ^{13}C NMR (101 MHz, CD_2Cl_2) δ = 158.8 (C-4'), 145.8 (C-2), 134.0 (C-6), 133.1 (C-4), 131.3 (C-2'), 130.6 (C-1'), 130.3 (C-3), 127.8 (C-6'), 127.4 (C-3'), 127.4 (C-5), 119.3 (CN), 111.4 (C-1), 110.3 (C-5'), 55.8 (OCH_3), 16.4 (CH_3); HRMS (EI): m/z $[M]^{\bullet+}$ calcd for $C_{15}H_{13}NO^{\bullet+}$ 223.0997; found 223.0988.

2-[4-(4-Phenyl)-phenyl]benzonitrile (**14r**) [28]

This compound was prepared in accordance with **General procedure 4** from 2-bromobenzonitrile **12** (983 mg, 5.40 mmol), 4-biphenylboronic acid **7** (1.60 g, 8.10 mmol), $Pd(PPh_3)_4$ (624 mg, 0.540 mmol) and K_2CO_3 (2.68 g, 16.2 mmol). Purification by FCC afforded the product as a white solid (1.28 g, 5.02 mmol, 93%).

R_f = 0.35 (hexanes/EtOAc 10:1); Mp = 104-105 °C; IR = 3064, 3032, 2918, 2849, 2219, 1594, 1475, 1447, 1439, 1399, 1118, 1039, 1004, 844, 759, 740, 696, 680 cm^{-1}; δ_H(400 MHz, CD_2Cl_2): 7.80 (1H, ddd, J = 7.7, 1.4, 0.6 Hz, 6-H), 7.78 – 7.73 (2H, m, 3'-H and 5'-H), 7.72 – 7.65 (5H, m, 4-H and 2'-H and 6'-H and 2''-H and 6''-H), 7.59 (1H, ddd, J = 7.8, 1.3, 0.6 Hz, 3-H), 7.54 – 7.44 (3H, m, 5-H and 3''-H and 5''-H), 7.44 – 7.35 (1H, m, 4''-H); δ_C(101 MHz, CD_2Cl_2): 145.3 (C-2), 141.8 (C-4'), 140.6 (C-1''), 137.7 (C-1'), 134.2 (C-6), 133.3 (C-4), 130.5 (C-3), 129.7 (C-3'' and C-5''), 129.3 (C-2' and C-6' or C-2'' and C-6''), 128.1 (d, J = 1.9 Hz, C-5 and C-4'), 127.7 (C-2' and C-6' or C-2'' and C-6''), 127.5 (C-3' and C-5'), 119.1 (CN), 111.6 (C-1); HRMS (EI): m/z $[M]^{\bullet+}$ calcd for $C_{19}H_{13}N^{\bullet+}$ 255.1048; found 255.1042

2-(4'-Methoxy-3',5'-dimethylphenyl)benzonitrile (**14s**)

This compound was prepared in accordance with **General procedure 4** from 2-bromobenzonitrile **12** (377 mg, 2.05 mmol), 3,5-dimethyl-4-methoxyphenylboronic acid **7** (559 mg, 3.07 mmol), $Pd(PPh_3)_4$ (239 mg, 0.205 mmol) and K_2CO_3 (1026 mg, 6.15 mmol). Purification by FCC afforded the product as a white solid (340 mg, 1.43 mmol, 70%).

R_f = 0.40 (hexanes/EtOAc 10:1); Mp = 67 °C; IR (ATR): 3061, 2919, 2851, 2223, 1572, 1445, 1329, 1235, 1196, 1167, 1113, 999, 892, 858, 756 cm^{-1}; δ_H(400 MHz, CD_2Cl_2): 7.74 (1H, ddd, J = 7.8, 1.4, 0.6 Hz, 6-H), 7.66 – 7.60 (1H, m, 4-H), 7.48 (1H, ddd, J = 7.9, 1.3, 0.6 Hz, 3-H), 7.42 (1H, td, J = 7.6, 1.3 Hz, 5-H), 7.21 (2H, p, J = 0.6 Hz. 2'-H and 6'-H), 3.78 (3H, s, OCH_3), 2.35 (6H, t, J = 0.7 Hz, CH_3); δ_C(101 MHz, CD_2Cl_2): 158.0 (C-4'), 145.7 (C-2), 134.1 (C-1'), 134.0 (C-6), 133.1 (C-4), 131.7 (C-3' and C-5'), 130.4 (C-3), 129.6 (C-2' and C-6'), 127.6 (C-5), 119.2 (CN), 111.5 (C-1), 60.1 (OCH_3), 16.3 (CH_3); HRMS (EI): m/z $[M]^{\bullet+}$ calcd for $C_{16}H_{15}NO^{\bullet+}$ 237.1154; found 237.1147.

2-(4'-Hydroxyphenyl)benzonitrile (**14t**) [29]

This compound was prepared in accordance with **General procedure 4** from 2-bromobenzonitrile **12** (1.27 g, 6.97 mmol), 4-hydroxyphenylboronic acid **7** (1.32 g, 9.55 mmol), $Pd(PPh_3)_4$ (403 mg, 0.349 mmol) and K_2CO_3 (3.46 g, 20.9 mmol). Purification by FCC afforded the product as a white solid (982 mg, 5.03 mmol, 72%).

R_f = 0.32 (hexanes/EtOAc 4:1); Mp = 179-180 °C; IR (ATR): 3355, 2234, 1613, 1590, 1519, 1478, 1444, 1359, 1279, 1220, 1173, 1101, 825, 754 cm^{-1}; δ_H(400 MHz, MeOD): 7.77 (1H, ddd, J = 7.8, 1.4, 0.6 Hz,

6-H), 7.68 (1H, td, J = 7.6, 1.4 Hz, 4-H), 7.53 (1H, ddd, J = 7.7, 1.4, 0.6 Hz, 3-H), 7.45 (1H, td, J = 7.8, 1.2 Hz, 5-H), 7.42 – 7.38 (2H, m, 2'-H and 6'-H), 6.94 – 6.86 (2H, m, 3'-H and 5'-H); ^{13}C NMR (101 MHz, MeOD) δ = 159.4 (C-4'), 146.9 (C-2), 134.8 (C-6), 134.2 (C-4), 131.1 (C-2' and C-6'), 131.1 (C-3), 130.8 (C-1'), 128.2 (C-5), 119.9 (CN), 116.5 (C-3' and C-5'), 111.8 (C-1); HRMS (EI): m/z $[M]^{\bullet+}$ calcd for $C_{13}H_9NO^{\bullet+}$ 195.0684; found 195.0678.

2-(4'-((*tert*-Butyldimethylsilyl)oxy)phenyl)benzonitrile (**14u**)

This compound was prepared in accordance with **General procedure 6** from phenol **14t** (508 mg, 2.60 mmol), imidazole (447 mg, 6.50 mmol) and TBS-Cl (470 mg, 3.12 mmol). Purification by FCC afforded the product as a white solid (708 mg, 2.29 mmol, 88%).

R_f = 0.51 (hexanes/EtOAc 10:1); Mp = 84 °C; IR (ATR): 2954, 2854, 2224, 1604, 1510, 1475, 1250, 1171, 1100, 1005, 900, 850, 781, 764 cm^{-1}; δ_H(400 MHz, CD_2Cl_2): 7.75 (1H, ddd, J = 7.8, 1.4, 0.6 Hz, 6-H), 7.64 (1H, td, J = 7.7, 1.4 Hz, 4-H), 7.54 – 7.47 (1H, m, 3-H), 7.48 – 7.44 (2H, m, 2'-H and 6'-H), 7.42 (1H, td, J = 7.6, 1.3 Hz, 5-H), 7.01 – 6.94 (2H, m, 3'-H and 5'-H), 1.02 (9H, s, $C(CH_3)_3$), 0.26 (6H, s, $Si(CH_3)_2$); ^{13}C NMR (101 MHz, CD_2Cl_2) δ = 156.8 (C-4'), 145.5 (C-22), 134.1 (C-6), 133.2 (C-4), 131.7 (C-1'), 130.4 (C-2' and C-6'), 130.3 (C-3), 127.5 (C-5), 120.6 (C-3' and C-5'), 119.2 (CN), 111.4 (C-1), 25.8 ($C(\underline{C}H_3)_3$), 18.5 ($\underline{C}(CH_3)_3$) -4.3 ($Si(\underline{C}H_3)_2$); HRMS (EI): m/z $[M]^{\bullet+}$ calcd for $C_{19}H_{23}NOSi^{\bullet+}$ 309.1549; found 309.1542.

2-(4'-((2-(Trimethylsilyl)ethoxy)methoxy)phenyl)benzonitrile (**14v**)

Phenol **14t** (264 mg, 1.35 mmol, 1.00 equiv) was dissolved in dry THF (10 mL) and DIPEA (1.17 mL, 6.75 mmol, 5.00 equiv) was added, followed by the slow addition of the SEM-Cl (0.717 mL, 4.05 mmol, 3.00 equiv). The mixture was stirred at room temperature overnight and then poured into a H_2O-ice mixture (20 mL) and extracted with diethyl ether (3 x 10 mL). Purification by FCC afforded the product as a white solid (249 mg, 0.765 mmol, 57%).

R_f = 0.43 (hexanes/EtOAc 10:1); Mp = 81 °C; IR (ATR): 2950, 2897, 2224, 1607, 1514, 1477, 1443, 1223, 1178, 1095, 1021, 988, 942, 928, 859, 833, 763, 691 cm^{-1}; δ_H(400 MHz, CD_2Cl_2): 7.75 (1H, ddd, J = 7.8, 1.4, 0.6 Hz, 6-H), 7.64 (1H, td, J = 7.7, 1.4 Hz, 4-H), 7.54 – 7.48 (4H, m, 3-H and 2'-H and 6'-H), 7.43 (1H, td, J = 7.6, 1.2 Hz, 5-H), 7.20 – 7.13 (2H, m, 3'-H and 5'-H), 5.28 (2H, s, $OC\underline{H}_2O$), 3.84 – 3.74 (2H, m, $OC\underline{H}_2CH_2$), 1.03 – 0.93 (2H, m, $OCH_2C\underline{H}_2$), 0.02 (9H, s, $Si(C\underline{H}_3)_3$) ; δ_C(101 MHz, CD_2Cl_2): 158.4 (C-4'), 145.4 (C-2), 134.1 (C-6), 133.2 (C-4), 132.0 (C-1'), 130.4 (C-2' and C-6'), 130.3 (C-3), 127.6 (C-5), 119.2 (CN), 116.7 (C-3' and C-5'), 111.5 (C-1), 93.3 (OCH_2O), 66.8 ($O\underline{C}H_2CH_2$), 18.4 (OCH_2CH_2), -1.4 ($Si(CH_3)_3$) ; HRMS (EI): m/z $[M]^{\bullet+}$ calcd for $C_{19}H_{23}NO_2Si^{\bullet+}$ 325.1498; found 325.1506.

2-(4'-Phenylmethoxyphenyl)benzonitrile (**14w**)

Phenol **14t** (303 mg, 1.55 mmol, 1.00 equiv) was dissolved in DMF (10 mL). K_2CO_3 (256 mg, 1.55 mmol, 1.00 equiv) and benzyl bromide (298 mg, 1.71 mmol, 1.10 equiv) were added. The suspension was warmed to 50 °C and stirred for 16 h. After completion of the reaction, H_2O was added and the mixture was extracted

with EtOAc (3 x 10 mL), washed with brine (3 x 10 mL), dried over $MgSO_4$ and concentrated. Purification by FCC afforded the product as a white solid (423 mg, 1.48 mmol, 96%)

R_f = 0.26 (hexanes/EtOAc 10:1); Mp = 100 °C; IR (ATR): 3398, 2961, 2910, 2873, 2219, 1716, 1606, 1514, 1467, 1379, 1289, 1238, 1175, 1023, 1011, 999, 828, 764, 745, 700 cm^{-1}; δ_H(400 MHz, CD_2Cl_2): 7.75 (1H, ddd, *J* = 7.8, 1.4, 0.6 Hz, 6-H), 7.64 (1H, td, *J* = 7.7, 1.4 Hz, 4-H), 7.55 – 7.50 (3H, m, 2'-H and 6'-H and H_{arom}), 7.50 – 7.46 (2H, m, H_{arom}), 7.45 – 7.39 (3H, m, 3''-H and 5''-H and H_{arom}), 7.38 – 7.33 (1H, m, H_{arom}), 7.15 – 7.08 (2H, m, 3'-H and 5'-H), 5.14 (2H, s, OCH_2); ^{13}C NMR (101 MHz, CD_2Cl_2) δ = 159.7 (C-5), 145.4 (C-2), 137.3 (C-1'), 134.1 (C-6), 133.2 (C-4), 131.3 (C-1'), 130.5 (C-2' and C-6'), 130.3 (C_{arom}), 129.0 (C-3'' and C-5''), 128.4 (C_{arom}), 128.0 (C-2'' and C-6''), 127.6 (C_{arom}), 119.3 (C_{arom}), 115.3 (C-3' and C-5'), 111.4 (C_{arom}), 70.5 (OCH_2); HRMS (EI): m/z $[M]^{•+}$ calcd for $C_{20}H_{15}NO^{•+}$ 285.1154; found 285.1151.

2-(3',4',5'-Trimethoxyphenyl)benzylamine (**15a**)

This compound was prepared in accordance with **General procedure 5** from nitrile **14a** (474 mg, 1.76 mmol), LAH (134 mg, 3.52 mmol) and $AlCl_3$ (469 mg, 3.52 mmol). Purification by FCC afforded the product as a yellow oil (327 mg, 1.20 mmol, 68%).

R_f = 0.19 (DCM/MeOH 1% + NEt_3 1%); IR (ATR): 2962, 2936, 2827, 1581, 1506, 1451, 1407, 1342, 1183, 1155, 1118, 1002, 831, 766, 670 cm^{-1}; δ_H(400 MHz, CD_2Cl_2): 7.48 (1H, ddd, *J* = 7.4, 1.6, 0.6 Hz,6-H), 7.34 (1H, td, *J* = 7.4, 1.8 Hz, 5-H), 7.30 (1H, td, *J* = 7.3, 1.6 Hz, 4-H), 7.27 – 7.23 (1H, m, 3-H), 6.59 (2H, s, 2'-H and 6'-H), 3.83 (6H, s,3'-OCH_3 and 5'-OCH_3), 3.82 (3H, s, 4'-OCH_3), 3.82 (2H, s, C$\underline{H}_2$$NH_2$); δ_C(101 MHz, CD_2Cl_2): 153.6 (C-3' and C-5'), 142.1 (C-2), 140.2 (C-1'), 137.7 (C-4'), 137.2 (C-1), 130.4 (C-3), 128.8 (C-6), 128.2 (C-5), 127.4 (C-4), 107.0 (C-2' and C-6'), 61.0 (4'-OCH_3), 56.6 (3'-OCH_3 and 5'-OCH_3), 44.3 (CH_2NH_2); HRMS (EI): $[M]^{•+}$ calcd for $C_{16}H_{19}NO_3^{•+}$ 273.1365; found 273.1360.

5-Methoxy-2-phenylbenzylamine (**15b1**)

This compound was prepared in accordance with **General procedure 1** from nitrile **14b1** (879 mg, 4.20 mmol), LAH (638 mg, 16.8 mmol) and $AlCl_3$ (2.24 g, 16.8 mmol). Purification by FCC afforded the product as a yellow oil (603 mg, 2.83 mmol, 67%).

R_f = 0.14 (DCM/MeOH 1% + NEt_3 1%); IR (ATR): 3368, 3026, 2934, 2834, 1606, 1567, 1506, 1480, 1464, 1442, 1419, 1273, 1228, 1162, 1047, 1006, 851, 815, 766, 700 cm^{-1}; δH(400 MHz, CD_2Cl_2): 7.44 – 7.37 (2H, m, 3'-H and 5'-H), 7.33 (3H, tt, *J* = 7.9, 1.4 Hz, 2'-H and 6'-H and 4'-H), 7.15 (1H, d, *J* = 8.3 Hz, 3-H), 7.04 (1H, d, *J* = 2.7 Hz, 6-H), 6.83 (1H, dd, *J* = 8.3, 2.7 Hz, 4-H), 3.84 (3H, s,OCH_3), 3.74 (2H, s, C$\underline{H}_2$$NH_2$); δ_C(101 MHz, CD_2Cl_2): 159.6 (C-5), 142.8 (C-1), 141.5 (C-1'), 134.1 (C-2), 131.5 (C-3), 129.7 (C-2' and C-4'), 128.5 (C-3' and C-5'), 127.1 (C-6'), 113.8 (C-6), 112.2 (C-4), 55.7 (OCH_3), 44.6 (CH_2NH_2); HRMS (EI): m/z $[M]^{•+}$ calcd for $C_{14}H_{15}NO^{•+}$ 213.1154; found 213.1149.

2-(4’-Methoxyphenyl)benzylamine (**15b2**)

This compound was prepared in accordance with **General procedure 5** from nitrile **14b2** (349 mg, 1.67 mmol), LAH (190 mg, 5.00 mmol) and $AlCl_3$ (312 mg, 2.34 mmol). Purification by FCC afforded the product as a yellow solid (192 mg, 0.774 mmol, 46%).

R_f = 0.15 (DCM/MeOH 1% + NEt_3 1%); Mp = 40 – 44 °C; IR (ATR): 3359, 3273, 2934, 2835, 1954, 1579, 1496, 1478, 1446, 1432. 1251, 1230. 1182, 1159, 1120, 1050, 1021, 1003, 935, 906, 853, 806, 750, 742 cm^{-1}; δ_H(400 MHz, CD_2Cl_2): 7.49 – 7.44 (1H, m, 6-H), 7.32 (1H, td, *J* = 7.4, 1.7 Hz, 5-H), 7.30 – 7.25 (3H, m, 4-H and 2’-H and 6’-H), 7.23 – 7.19 (1H, m, 3-H), 6.98 – 6.93 (2H, m, 3’-H and 5’-H), 3.84 (3H, s, OCH_3), 3.77 (2H, s, C$\underline{H}_2$$NH_2$); δ_C(101 MHz, CD_2Cl_2): 159.25 (C-4’), 141.36 (C-1), 141.25 (C-2), 133.93 (C-1’), 130.58 (C-2’) and (C-6’), 130.54 (C-3), 128.48 (C-6), 127.72 (C-5), 126.99 (C-4), 113.97 (C-3’ and C-5’), 55.65 (OCH_3), 44.45 (CH_2NH_2); HRMS (EI): m/z $[M]^{\bullet+}$ calcd for $C_{14}H_{15}NO^{\bullet+}$ 213.1154; found 213.1150.

2-(3’,5’-Dimethoxyphenyl)benzylamine (**15c**)

This compound was prepared in accordance with **General procedure 5** from nitrile **14c** (502 mg, 2.10 mmol), LAH (159 mg, 4.20 mmol) and $AlCl_3$ (560 mg, 4.20 mmol). Purification by FCC afforded the product as a yellow oil (244 mg, 1.00 mmol, 48%).

R_f = 0.17 (DCM/MeOH 1% + NEt_3 1%); IR (ATR): 2936, 2906, 2837, 2645, 1586, 1454, 1417, 1322, 1199, 1148, 1061, 1026, 927, 830, 768, 700 cm^{-1}; δ_H(400 MHz, CD_2Cl_2): 7.45 (1H, ddd, *J* = 7.4, 1.5, 0.6 Hz, 6-H), 7.37 – 7.31 (1H, m, 5-H), 7.27 (1H, td, *J* = 7.4, 1.5 Hz, 4-H), 7.24 – 7.19 (1H, m, 3-H), 6.50 (2H, d, *J* = 2.3 Hz, 2’-H and 6’-H), 6.46 (1H, t, *J* = 2.3 Hz, 4’-H), 3.80 (6H, s, OCH_3), 3.78 (2H, s, C$\underline{H}_2$$NH_2$); δ_C(101 MHz, CD_2Cl_2): 161.2 (C-3’ and C-5’), 143.9 (C-1’), 141.8 (C-2), 141.5 (C-1), 130.2 (C-3), 128.6 (C-6), 128.2 (C-5), 127.0 (C-4), 107.8 (C-2’ and C-6’), 99.5 (C-4’), 55.9 (OCH_3), 44.7 (CH_2NH_2); HRMS (EI): $[M]^{\bullet+}$ calcd for $C_{15}H_{17}NO_2^{\bullet+}$ 243.1259; found 243.1267.

2-(2’-Methoxyphenyl)benzylamine (**15e**)

This compound was prepared in accordance with **General procedure 5** from nitrile **14e** (330 mg, 1.58 mmol), LAH (179 mg, 4.73 mmol) and $AlCl_3$ (294 mg, 2.21 mmol). Purification by FCC afforded the product as a yellow solid (185 mg, 0.867 mmol, 55%).

R_f = 0.14 (DCM/MeOH 1% + NEt_3 1%); Mp = 105 °C; IR (ATR): 3360, 3273, 3058, 2961, 2934, 2835, 1711, 1661, 1610, 1514, 1497, 1479, 1433, 1363, 1294, 1233, 1179, 1120, 1021, 1002, 906, 834, 757 cm^{-1}; δ_H(400 MHz, CD_2Cl_2): 7.47 (1H, ddd, *J* = 7.5, 1.4, 0.7 Hz, 6-H), 7.40 – 7.32 (2H, m, 5-H and 4’-H), 7.27 (1H, td, *J* = 7.5, 1.5 Hz, 4-H), 7.18 – 7.10 (2H, m, 3-H and 6’-H), 7.03 (1H, td, *J* = 7.4, 1.1 Hz, 5’-H), 7.01 – 6.98 (1H, m, 3’-H), 3.76 (3H, s, OCH_3), 3.59 (2H, d, *J* = 9.6 Hz, C$\underline{H}_2$$NH_2$), 1.46 (2H, s, CH_2N$\underline{H}_2$); δ_C(101 MHz, CD_2Cl_2): 156.9 (C-2’), 142.7 (C-1), 138.1 (C-2), 131.4 (C-3 or C-6’), 130.7 (C-3 or C-6’), 130.4 (C-1’), 129.2 (C-5 and C-4’), 128.0 (C-5 and C-4’), 127.7 (C-6), 126.8 (C-4) , 120.9 (C-5’), 111.1 (C-3’), 55.7 (OCH_3), 44.6 (CH_2NH_2); HRMS (EI): m/z $[M]^{\bullet+}$ calcd for $C_{14}H_{15}NO^{\bullet+}$ 213.1154; found 213.1147.

2-(3'-Methoxyphenyl)benzylamine (**15f**)

This compound was prepared in accordance with **General procedure 5** from nitrile **14f** (738 mg, 3.52 mmol), LAH (495 mg, 13.0 mmol) and $AlCl_3$ (658 mg, 4.93 mmol). Purification by FCC afforded the product as a yellow oil (481 mg, 2.26 mmol, 64%).

R_f = 0.15 (DCM/MeOH 1% + NEt_3 1%); IR = 3058, 3001, 2936, 2834, 1596, 1579, 1474, 1421, 1316, 1293, 1210, 1176, 1043, 1019, 860, 784, 756, 702 cm^{-1}; δ_H(400 MHz, DMSO-*d*$_6$): 7.6 – 7.5 (1H, m, 6-H), 7.3 (2H, dddd, *J* = 8.3, 6.2, 3.2, 1.1 Hz, 5-H and 5'-H), 7.3 (1H, td, *J* = 7.4, 1.5 Hz, 4-H), 7.2 (1H, dd, *J* = 7.6, 1.5 Hz, 3-H), 7.0 – 6.9 (3H, m, 2'-H and 4'-H and 6'-H), 3.8 (3H, s, OCH_3), 3.6 (2H, s, $C\underline{H}_2NH_2$); δ_C(101 MHz, DMSO-*d*$_6$): 159.0 (C-4), 142.3 (C-1), 141.0 (C-1'), 140.4 (C-2), 129.3 (C-3 or C-5 or C-5'), 129.2 (C-3 or C-5 or C-5'), 128.2 (C-6 or C-5 or C-5'), 127.4 (C-5 or C-5'), 126.2 (C-4), 121.3 (C-6' or C-4'), 114.6 (C-2'), 112.6 (C-6' or C-4'), 55.1 (OCH_3), 43.2 (CH_2NH_2); HRMS (EI): m/z $[M]^{\bullet+}$ calcd for $C_{14}H_{15}NO^{\bullet+}$ 213.1154; found 213.1158.

2-[2'-(Trifluoromethyl)phenyl]benzylamine (**15g**)

This compound was prepared in accordance with **General procedure 5** from nitrile **14g** (326 mg, 1.32 mmol), LAH (225 mg, 5.94 mmol) and $AlCl_3$ (246 mg, 1.85 mmol). Purification by FCC afforded the product as a yellow oil (205 mg, 0.816 mmol, 62%).

R_f = 0.18 (DCM/MeOH 1% + NEt_3 1%); IR (ATR): 3342, 3249, 3057, 2925, 2870, 1645, 1601, 1575, 1493, 1429, 1367, 1310, 1259, 1158, 1106, 1068, 1032, 1006, 965, 758, 715 cm^{-1}; δ_H(400 MHz, DMSO-*d*$_6$): 7.83 (1H, dd, *J* = 7.9, 1.3 Hz, 3'-H), 7.70 (1H, td, *J* = 7.5, 1.4 Hz, 5'-H), 7.66 – 7.56 (2H, m, 6-H and 4'-H), 7.44 – 7.33 (2H, m, 5-H and 6'-H), 7.25 (1H, td, *J* = 7.5, 1.3 Hz, 4-H), 7.07 (1H, d, *J* = 7.5 Hz. 3-H), 3.34 (2H, s, $C\underline{H}_2NH_2$); δ_C(101 MHz, DMSO-*d*$_6$): 141.4 (C-1), 139.3 (d, *J* = 3.1 Hz, C-1'), 137.1 (C-2), 132.1 (C-6), 131.9 (C_{arom}), 128.9 (C_{arom}), 128.0 (d, *J* = 2.5 Hz, C-5 and C-6'), 127.3 (C-CF_3 or C-2'), 127.0 (C_{arom}), 127.0 (C_{arom}), 125.9 (q, *J* = 5.2 Hz, C-3') 125.4 (CF_3 or C-2'), 125.3 (C-4), 122.7 (C_{arom}), 43.1 (CH_2NH_2); HRMS (ESI): m/z $[M - H]^-$ calcd for $C_{14}H_{11}F_3N^-$ 250.0849; found 250.0839.

2-[4'-(Trifluoromethyl)phenyl]benzylamine (**15h**)

This compound was prepared in accordance with **General procedure 5** from nitrile **14h** (425 mg, 1.72 mmol), LAH (248 mg, 6.54 mmol) and $AlCl_3$ (321 mg, 2.41 mmol). Purification by FCC afforded the product as a yellow oil (271 mg, 1.08 mmol, 63%).

R_f = 0.23 (DCM/MeOH 1% + NEt_3 1%); IR (ATR): 3339, 3064, 3026, 2860, 1617, 1591, 1468, 1404, 1371, 1321, 1161, 1117, 1104, 1068, 1021, 1006, 842, 765, 756, 745, 711, 657 cm^{-1}; δ_H(400 MHz, $CDCl_3$): 7.71 – 7.66 (2H, m, 3'-H and 5'-H), 7.49 (3H, tdd, *J* = 7.5, 1.8, 0.9 Hz, 3-H and 2'-H and 6'-H), 7.41 (1H, td, *J* = 7.5, 1.5 Hz, 4-H), 7.33 (1H, td, *J* = 7.5, 1.5 Hz, 5-H), 7.22 (1H, dd, *J* = 7.6, 1.5 Hz, 6-H), 3.79 (2H, s, $C\underline{H}_2NH_2$); δ_C(101 MHz, $CDCl_3$): 145.0 (C-1'), 140.5 (C-2), 140.0 (C-1), 130.0 (C-6), 129.6 (C-2' and C-6'), 129.4 (d, *J* = 32.3 Hz, CF_3), 128.6 (C-3) , 128.4 (C-4), 127.1 (C-5), 125.4 (q, *J* = 3.7 Hz, C-3' and C-5'), 123.0 (C-4'), 44.1 (CH_2NH_2); HRMS (ESI): m/z $[M - H]^-$ calcd for $C_{14}H_{11}F_3N^-$ 250.0849; found 250.0837.

2-[3’-(Trifluoromethyl)phenyl]benzylamine (**15i**)

This compound was prepared in accordance with **General procedure 5** from nitrile **14i** (445 mg, 1.80 mmol), LAH (205 mg, 5.40 mmol) and $AlCl_3$ (336 mg, 2.52 mmol). Purification by FCC afforded the product as a yellow oil (302 mg, 1.20 mmol, 67%).

R_f = 0.21 (DCM/MeOH 1% + NEt_3 1%); IR (ATR): 3376, 3292, 3063, 2927, 2870, 1660, 1611, 1592, 1479, 1427, 1331, 1254, 1162, 1118, 1093, 1072, 1024, 905, 805, 757, 705, 657 cm^{-1}; δ_H(400 MHz, CD_2Cl_2): 7.69 – 7.62 (2H, m, 4’-H and 2’-H), 7.62 – 7.54 (2H, m, 6’-H and 5’-H), 7.54 – 7.50 (1H, m, 6-H), 7.40 (1H, td, *J* = 7.5, 1.5 Hz, 5-H), 7.33 (1H, td, *J* = 7.5, 1.5 Hz, 4-H), 7.24 (1H, dd, *J* = 7.5, 1.5 Hz, 3-H), 3.75 (2H, s, $C\underline{H_2}NH_2$); δ_C(101 MHz, CD_2Cl_2): 142.5 (C-1), 141.0 (C-1’), 140.2 (C-2), 133.1 (C-6’ or C-5’), 130.9 (q, *J* = 32.4 Hz, C-3’), 130.3 (C-3), 129.1 (C-5), 128.7 (C-6’ or C-5’), 128.7 (C-6), 127.2 (C-4), 126.4 (q, *J* = 3.8 Hz, C-2’ or C-4’), 124.2 (d, *J* = 3.9 Hz, C-2’ or C-4’), 44.2 (CH_2NH_2); HRMS (ESI): m/z $[M - H]^-$ calcd for $C_{14}H_{11}F_3N^-$ 250.0849; found 250.0831

2-(4’-Chlorophenyl)benzylamine (**15j**)

This compound was prepared in accordance with **General procedure 5** from nitrile **14j** (485 mg, 2.27 mmol), LAH (172 mg, 4.54 mmol) and $AlCl_3$ (605 mg, 4.54 mmol). Purification by FCC afforded the product as a yellow oil (193 mg, 0.887 mmol, 39%).

R_f = 0.23 (DCM/MeOH 1% + NEt_3 1%); IR (ATR): 3370, 3310, 3059, 3024, 2922, 2855, 1591, 1496, 1474, 1445, 1395, 1379, 1088, 1018, 1004, 830, 757, 736 cm^{-1}; δ_H(500 MHz, MeOD): 7.53 – 7.47 (1H, m, 6-H), 7.44 (2H, d, *J* = 8.4 Hz, 2’-H and 6’-H), 7.38 (1H, td, *J* = 7.6, 1.5 Hz, 5-H), 7.35 – 7.27 (3H, m, 4-H and 3’-H and 5’-H), 7.20 (1H, dd, *J* = 7.5, 1.5 Hz, 3-H), 3.72 (2H, s, $C\underline{H_2}NH_2$); δ_C(126 MHz, MeOD): 141.4 (C-2), 141.2 (C-1’), 140.7 (C-1), 134.3 (C-4’), 131.8 (C-3’ and C-5’), 130.9 (C-3), 129.5 (C-2’ and C-6’), 129.3 (C-6), 129.2 (C-5), 128.1 (C-4), 43.9 (CH_2NH_2); HRMS (EI): m/z $[M - H]^-$ calcd for $C_{13}H_{12}{}^{35}ClN^{\bullet+}$ 216.0586; found 216.0573.

2-(4’-Cyanophenyl)benzylamine (**15k**)

This compound was prepared in accordance with **General procedure 7** from carbamate **14k** (376 mg, 1.22 mmol) and TFA (2.99 mL, 39.0 mmol). Purification by FCC afforded the product as a yellow oil (196 mg, 0.941 mmol, 77%).

R_f = 0.14 (DCM/MeOH 1% + NEt_3 1%); IR (ATR): 3356, 2910, 2360, 2227, 1635, 1590, 1506, 1473, 1373, 1328, 1276, 1180, 1102, 1006, 882, 840, 760, 735 cm^{-1}; δ_H(400 MHz, CD_2Cl_2): 7.7 – 7.7 (2H, m, 3’-H and 5’-H), 7.6 – 7.5 (3H, m, 6-H and 2’-H and 6’-H), 7.4 (1H, td, *J* = 7.5, 1.5 Hz, 5-H), 7.3 (1H, td, *J* = 7.5, 1.4 Hz, 4-H), 7.2 (1H, dd, *J* = 7.5, 1.5 Hz, 3-H), 3.7 (2H, s, $C\underline{H_2}NH_2$); δ_C(101 MHz, CD_2Cl_2): 146.5 (C-1’), 141.0 (C-1), 139.9(C-2), 132.4 (C-3’ and C-5’), 130.4 (C-2’ and C-6’), 130.0 (C-3), 128.9 (C-5 or C-6), 128.8 (C-5 or C-6), 127.3 (C-4), 119.2 (CF_3), 111.3 (C-4’), 44.2 (CH_2NH_2); HRMS (EI): m/z $[M]^{\bullet+}$ calcd for $C_{14}H_{12}N_2^{\bullet+}$ 208.1000; found 208.0995.

2-(3’,5’-Dimethoxyphenyl)-5-methoxybenzylamine (**15l**)

This compound was prepared in accordance with **General procedure 5** from nitrile **14l** (646 mg, 2.40 mmol), LAH (319 mg, 8.40 mmol), $AlCl_3$ (1.12 g, 8.40 mmol). Purification by FCC afforded the product as a white-yellow solid (509 mg, 1.89 mmol, 79%).

R_f = 0.15 (DCM/MeOH 1% + NEt_3 1%); Mp = 60 °C; IR (ATR): 3354, 3269, 2955, 2834, 1589, 1455, 1410, 1286, 1227, 1204, 1149, 1063, 818, 702 cm^{-1}; δ_H(400 MHz, CD_2Cl_2): 7.15 (1H, d, *J* = 8.4 Hz, 3-H), 7.02 (1H, d, *J* = 2.7 Hz, 6-H), 6.81 (1H, dd, *J* = 8.4, 2.7 Hz, 4-H), 6.47 (2H, d, *J* = 2.3 Hz, 2’-H and 6’-H), 6.44 (1H, t, *J* = 2.1 Hz, 4’-H), 3.84 (3H, s, 5’-OCH_3), 3.79 (6H, s, 3’-OCH_3 and 5’-OCH_3), 3.76 (2H, s, $C\underline{H}_2NH_2$); ^{13}C NMR (101 MHz, CD_2Cl_2) δ = 161.1 (C-3’ and C-5’), 159.8 (C-5), 143.6 (C-1’), 142.9 (C-1), 134.3 (C-2), 131.3 (C-3), 114.0 (C-6), 112.3 (C-4), 108.1 (C-2’ and C-6’), 99.3 (C-4’), 55.9 (3’-OCH_3 and 5’-OCH_3), 55.8 (5’-OCH_3), 44.8 (CH_2NH_2); HRMS (EI): m/z $[M]^{\bullet+}$ calcd for $C_{16}H_{19}NO_3^{\bullet+}$ 274.1438; found 274.1439.

2-(2’-Acetamidophenyl)-benzylamine (**15m**)

This compound was prepared in accordance with **General procedure 7** from carbamate **14m** (511 mg, 1.50 mmol) and TFA (2.99 mL, 39.0 mmol). Purification by FCC afforded the product as a yellow oil (273 mg, 1.13 mmol, 76%).

R_f = 0.23 (DCM/MeOH 1% + NEt_3 1%); IR (ATR): 3275, 2966, 2361, 2341, 1670, 1581, 1540, 1521, 1474, 1439, 1368, 1301, 1007, 871, 753, 734, 700 cm^{-1}; δ_H(400 MHz, DMSO-*d*$_6$): 10.22 (1H, s, $N\underline{H}COCH_3$), 7.59 (1H, d, *J* = 8.0 Hz, 3’-H), 7.50 (1H, dd, *J* = 7.6, 1.4 Hz, 6-H), 7.36 (2H, dtd, *J* = 11.5, 7.7, 1.6 Hz, 5-H and 4’-H), 7.29 (1H, td, *J* = 7.5, 1.5 Hz, 4-H), 7.21 (1H, td, *J* = 7.4, 1.3 Hz, 5’-H), 7.14 (2H, dd, *J* = 7.6, 1.7 Hz, 6’-H), 7.06 (1H, dd, *J* = 7.5, 1.4 Hz, 3-H), 3.47 (2H, dd, *J* = 100.1, 13.1 Hz, $C\underline{H}_2NH_2$), 1.79 (3H, s, $NHCOC\underline{H}_3$); δ_C(101 MHz, DMSO-*d*$_6$): 168.1 ($NH\underline{C}OCH_3$), 140.4 (C-1), 137.9 (C-2), 136.1 (C-2’), 135.3 (C-1’), 130.5 (C-6’), 129.9 (C-3), 128.7 (C-6), 127.8 (C-5), 127.5 (C-4’), 126.6 (C-4), 125.6 (C-3’), 124.7 (C-5’), 43.3 (CH_2NH_2), 23.1 ($NHCO\underline{C}H_3$); HRMS (EI): m/z $[M]^{\bullet+}$ calcd for $C_{15}H_{16}N_2O^{\bullet+}$ 240.1263; found 240.1255.

2-(3’,4’-Dimethoxyphenyl)benzylamine (**15n**)

This compound was prepared in accordance with **General procedure 5** from nitrile **14n** (1.44 g, 6.00 mmol), LAH (683 mg, 18.0 mmol) and $AlCl_3$ (2.40 g, 18.0 mmol). Purification by FCC afforded the product as a yellow oil (793 mg, 3.26 mmol, 54%).

R_f = 0.13 (DCM/MeOH 1% + NEt_3 1%); IR (ATR): 3268, 3015, 2945, 2833, 1600, 1547, 1480, 1486, 1445, 1434, 1254, 1226, 1161, 1008, 1005, 871, 832, 734, 702 cm^{-1}; δ_H(400 MHz, CD_2Cl_2): 7.45 (1H, ddd, *J* = 7.5, 1.5, 0.6 Hz, 6-H), 7.33 (1H, td, *J* = 7.5, 1.8 Hz, 5-H), 7.28 (1H, td, *J* = 7.3, 1.5 Hz, 4-H), 7.25 – 7.21 (1H, m, 3-H), 6.94 (1H, d, *J* = 1.6 Hz, 2’-H), 6.93 (1H, d, *J* = 8.5 Hz, 5’-H), 6.89 (1H, dd, *J* = 8.2, 1.9 Hz, 6’-H), 3.87 (3H, s, 4’-OCH_3), 3.84 (3H, s,3’-OCH_3), 3.79 (2H, s, $C\underline{H}_2NH_2$); δ_C(101 MHz, CD_2Cl_2): 149.2 (C-3’), 148.8 (C-4’), 141.6 (C-1’), 141.5 (C-2), 134.3 (C-1), 130.5 (C-3), 128.5 (C-6), 127.8 (C-5), 126.9

(C-4), 121.6 (C-6’), 113.3 (C-2’), 111.6 (C-5’), 56.2 (3’-OCH_3 or 4’-OCH_3), 56.2 (3’-OCH_3 or 4’-OCH_3), 44.6 (CH_2NH_2); HRMS (ESI): m/z $[M + H]^+$ calcd for $C_{15}H_{18}NO_2^+$ 244.1332; found 244.1332.

2-(4’-Methyl-3’-nitrophenyl)benzylamine (**15o**)

This compound was prepared in accordance with **General procedure 7** from carbamate **14o** (346 mg, 1.01 mmol) and TFA (2.99 mL, 39.0 mmol). Purification by FCC afforded the product as a yellow oil (240 mg, 0.991 mmol, 98%).

R_f = 0.14 (DCM/MeOH 1% + NEt_3 1%); IR (ATR): 3377, 3060, 2930, 2865, 2360, 2341, 1558, 1523, 1499, 1443, 1342, 1202, 1158, 1079, 1033, 889, 835, 805, 753, 675 cm^{-1}; ^{1}H NMR (400 MHz, CD_2Cl_2) δ = 8.03 (d, *J* = 1.9 Hz, 1H, 3-H), 7.56 (dd, *J* = 7.8, 1.9 Hz, 1H, 6’-H), 7.54 – 7.46 (m, 1H, 6-H), 7.40 (ddd, *J* = 9.3, 7.5, 1.4 Hz, 2H, 5-H and 5’-H), 7.33 (td, *J* = 7.4, 1.5 Hz, 1H, 4-H), 7.24 (dd, *J* = 7.6, 1.5 Hz, 1H, 3-H), 3.77 (s, 2H, $C\underline{H}_2NH_2$), 2.63 (d, *J* = 0.7 Hz, 3H, CH_3); δ_C(101 MHz, CD_2Cl_2): 149.5 (C-3’), 141.2 (C-1 or C-1’), 140.7 (C-1 or C-1’), 139.2 (C-2), 134.2 (C-6’), 133.0 (C-5’), 132.5 (C-4’), 130.3 (C-3), 128.8 (C-5 or C-6), 128.8 (C-5 or C-6), 127.3 (C-4), 125.5 (C-2’), 44.3 (CH_2NH_2), 20.2 (CH_3); HRMS (EI): m/z $[M - H]^-$ calcd for $C_{14}H_{13}N_2O_2^-$ 241.0983; found 241.0973.

[2-(1,3-Benzodioxol-5-yl)phenyl]methanamine (**15p**)

This compound was prepared in accordance with **General procedure 5** from nitrile **14p** (317 mg, 1.42 mmol), LAH (216 mg, 5.68 mmol) and $AlCl_3$ (757 mg, 5.68 mmol). Purification by FCC afforded the product as a yellow oil (246 mg, 1.08 mmol, 76%).

R_f = 0.23 (DCM/MeOH 1% + NEt_3 1%); IR (ATR): 3348, 3278, 3179, 3056, 3011, 2890, 2848, 2778, 1603, 1591, 1500, 1473, 1450, 1435, 1380, 1335, 1243, 1219, 1104, 1035, 1002, 928, 822, 810, 762, 731 cm^{-1}; δ_H(400 MHz, CD_2Cl_2): 7.45 (1H, ddd, *J* = 7.5, 1.4, 0.6 Hz, 6-H), 7.32 (1H, td, *J* = 7.4, 1.6 Hz, 5-H), 7.26 (1H, td, *J* = 7.4, 1.5 Hz, 4-H), 7.22 – 7.16 (1H, m, 3-H), 6.87 (1H, dd, *J* = 5.7, 0.5 Hz, 2’-H), 6.86 (1H, d, *J* = 0.4 Hz, 5’-H), 6.83 – 6.76 (1H, m, 6’-H), 6.00 (2H, s, OCH_2O), 3.77 (2H, s, $C\underline{H}_2NH_2$); δ_C(101 MHz, CD_2Cl_2): 147.9 (C-3’), 147.1 (C-4’), 141.5 (C-1 or C-2), 141.3 (C-1 or C-2), 135.5 (C-1’), 130.5 (C-3), 128.5 (C-6), 127.9 (C-5), 127.0 (C-4), 122.8 (C-6’), 110.1 (C-5’), 108.4 (C-2’), 101.7 (OCH_2O), 44.5 (CH_2NH_2); HRMS (EI): m/z $[M]^{\bullet+}$ calcd for $C_{14}H_{13}NO_2^{\bullet+}$ 227.0946; found 227.0941.

2-(4’-Methoxy-3’-methylphenyl)benzylamine (**15q**)

This compound was prepared in accordance with **General procedure 5** from nitrile **14q** (348 mg, 1.56 mmol), LAH (237 mg, 6.24 mmol) and $AlCl_3$ (832 mg, 6.24 mmol). Purification by FCC afforded the product as a yellow oil (229 mg, 1.01 mmol, 65%).

R_f = 0.14 (DCM/MeOH 1% + NEt_3 1%); IR (ATR): 3364, 3058, 3018, 2920, 2834, 1609, 1589, 1506, 1480, 1463, 1295, 1239, 1170, 1135, 1028, 887, 812, 763, 749, 689 cm^{-1}; δ_H(400 MHz, CD_2Cl_2): 7.48 – 7.39 (1H, m, 6-H), 7.31 (1H, td, *J* = 7.4, 1.7 Hz, 5-H), 7.26 (1H, td, *J* = 7.4, 1.6 Hz, 4-H), 7.21 – 7.18 (1H, m, 3-H), 7.16 – 7.10 (2H, m, 2’-H and 6’-H), 6.89 (1H, d, *J* = 8.1 Hz, 5’-H), 3.87 (3H, s, OCH_3), 3.77 (2H, s, $C\underline{H}_2NH_2$), 2.25 (3H, s, CH_3); δ_C(101 MHz, CD_2Cl_2): 157.4 (C-5’), 141.6 (C-1 and C-2), 133.6 (C-1’), 131.8

(C-2’ or C-6’), 130.5 (C-3), 128.4 (C-6), 127.8 (C-2’ or C-6’), 127.6 (C-5), 126.9 (C-4), 126.6 (C-6’), 110.0 (C-4’), 55.7 (OCH_3), 44.6 (CH_2NH_2), 16.4 (CH_3); HRMS (EI): m/z $[M]^{•+}$ calcd for $C_{15}H_{17}NO^{•+}$ 227.1310; found 227.1301.

2-[4-(4-Phenyl)-phenyl]benzylamine (**15r**)

This compound was prepared in accordance with **General procedure 5** from nitrile **14r** (1.12 g, 4.40 mmol), LAH (668 mg, 17.6 mmol) and $AlCl_3$ (2.34 g, 17.6 mmol). Purification by FCC afforded the product as a yellow-white solid (1.05 g, 4.05 mmol, 92%).

R_f = 0.23 (DCM/MeOH 1% + NEt_3 1%); Mp = 80 – 82 °C; IR (ATR): 3374, 3026, 2908, 1597, 1475, 1450, 1398, 1336, 1248, 1159, 1004, 880, 847, 749, 697 cm^{-1}; δ_H(400 MHz, DMSO-*d*$_6$): 7.77 – 7.70 (4H, m, 2’-H and 6’-H and 2’’-H and 6’’-H), 7.60 (1H, dd, *J* = 7.6, 1.4 Hz, 3-H), 7.53 – 7.42 (4H, m, 3’-H and 5’-H and 3’’-H and 5’’-H), 7.38 (2H, tdd, *J* = 7.5, 3.6, 1.4 Hz, 4-H and 4’-H), 7.30 (1H, td, *J* = 7.4, 1.4 Hz, 5-H), 7.23 (1H, dd, *J* = 7.6, 1.5 Hz, 6-H), 3.68 (2H, s, C$\underline{H}_2$NH$_2$); δ_C(101 MHz, DMSO-*d*$_6$): 141.1 (C-2), 140.0 (C-1 or C-1’), 140.0 (C-1 or C-1’), 139.8 (C-4’), 138.7 (C-1’’), 129.6 (C-3’’ and C-5’’), 129.4 (C-6), 129.0 (C-3’ and C-5’), 128.3 (C-3), 127.5 (C-4 or C-4’), 127.4 (C-4 or C-4’), 126.6 (C-2’’ and C-6’’), 126.5 (C-2’ and C-6’’), 126.3 (C-5), 43.2 (CH_2NH_2); HRMS (ESI): m/z $[M - H]^-$ calcd for $C_{19}H_{17}N^-$ 258.1288; found 258.1279.

2-(4’-Methoxy-3’,5’-dimethylphenyl)benzylamine (**15s**)

This compound was prepared in accordance with **General procedure 5** from nitrile **14s** (339 mg, 1.43 mmol), LAH (217 mg, 5.72 mmol) and $AlCl_3$ (763 mg, 5.72 mmol). Purification by FCC afforded the product as a yellow oil (209 mg, 0.866 mmol, 61%).

R_f = 0.15 (DCM/MeOH 1% + NEt_3 1%); IR (ATR): 3353, 2922, 2859, 2825, 1588, 1466, 1412, 1371, 1324, 1230, 1199, 1164, 1101, 1077, 1007, 876, 821, 760 cm^{-1}; δ_H(400 MHz, CD_2Cl_2): 7.48 – 7.41 (1H, m, 6-H), 7.31 (1H, td, *J* = 7.5, 1.6 Hz, 5-H), 7.26 (1H, td, *J* = 7.4, 1.5 Hz, 4-H), 7.18 (1H, dd, *J* = 7.4, 1.6 Hz, 3-H), 6.97 (2H, s, 2’-H and 6’-H), 3.77 (2H, s, C$\underline{H}_2$NH$_2$), 3.75 (3H, s, OCH_3), 2.31 (6H, s, CH_3); δ_C(101 MHz, CD_2Cl_2): 156.6 (C-4’), 141.6 (C-1 or C-2), 141.4 (C-1 or C-2), 137.0 (C-1’), 131.0 (C-3’ and C-5’), 130.4 (C-3), 129.9 (C-2’ and C-6’), 128.3 (C-6), 127.7 (C-5), 126.8 (C-4), 60.0 (OCH_3), 44.5 (CH_2NH_2), 16.3 (3’-CH_3 and 5’-CH_3); HRMS (EI): m/z $[M]^{•+}$ calcd for $C_{16}H_{19}NO^{•+}$ 241.1467; found 241.1458.

2-(4’-Hydroxyphenyl)benzylamine (**15t**)

This compound was prepared in accordance with **General procedure 4** from nitrile **14t** (390 mg, 2.00 mmol), LAH (342 mg, 9.00 mmol) and $AlCl_3$ (667 mg, 5.00 mmol). Purification by FCC afforded the product as a yellow white powder (222 mg, 1.11 mmol, 56%).

R_f = 0.49 (DCM/MeOH 1% + NEt_3 2%); MP = 180 – 182 °C; IR (ATR): 3325, 2377, 2978, 2945, 2601, 2496, 1607, 1584, 1515, 1473, 1445, 1397, 1259, 1245, 1169, 1101, 1035, 941, 836, 804, 758 cm^{-1}; δ_H(400 MHz, DMSO-*d*$_6$): 7.56 (1H, dd, *J* = 7.7, 1.5 Hz, 6-H), 7.32 (1H, td, *J* = 7.5, 1.6 Hz, 4-H), 7.27 (1H, td, *J* = 7.4, 1.6 Hz, 5-H), 7.19 – 7.14 (3H, m, 3-H and 2’-H and 6’-H), 6.88 – 6.79 (2H, m, 3’-H and 5’-H), 3.71

(2H, s, $C\underline{H}_2NH_2$); ^{13}C NMR (101 MHz, DMSO-d_6) δ = 156.6 (C-5), 140.8 (C-2), 138.7 (C-1), 131.1 (C-1'), 130.1 (C-2' and C-6'), 129.6 (C-3), 128.1 (C-6), 126.9 (C-4), 126.7 (C-5), 115.0 (C-3' and C-5'), 42.3 (CH_2NH_2); HRMS (EI): m/z [M - H]$^-$ calcd for $C_{13}H_{12}NO^-$ 198.0924; found 198.0911.

2-(4'-((*tert*- Butyldimethylsilyl)oxy)phenyl)benzylamine (**15u**)

This compound was prepared in accordance with **General procedure 5** from nitrile **14u** (501 mg, 1.62 mmol), LAH (215 mg, 5.67 mmol), $AlCl_3$ (648 mg, 4.86 mmol). Purification by FCC afforded the product as a white-yellow solid (380 mg, 1.21 mmol, 75%).

R_f = 0.15 (DCM/MeOH 1% + NEt_3 1%); Mp = 56 °C; IR (ATR): 2954, 2928, 2857, 1606, 1512, 1478, 1248, 1168, 1097, 1005, 911, 835, 775, 764, 683 cm^{-1}; δ_H(400 MHz, CD_2Cl_2): 7.48 – 7.43 (1H, m, 6-H), 7.32 (1H, td, *J* = 7.4, 1.6 Hz, 5-H), 7.27 (1H, td, *J* = 7.4, 1.6 Hz, 4-H), 7.24 – 7.19 (3H, m, 3-H and 2'-H and 6'-H), 6.94 – 6.86 (2H, m, 3'-H and 5'-H), 3.77 (2H, s, $C\underline{H}_2NH_2$), 1.02 (9H, s, $C(C\underline{H}_3)_3$), 0.25 (6H, s, $Si(C\underline{H}_3)_2$); δ_C(101 MHz, CD_2Cl_2): 155.3 (C-4'), 141.5 (C-1 or C-2), 141.4 (C-1 or C-2), 134.6 (C-1'), 130.6 (C-2' and C-6'), 130.5 (C-3), 128.4 (C-6), 127.7 (C-5), 126.9 (C-4), 120.1 (C-3' and C-5'), 44.5 (CH_2NH_2), 25.9 ($C(\underline{C}H_3)_3$), 18.5 ($\underline{C}(CH_3)_3$, -4.3 ($Si(\underline{C}H_3)_2$); HRMS (ESI): m/z [M + H]$^+$ calcd for $C_{19}H_{28}NOSi^+$ 314.1935; found 314.1938.

2-(4'-((2- (Trimethylsilyl)ethoxy)methoxy)phenyl)benzylamine (**15v**)

This compound was prepared in accordance with **General procedure 5** nitrile **14v** (195 mg, 0.600 mmol), LAH (68.3 mg, 1.80 mmol) and $AlCl_3$ (240 mg, 1.80 mmol). Purification by FCC afforded the product as a yellow oil (136 mg, 0.413 mmol, 69%).

R_f = 0.14 (DCM/MeOH 1% + NEt_3 1%); IR (ATR): 3346, 3013, 2938, 2897, 1605, 1504, 1486, 1434, 1221, 1167, 1087, 1019, 980, 945, 934, 867, 825, 743, 700 cm^{-1}; δ_H(400 MHz, CD_2Cl_2): 7.48 – 7.41 (1H, m, H_{arom}), 7.35 – 7.19 (5H, m, H_{arom}), 7.10 – 7.04 (2H, m, 3'-H and 5'-H), 5.25 (2H, d, *J* = 2.4 Hz, $OC\underline{H}_2O$), 4.35 – 4.27 (2H, m, $C\underline{H}_2NH_2$), 3.83 – 3.75 (2H, m, $OC\underline{H}_2CH_2$), 1.03 – 0.93 (2H, m, $OCH_2C\underline{H}_2$), 0.02 (9H, d, *J* = 1.3 Hz, $Si(C\underline{H}_3)_3$); δ_C(101 MHz, CD_2Cl_2): 157.1 (C-4'), 141.7 (C_{arom}), 139.0 (C_{arom}), 131.0 (C-2' and C-6'), 130.7 (C_{arom}), 130.7 (C_{arom}), 129.8 (C_{arom}), 127.7 (C_{arom}), 127.0 (C_{arom}), 116.3 (C-3' and C-5'), 93.6 (OCH_2O), 66.8 ($O\underline{C}H_2CH_2$), 53.8 (CH_2NH_2), 18.6 ($OCH_2\underline{C}H_2$), -1.2 ($Si(\underline{C}H_3)_3$); HRMS (EI): m/z [M + H]$^+$ calcd for $C_{19}H_{28}NO_2Si^+$ 330.1884; found 330.1886.

2-(4'-Phenylmethoxyphenyl)benzylamine (**15w**)

This compound was prepared in accordance with **General procedure 5** from nitrile **14w** (380 mg, 1.33 mmol), LAH (151 mg, 3.99 mmol) and $AlCl_3$ (248 mg, 1.86 mmol). Purification by FCC afforded the product as a yellow solid (248 mg, 0.857 mmol, 64%).

R_f = 0.23 (DCM/MeOH 1% + NEt_3 1%); IR (ATR): 3035, 2916, 2874, 1606, 1576, 1514, 1496, 1387, 1232, 1178, 1117, 1025, 1012, 999, 840, 756, 699 cm^{-1}; δ_H(400 MHz, CD_2Cl_2): 7.47 (3H, dddd, *J* = 8.9, 8.0, 1.6, 0.8 Hz, 2''-H and 4''-H and 6''-H), 7.44 – 7.39 (2H, m, 3''-H and 5''-H), 7.38 – 7.32 (2H, m, 4-H and 3-H or 5-H), 7.32 – 7.24 (3H, m, 2'-H and 6'-H and 3-H or 5-H), 7.23 – 7.20 (1H, m, 6-H), 7.08 – 6.99 (2H,

m, 3’-H and 5’-H), 5.12 (2H, s, OCH_2), 3.78 (2H, s, C$\underline{H}_2$$NH_2$); δ_C(101 MHz, CD_2Cl_2): 158.4 (C-4’), 141.6 (C-2), 141.3 (C-1), 137.6 (C-1’’), 134.3 (C-1’), 130.6 (C-2’ and C-6’), 130.5 (C-6), 128.9 (C-3’’ and C-5’’), 128.5 (C-3 or C-5), 128.4 (C-3 or C-5), 128.0 (C-2’’ and C-6’’), 127.7 (C-4), 126.9 (C-4’’), 114.9 (C-3’ and C-5’), 70.4 (OCH_2), 44.6 (CH_2NH_2); HRMS (EI): m/z $[M]^{\bullet+}$ calcd for $C_{20}H_{19}NO^{\bullet+}$ 289.1467; found 289.1462.

(4-bromo-3-methoxyphenoxy)(tert-butyl)dimethylsilane (**17**)

This compound was prepared in accordance with **General procedure 6** from phenol **16** (8.33 g, 41.0 mmol), imidazole (6.98 g, 103 mmol) and TBS-Cl (8.65 g, 57.4 mmol). Purification by FCC afforded the product as a colorless oil (12.6 g, 39.8 mmol, 97%).

R_f = 0.51 (hexanes/EtOAc 20:1); IR = 2955, 2930, 2886, 2858, 1587, 1484, 1471, 1463, 1447, 1403, 1298, 1254, 1202, 1168, 1051, 975, 834, 778, 703, 669 cm^{-1}; δ_H(500 MHz, CD_2Cl_2): 7.33 (1H, d, *J* = 8.5 Hz, 5-H), 6.44 (1H, d, *J* = 2.6 Hz, 2-H), 6.36 (1H, dd, *J* = 8.5, 2.6 Hz, 6-H), 3.83 (3H, s, OCH_3), 0.98 (9H, s, $C(C\underline{H}_3)_3$), 0.21 (6H, s, $Si(C\underline{H}_3)_2$); δ_C(126 MHz, CD_2Cl_2): 157.1 (C-1 or C-3), 157.0 (C-1 or C-3), 133.5 (C-5), 113.8 (C-6), 105.7 (C-2), 103.4 (C-4), 56.6 (OCH_3), 26.0 ($C(\underline{C}H_3)_3$), 18.6 ($\underline{C}(CH_3)_3$), -4.2 ($Si(\underline{C}H_3)_2$); HRMS (EI): m/z $[M]^{\bullet+}$ calcd for $C_{13}H_{21}BrO_2Si^{\bullet+}$ 316.0494; found 316.0499. Data in accordance with literature values.[30]

(4-{[*tert*-Butyl(dimethyl)silyl]oxy}-2-methoxyphenyl)boronic acid (**18)**

A solution of *n*-butyllithium (2.5 M, 17.2 mL, 43.0 mmol, 1.1 equiv) in hexane was added dropwise to a solution of aryl bromide **17** (12.4 g, 39.1 mmol, 1.0 equiv) in THF (40 mL) under a N_2 atmosphere at -78 °C. The mixture was stirred stirred for 1.5 h after which B(O*i*Pr)$_3$ (11.0 mL, 46.9 mmol, 1.2 equiv) was added. The mixture was stirred for an additional 2.5 h and then quenched with a solution of saturated NH_4Cl (40 mL). After allowing the mixture to warm to room temperature H_2O (50 mL) was added. The pH was adjusted to 5.0 with 1 M HCl and the resulting solution extracted with EtOAc (3 x 40 mL). The combined organic layers were washed with brine (3 x 20 mL), dried over $MgSO_4$ and the solvent removed *in vacuo*. Purification by FCC afforded the product as an off-white solid (7.23 g, 25.6 mmol, 66%).

R_f = 0.37 (hexanes/EtOAc 4:1); Mp = 124 °C; IR (ATR): 3356, 3200, 2957, 2928, 2856, 1597, 1561, 1455, 1418, 1361, 1340, 1293, 1253, 1205, 1157, 1109, 1082, 1039, 979, 838, 777, 664 cm^{-1}; δ_H(500 MHz, CD_2Cl_2): 7.21 (1H, d, *J* = 8.1 Hz, 6-H), 6.07 (1H, dd, *J* = 8.1, 2.0 Hz, 5-H), 5.99 (1H, d, *J* = 2.0 Hz, 3-H), 5.32 (2H, s, $B(OH)_2$), 3.43 (3H, s, OCH_3), 0.56 (9H, s, $C(C\underline{H}_3)_3$), -0.20 (6H, s, $Si(C\underline{H}_3)_2$); δ_C(126 MHz, CD_2Cl_2): 166.6 (C-2), 160.5 (C-4), 137.9 (C-6), 113.0 (C-1 and C-5), 103.3 (C-3), 55.8 (OCH_3), 25.8 ($C(\underline{C}H_3)_3$), 18.5 ($\underline{C}(CH_3)_3$), -4.3 ($Si(\underline{C}H_3)_2$); HRMS (EI): m/z $[M]^{\bullet+}$ calcd for $C_{13}H_{23}BO_4Si^{\bullet+}$ 282.1459; found 282.1448. Data in accordance with literature values.[31]

2-Bromo-5-hydroxybenzonitrile (**20**)

3-Hydroxybenzonitrile (**19**) (2.50 g, 21.0 mmol, 1.00 equiv) was dissolved in MeCN (20 mL) and cooled to -20 °C. $BF_3{\cdot}OEt_2$ (2.59 mL, 21.0 mmol, 1.00 equiv) followed by NBS (3.74 g, 21.0 mmol, 1.00 equiv)

were added and the mixture allowed to warm to ambient temperature. The resulting mixture was stirred at this temperature for 12 h, then treated with aqueous NH_4Cl solution (100 mL) and H_2O (100 mL), and extracted with EtOAc (3 x 30 mL). The combined organic layers were washed with brine (3 x 20 mL) and dried over anhydrous $MgSO_4$. After filtration, the solvent was removed *in vacuo*. Purification by FCC afforded the product as a light yellow solid (2.13 g, 10.8 mmol, 51 %).

R_f = 0.29 (hexanes/EtOAc 4:1);Mp = 181-182 °C; IR (ATR): 3344, 3101, 2237, 1591, 1487, 1472, 1427, 1304, 1232, 1173, 1125, 964, 860. 836, 785, 691 cm^{-1}; ^1H NMR (400 MHz, DMSO-d_6) δ = 10.49 (s, 1H, OH), 7.63 (d, *J* = 8.9 Hz, 1H, 3-H), 7.24 (d, *J* = 2.9 Hz, 1H, 6-H), 7.04 (dd, *J* = 8.9, 3.0 Hz, 1H, 4-H); ^{13}C NMR (101 MHz, DMSO-d_6) δ = 157.1 (C-5), 134.2 (C-3), 122.6 (C-4), 120.9 (C-6), 117.2 (C-2), 114.8 (C-1), 112.6 (CN); HRMS (EI): $[M]^{•+}$ calcd for $C_7H_4{}^{79}BrNO^{•+}$ 196.9476; found 196.9472. Data in accordance with literature values.[32]

5-Hydroxy-2-(2’-methoxy-4’-hydroxyphenyl)benzonitrile (**21**)

This compound was prepared in accordance with **General procedure 4** from aryl bromide **20** (3.25 g, 16.4 mmol), boronic acid **18** (7.22 g, 1.56 mmol), $Pd(PPh_3)_4$ (948 mg, 0.820 mmol) and K_2CO_3 (8.13 g, 49.2 mmol). Purification by FCC afforded the product as a white solid (1.61 g, 6.67 mmol, 41%).

R_f = 0.30 (hexanes/EtOAc 1:1); Mp = 234 °C; IR (ATR): 3329, 3255, 2929, 2233, 1606, 1594, 1471, 1442, 1364, 1214, 1293, 1222, 1194, 1165, 1129, 1035, 960, 946, 852, 830, 822, 800 cm^{-1}; δ_H(400 MHz, DMSO-d_6): 9.87 (2H, s, 5-OH and 4’-OH), 7.21 (1H, d, *J* = 8.4 Hz, 3-H), 7.11 (1H, d, *J* = 2.5 Hz, 6-H), 7.07 (1H, dd, *J* = 8.4, 2.6 Hz, 4-H), 6.98 (1H, d, *J* = 8.2 Hz, 6’-H), 6.50 (1H, d, *J* = 2.2 Hz, 3’-H), 6.43 (1H, dd, *J* = 8.2, 2.2 Hz, 5’-H), 3.68 (3H, s, OCH_3); δ_C(101 MHz, DMSO-d_6): 159.0 (C-4’), 157.3 (C-2’), 156.2 (C-5), 133.0 (C-2), 132.4 (C-3), 131.3 (C-6’), 120.3 (C-4), 118.6 (C-1’), 118.4 (C-6), 117.7 (CN), 112.9 (C-1), 107.2 (C-5’), 99.3 (C-3’), 55.1 (OCH_3); HRMS (EI): m/z $[M]^{•+}$ calcd for $C_{14}H_{11}NO_3{}^{•+}$ 241.0739; found 241.0741.

4,4'-Bis((*tert*-butyldimethylsilyl)oxy)-2'-methoxy-[1,1'-biphenyl]-2-carbonitrile (**22**)

This compound was prepared in accordance with **General procedure 6** from diphenol **21** (1.61 g, 6.67 mmol), TBS-Cl (2.81 g, 18.7 mmol) and imidazole (2.27 g, 33.4 mmol). Purification by FCC afforded the product as a clear oil (2.73 mg, 5.81 mmol, 87%).

R_f = 0.52 (hexanes/EtOAc 10:1); IR (ATR): 2957, 2929, 2885, 2857, 2234, 1602, 1577, 1482, 1472, 1461, 1444, 1407, 1284, 1252, 1198, 1161, 1037, 976, 832, 777, 679 cm^{-1}; δ_H(400 MHz, CD_2Cl_2): 7.28 (1H, d, *J* = 8.5 Hz, 3-H), 7.15 (1H, d, *J* = 2.6 Hz, 6-H), 7.11 – 7.05 (2H, m, 2’-H and 4-H), 6.53 (2H, d, *J* = 7.2 Hz, 3’-H and 5’-H), 3.78 (3H, s, OCH_3), 1.02 (9H, s, 5-$(CH_3)_3$ or 4’-$C(CH_3)_3$), 1.01 (9H, s, 5-$(CH_3)_3$ or 4’-$C(CH_3)_3$), 0.26 (6H, s, 5-$Si(CH_3)_2$ or 4’-$Si(C\underline{H}_3)_2$), 0.25 (6H, s, 6H, s, 5-$Si(CH_3)_2$ or 4’-$Si(C\underline{H}_3)_2$); δ_C(101 MHz, CD_2Cl_2): 158.2 (C-6’), 158.0 (C-4’), 155.0 (C-5), 136.0 (C-2), 132.9 (C-3), 131.9 (C-4 or C-6’), 125.1 (C-4 or C-6’), 124.2 (C-6), 120.9 (C-1’), 119.0 (CN), 114.5 (C-1), 112.3 (C-3’ or C-5’), 104.5 (C-3’ or C-5’), 55.9 (OCH_3), 26.0 (5-$C(\underline{C}H_3)_3$ or 4’-$(\underline{C}H_3)_3$), 25.9 (5-$C(\underline{C}H_3)_3$ or 4’-$(\underline{C}H_3)_3$), 18.7 (5-$\mathit{C}(CH_3)_3$ or

4'-*C*(CH_3)$_3$), 18.6 (5-C̲(CH_3)$_3$ or 4'-C̲(CH_3)$_3$), -4.1 (5-Si(C̲H_3)$_2$ or 4'-Si(C̲H_3)$_2$), -4.2 (5-Si(C̲H_3)$_2$ or 4'-Si(C̲H_3)$_2$); HRMS (ESI): m/z [M + H]$^+$ calcd for $C_{26}H_{41}NO_3Si_2^+$ 470.2547; found 470.2545.

(4,4'-Bis((*tert*-butyldimethylsilyl)oxy)-2'-methoxy-[1,1'-biphenyl]-2-yl)methanamine (**23**)

This compound was prepared in accordance with **General procedure 5** from nitrile **22** (2.73 g, 5.81 mmol), LAH (882 mg, 23.2 mmol) and $AlCl_3$ (3.10 g, 23.2 mmol). Purification by FCC afforded the product as a white solid (2.20 g mg, 4.64 mmol, 80%).

R_f = 0.15 (DCM/MeOH 1% + NEt_3 1%); Mp = 98 °C; IR (ATR): 2956, 2928, 2893, 2858, 1600, 1568, 1472, 1460, 1404, 1276, 1251, 1196, 1160, 1116, 1034, 973, 836, 774 cm^{-1}; δ_H(400 MHz, CD_2Cl_2): 6.97 (1H, d, *J* = 8.2 Hz, 3-H), 6.94 (1H, dd, *J* = 7.6, 0.7 Hz, 1-H), 6.92 (1H, d, *J* = 2.6 Hz, 6-H), 6.72 (1H, dd, *J* = 8.2, 2.6 Hz, 4-H), 6.48 (2H, d, *J* = 7.7 Hz, 3'-H and 5'-H), 3.71 (3H, s, OCH_3), 3.51 (2H, s, 5-Si(C̲H_3)$_2$ or 4'-Si(C̲H_3)$_2$), 1.01 (18H, d, *J* = 0.9 Hz, 5-C(CH̲$_3$)$_3$ and 4'-C(CH̲$_3$)$_3$), 0.25 (14H, d, *J* = 2.6 Hz, 5-Si(CH̲$_3$)$_2$ or 4'-Si(CH̲$_3$)$_2$); δ_C(101 MHz, CD_2Cl_2): 157.5 (C-2'), 156.2 (C-4'), 155.1 (C-5), 144.2 (C-1), 131.5 (C-3 or C-6'), 131.5 (C-3 or C-6'), 130.4 (C-2), 122.8 (C-1'), 118.6 (C-6), 117.5 (C-4), 111.5 (C-3' or C-5'), 103.4 (C-3' or C-5'), 55.3 (OCH_3), 44.3 (CH_2NH_2), 25.4 (5-C(CH̲$_3$)$_3$ and 4'-C(CH̲$_3$)$_3$), 18.1 (d, *J* = 2.9 Hz, 5-C̲(CH_3)$_3$ and 4'-C̲(CH_3)$_3$), -4.7 (d, *J* = 1.3 Hz, 5-Si(CH̲$_3$)$_2$ and 4'-Si(CH̲$_3$)$_2$); HRMS (ESI): m/z [M + H]$^+$ calcd for $C_{26}H_{45}NO_3Si_2^+$ 474.2860; found 474.2858.

2,7-Bis((*tert*-butyldimethylsilyl)oxy)-4-methoxy-9*H*-fluoren-9-one (**24**)

This compound was prepared in accordance with **General procedure 3** from amine **23** (929 mg, 1.96 mmol) and aqueous TBHP (70%, 0.760 mL, 7.84 mmol). Purification by FCC proved difficult. The crude compound was used in the next reaction without further purification.

δ_H(400 MHz, CD_2Cl_2): 7.54 (1H, dd, *J* = 8.1, 0.5 Hz), 7.02 (1H, dd, *J* = 2.4, 0.5 Hz), 6.87 (1H, dd, *J* = 8.0, 2.4 Hz), 6.69 (1H, d, *J* = 1.9 Hz), 6.52 (1H, d, *J* = 2.0 Hz), 3.91 (3H, s), 1.00 (9H, s), 0.99 (9H, s), 0.24 (6H, s), 0.21 (6H, s); HRMS (EI): m/z [M]$^{•+}$ calcd for $C_{26}H_{38}O_4Si_2^{•+}$ 470.2309; found 471.2384.

2. ^{1}H and ^{13}C NMR spectra

2,7-Dihydroxy-4-methoxy-9*H*-fluoren-9-one (**1d**)

δ_H(400 MHz, DMSO-d_6)

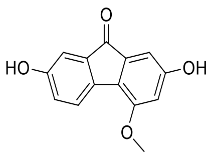

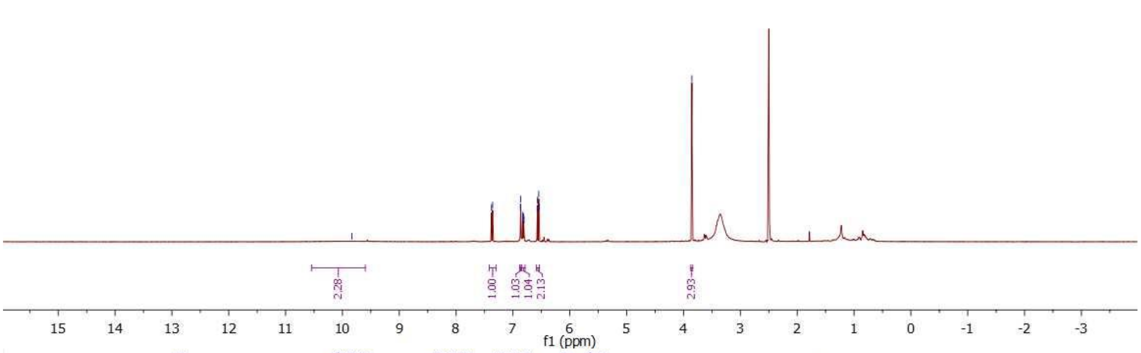

δ_C(101 MHz, DMSO-d_6)

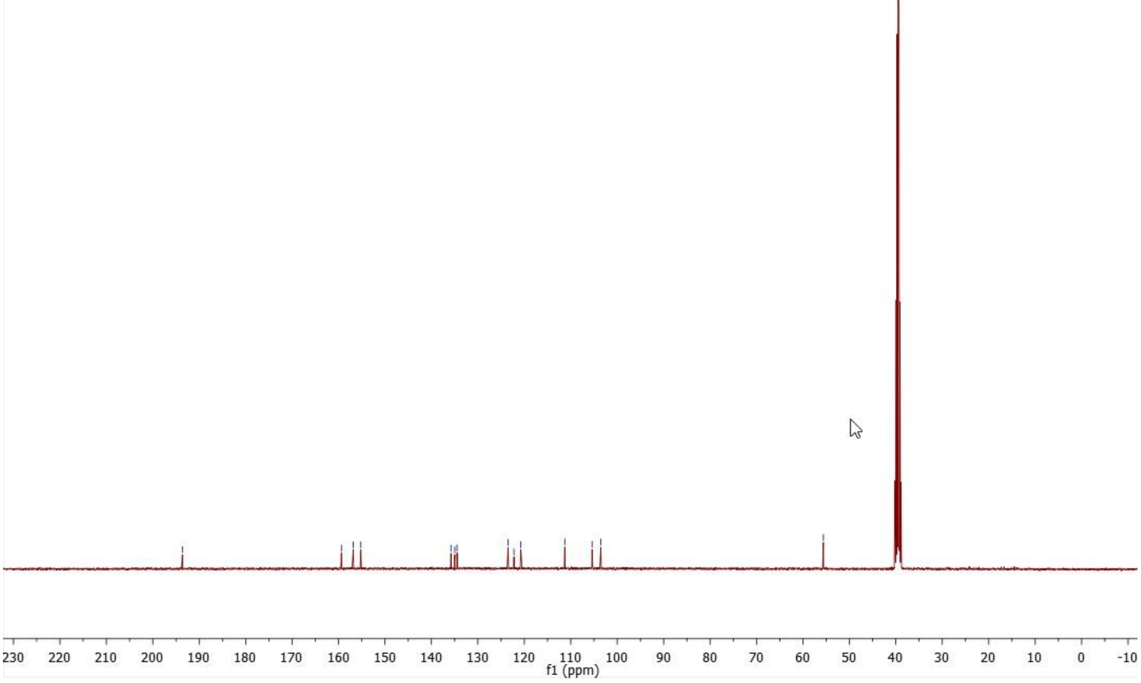

9*H*-Fluoren-9-one (**3**)

δ_H(400 MHz, CD_2Cl_2)

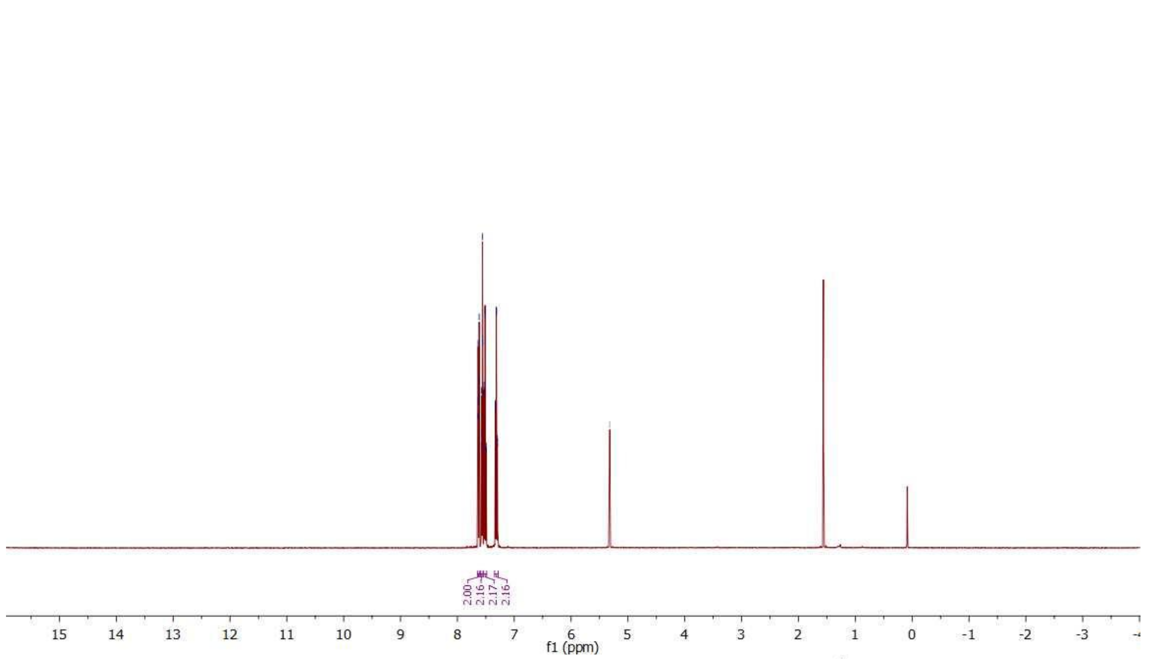

δ_C(101 MHz, CD_2Cl_2)

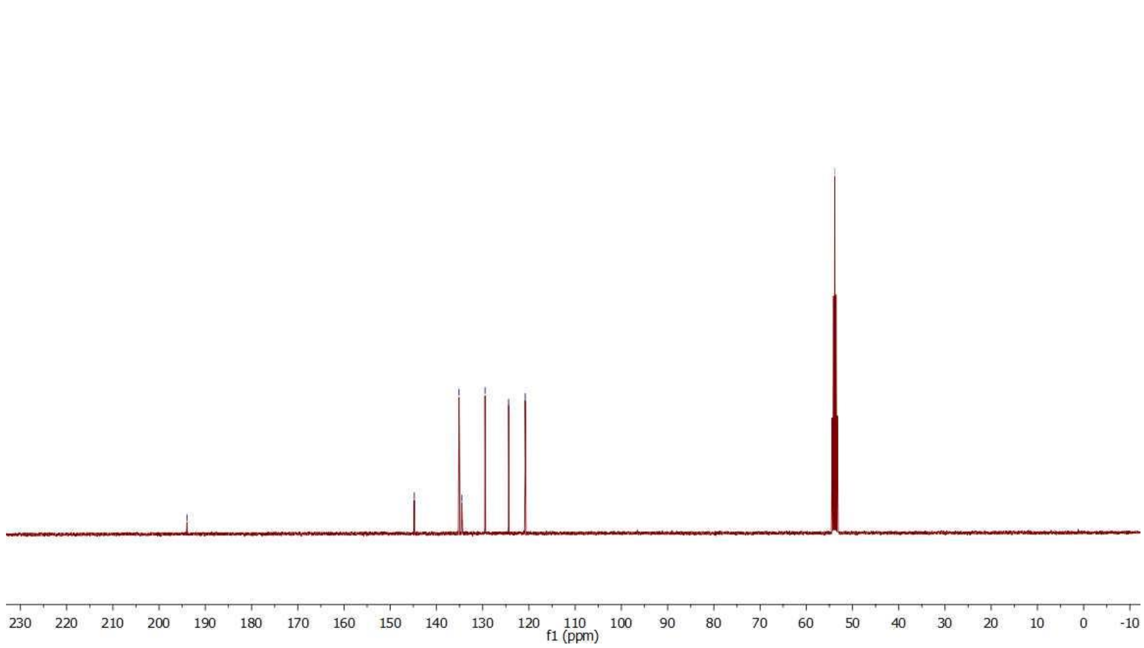

1,2,3-Trimethoxy-9*H*-fluoren-9-one (**10a**)

δ$_H$ (500 MHz, CD_2Cl_2)

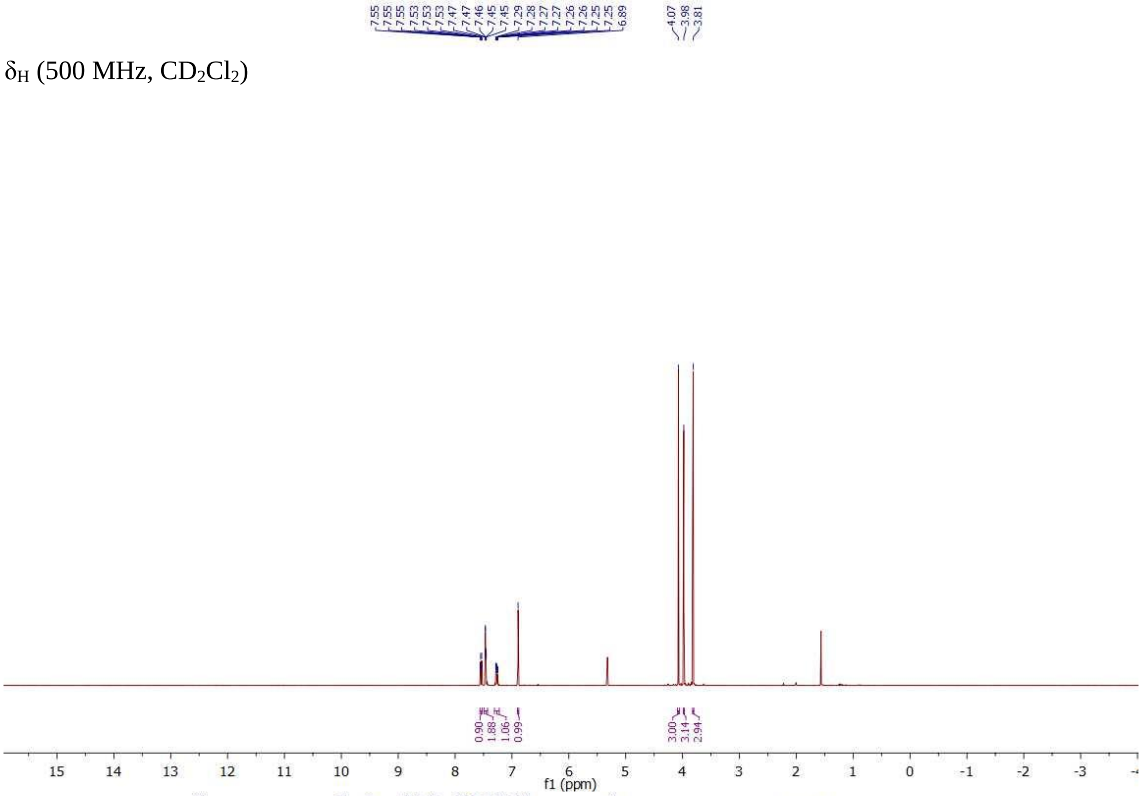

δ$_C$ (126 MHz, CD_2Cl_2)

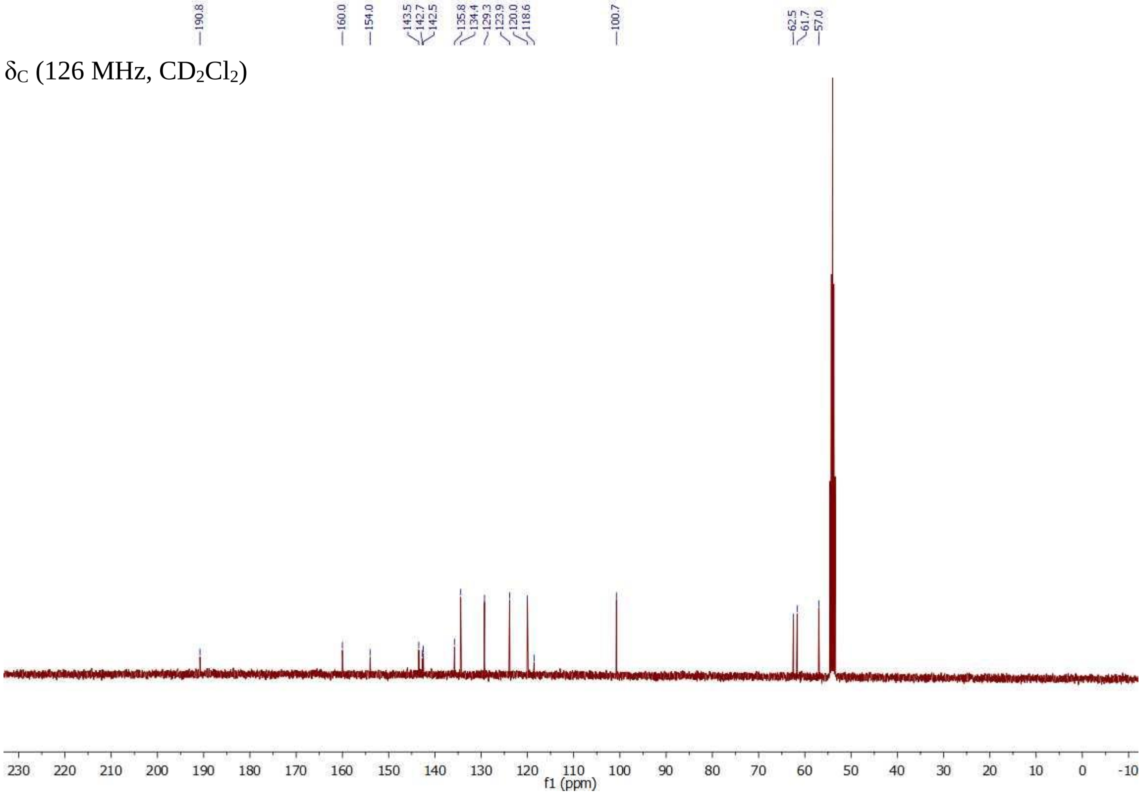

2-Methoxy-9*H*-fluoren-9-one (**10b**)

δ_H (400 MHz, CD_2Cl_2)

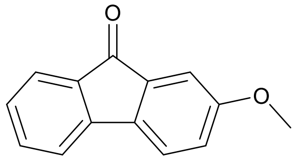

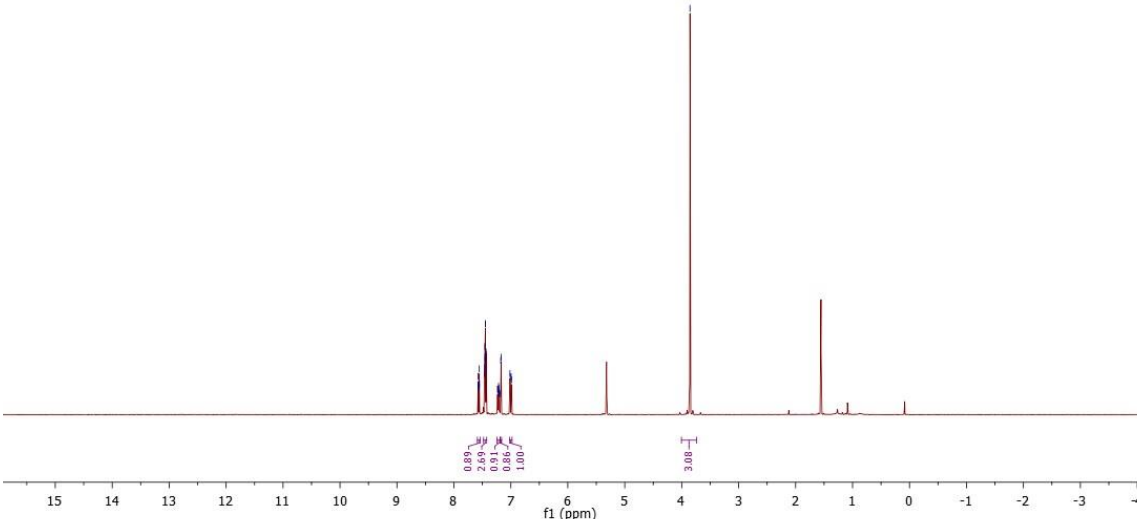

δ_C (101 MHz, CD_2Cl_2)

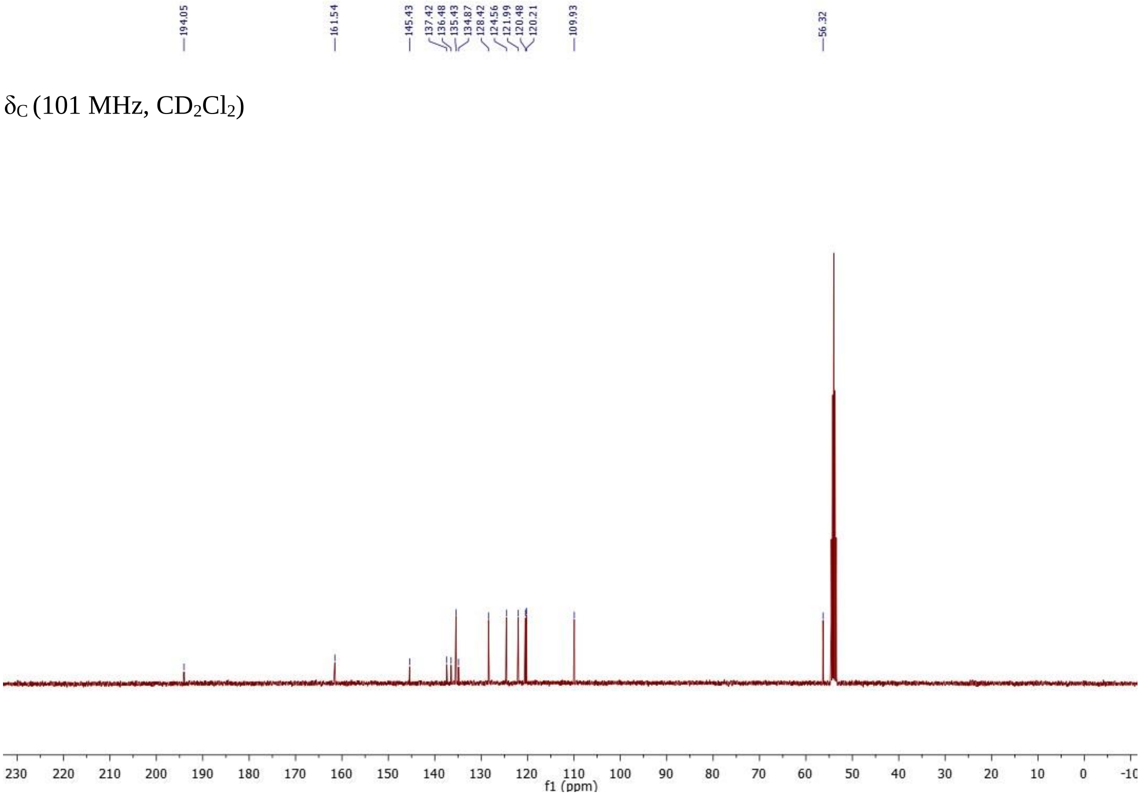

1,3-Dimethoxy-9*H*-fluoren-9-one (**10c**)

δ_H (500 MHz, CD_2Cl_2)

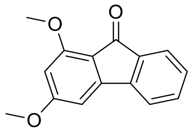

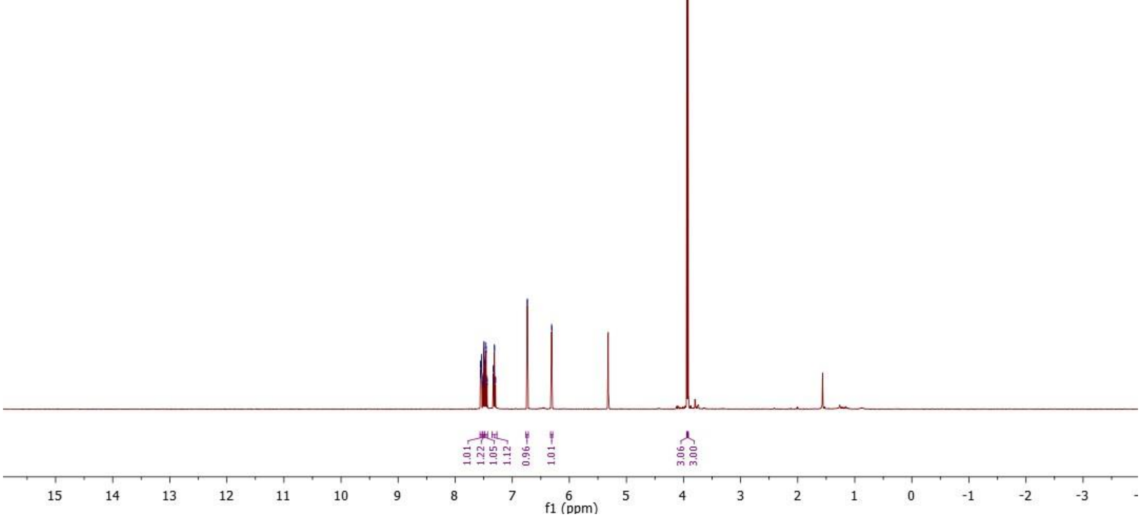

δ_C (126 MHz, CD_2Cl_2)

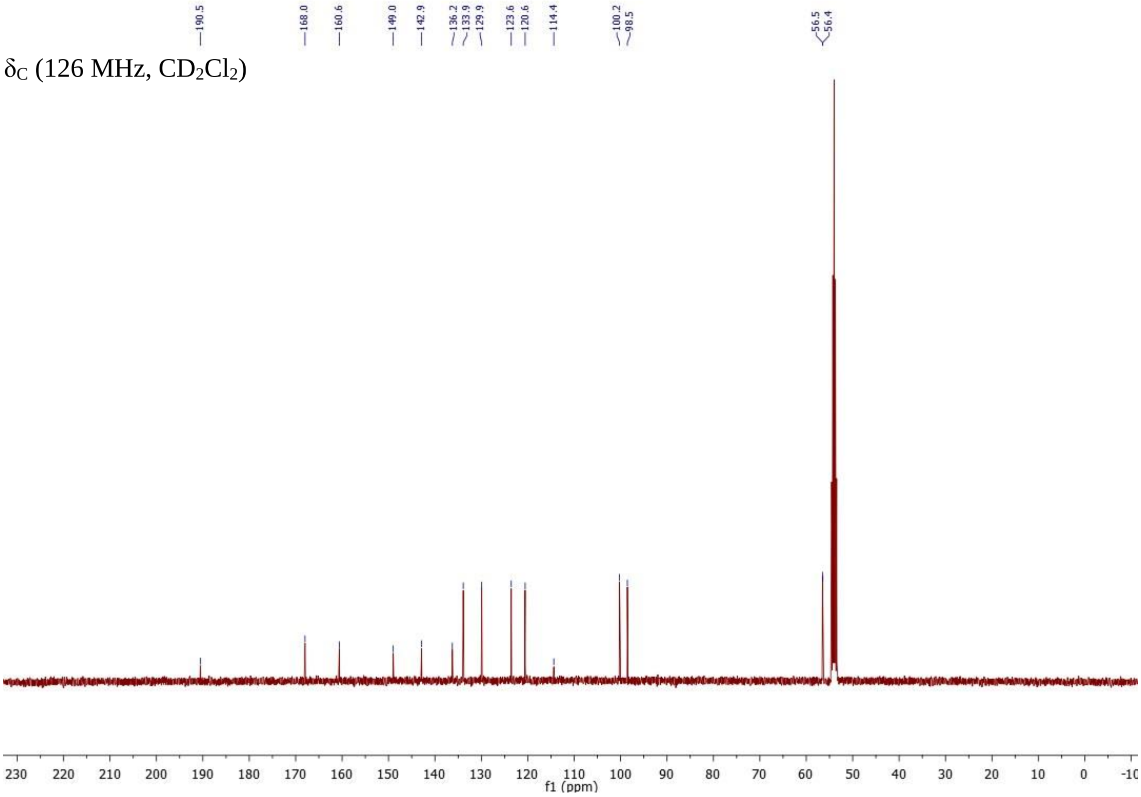

3-Methoxy-9*H*-fluoren-9-one (**10d**)

δ_H(400 MHz, CD_2Cl_2)

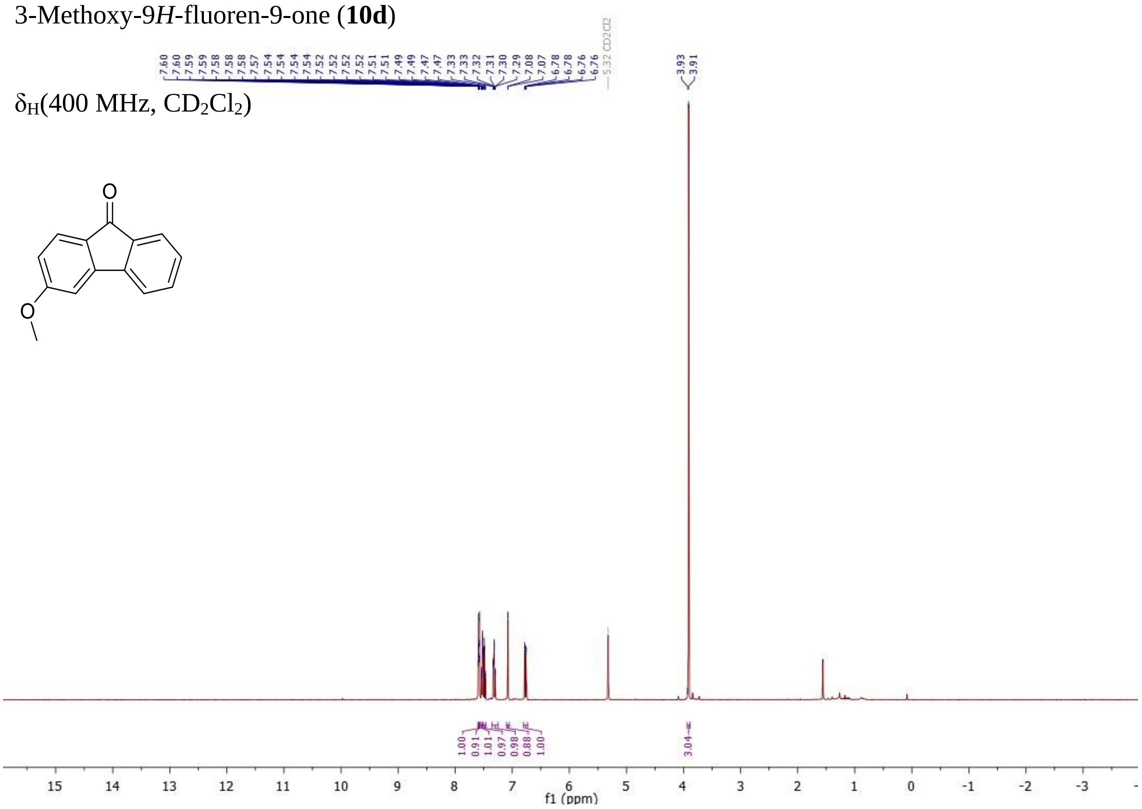

δ_C(101 MHz, CD_2Cl_2)

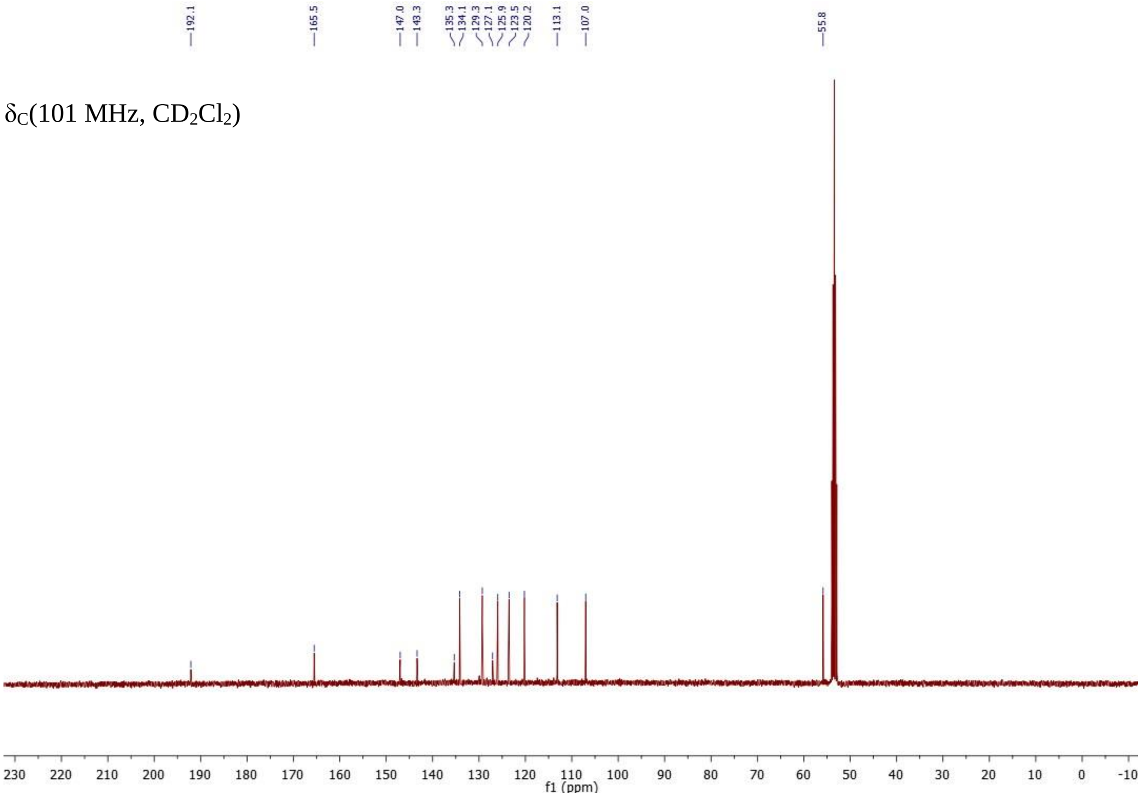

4-Methoxy-9*H*-fluoren-9-one (**10e**)

δ$_H$(400 MHz, CD_2Cl_2)

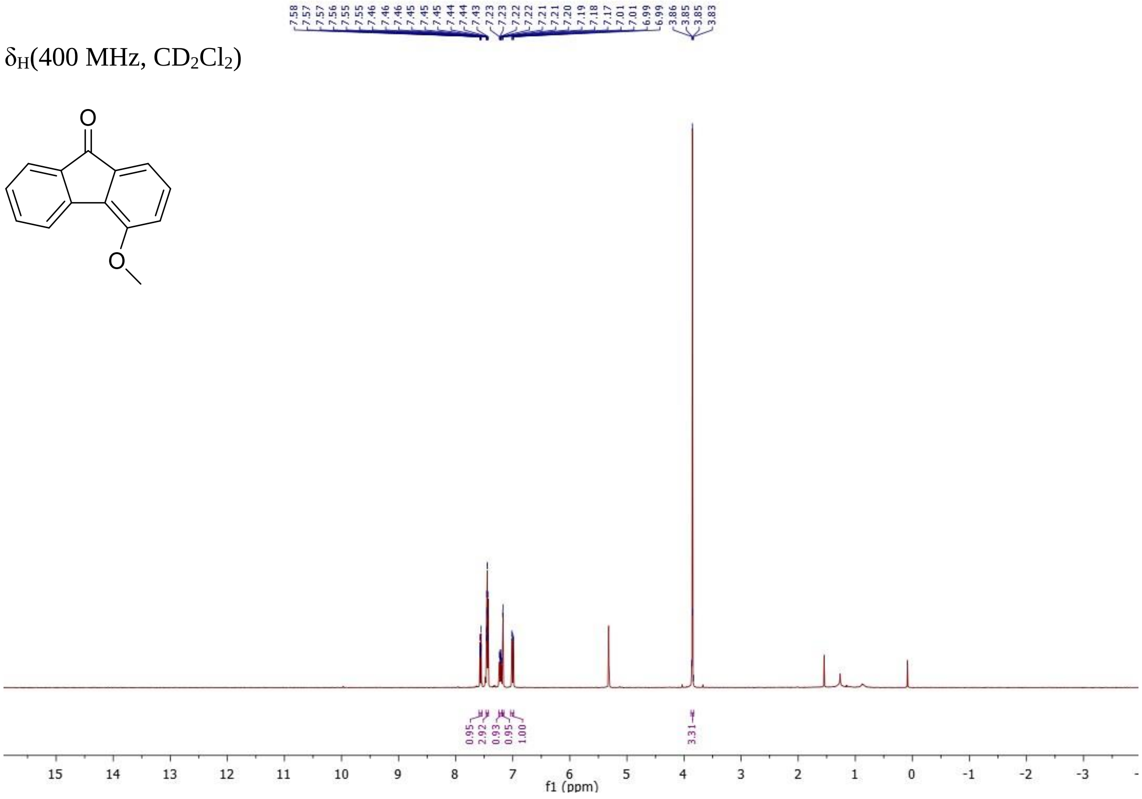

δ$_C$(101 MHz, CD_2Cl_2)

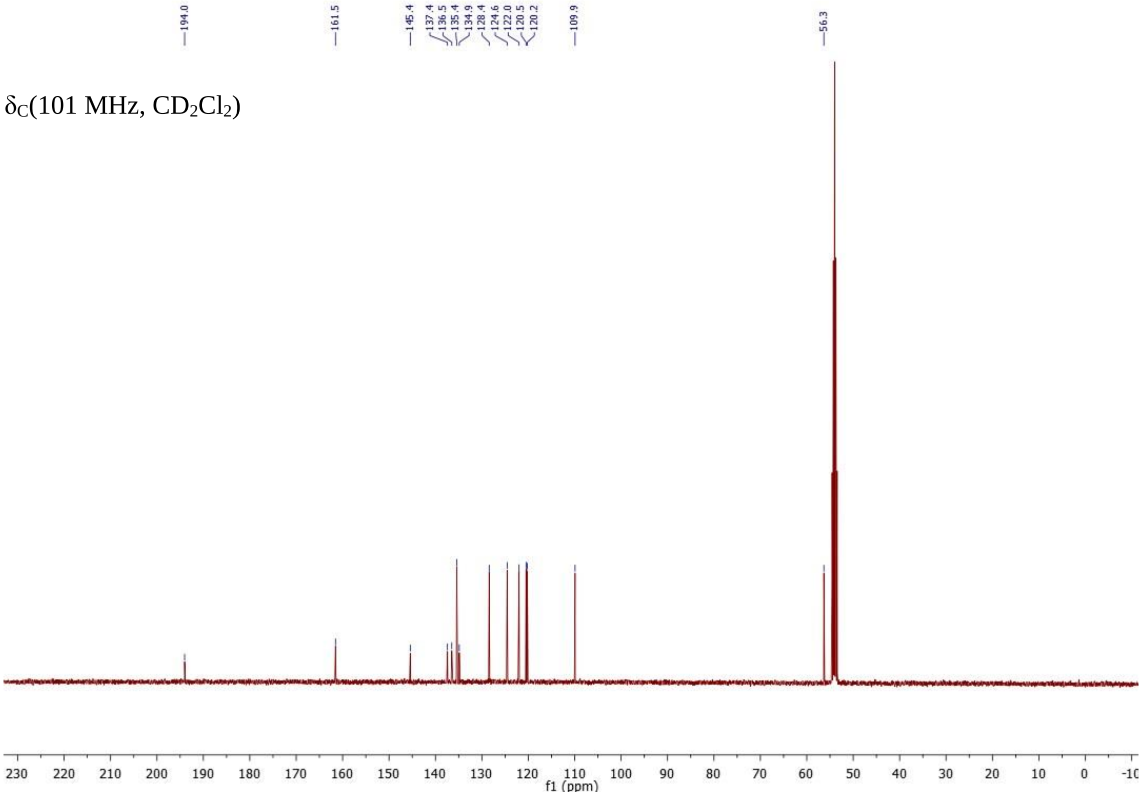

1-Methoxy-9*H*-fluoren-9-one (**10f**)

δ_H(400 MHz, CD_2Cl_2)

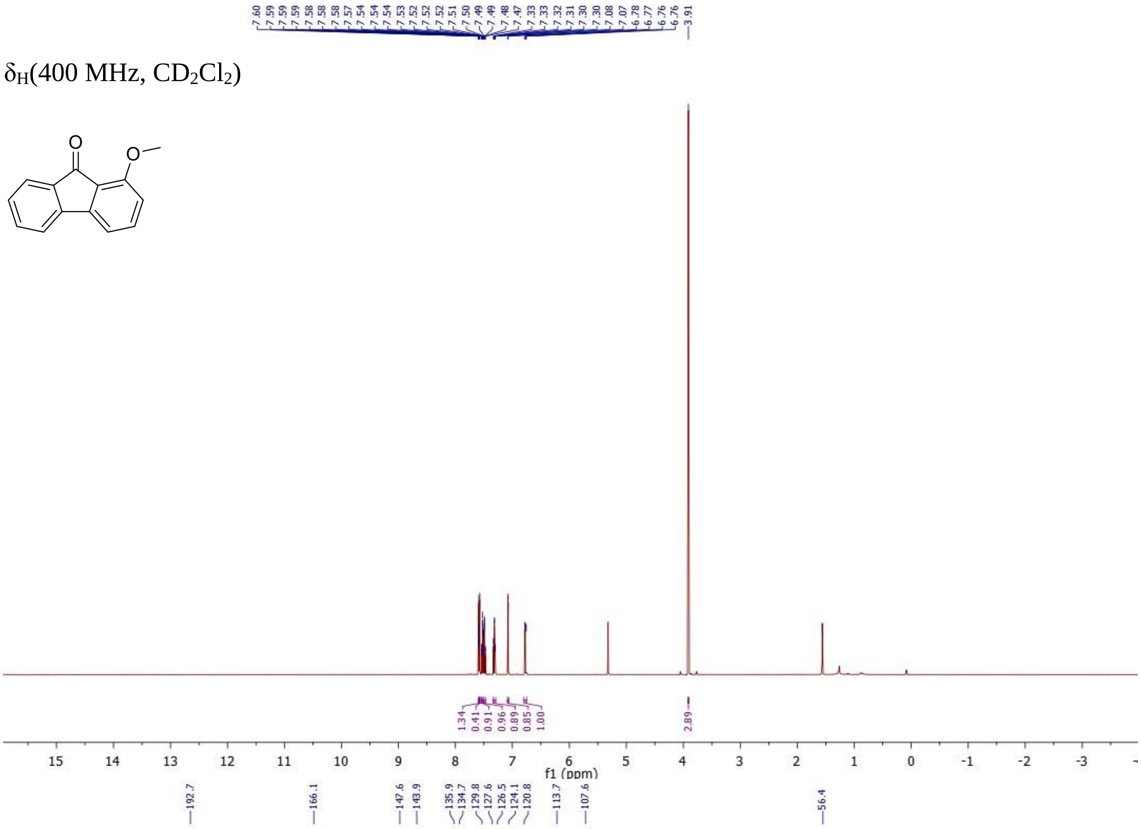

δ_C(101 MHz, CD_2Cl_2)

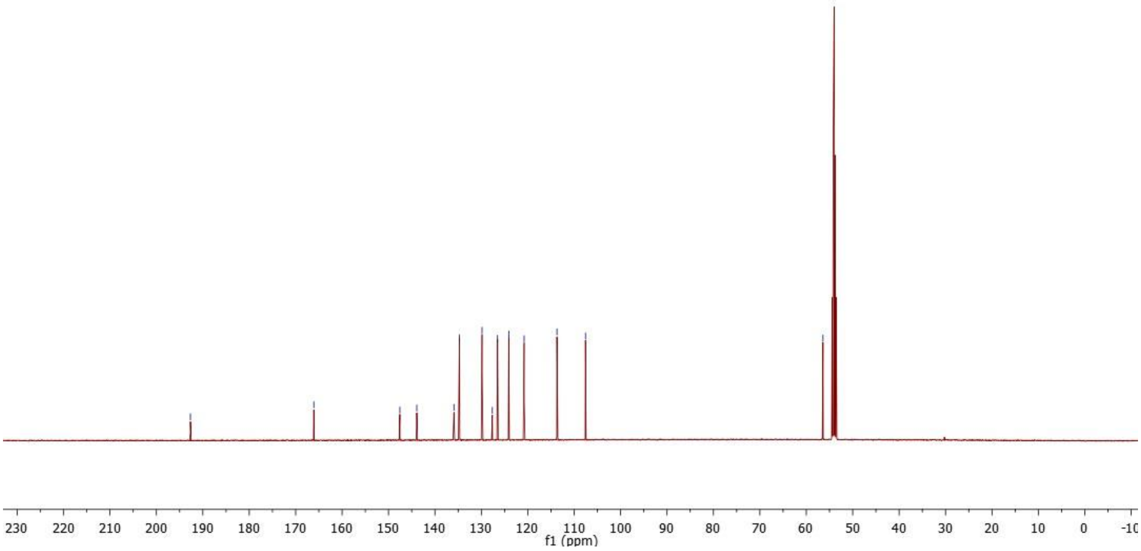

4-(Trifluoromethyl)-9*H*-fluoren-9-one (**10g**)

δ$_H$(500 MHz, CD_2Cl_2)

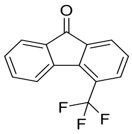

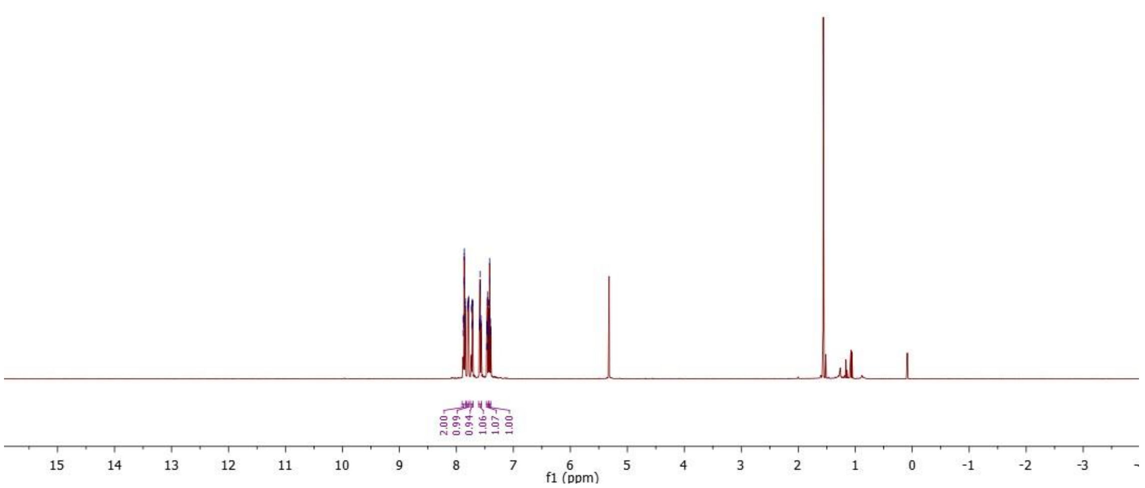

δ$_C$(126 MHz, CD_2Cl_2)

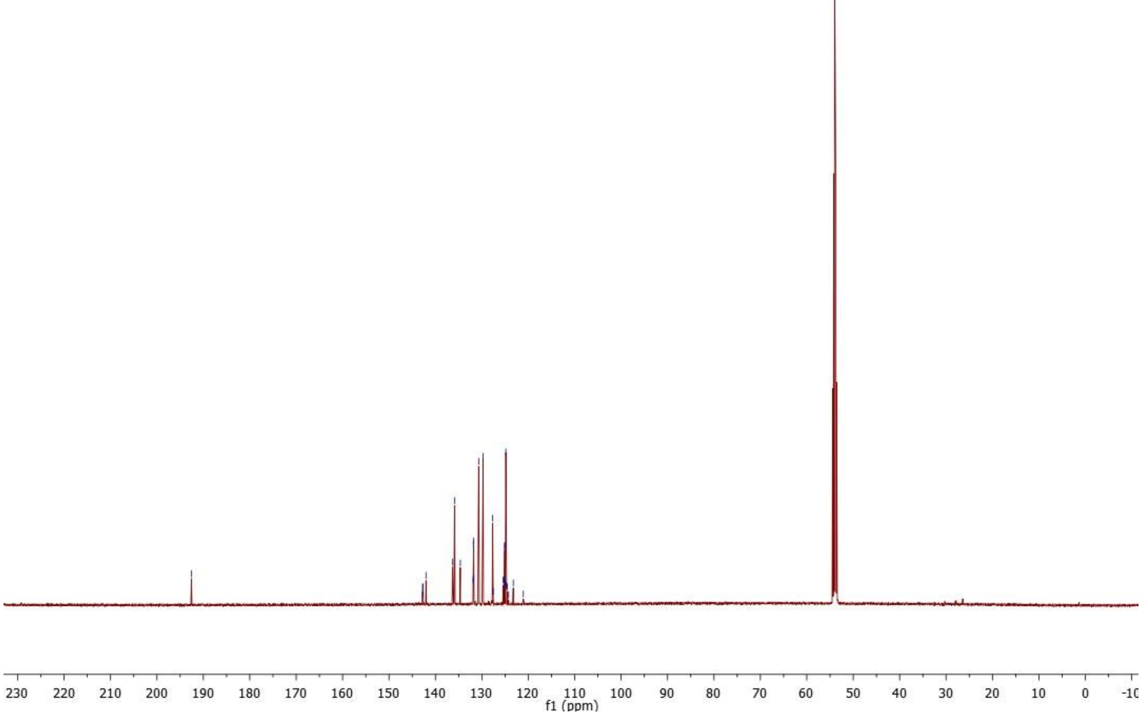

2-(Trifluoromethyl)-9*H*-fluoren-9-one (**10h**)

δ_H(500 MHz, CD_2Cl_2)

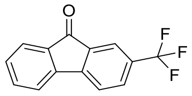

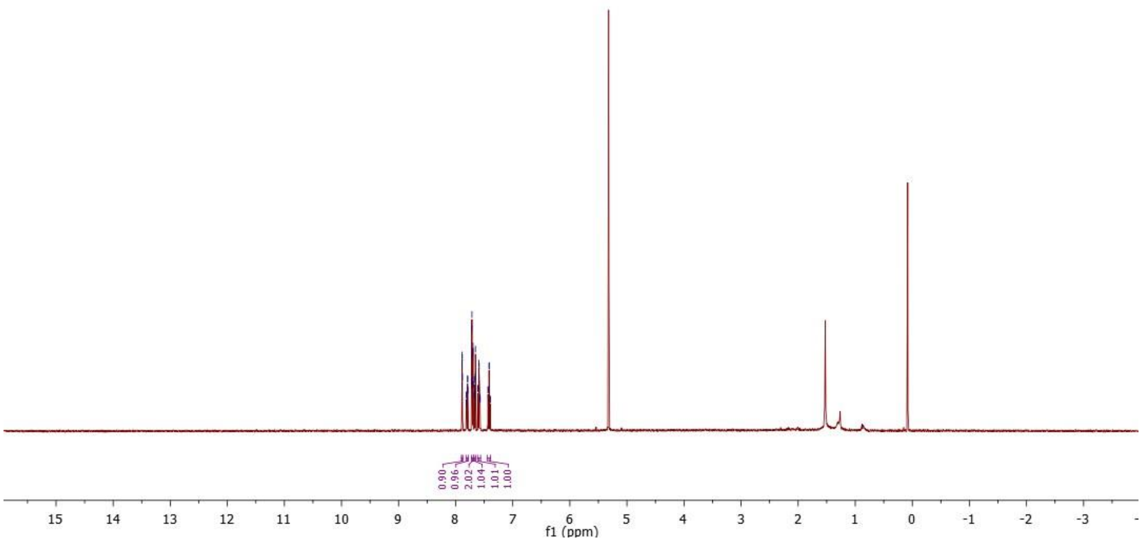

δ_C(126 MHz, CD_2Cl_2)

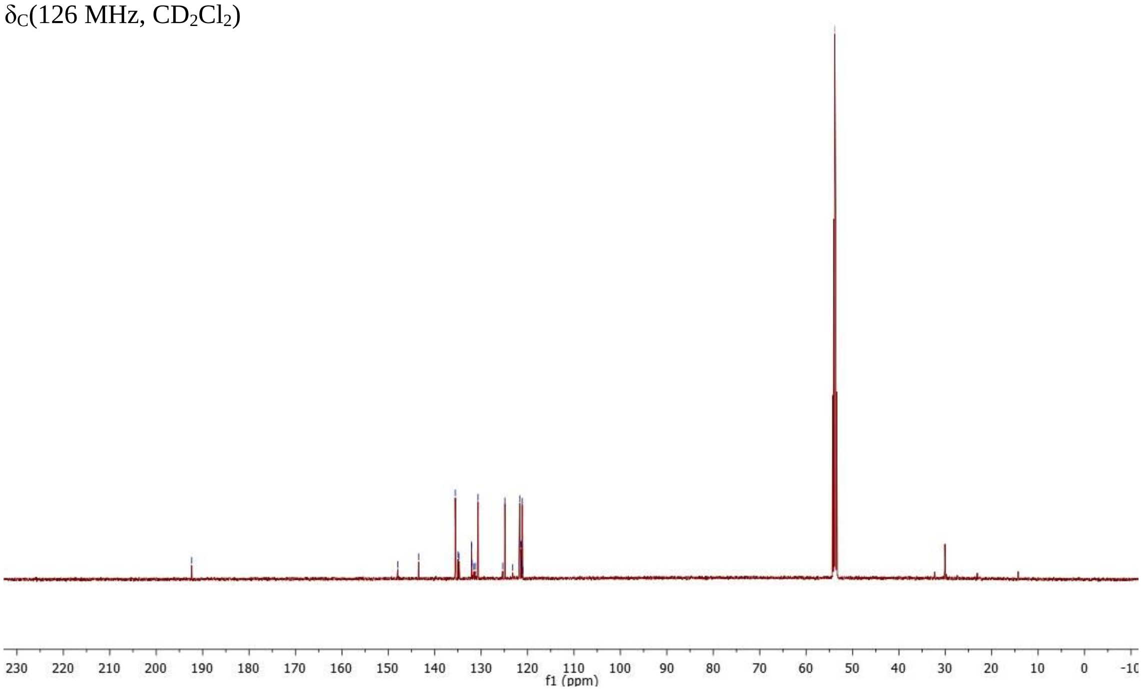

1-(Trifluoromethyl)-9*H*-fluoren-9-one (**10i1**)

δ$_H$(500 MHz, CD_2Cl_2)

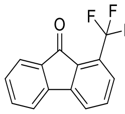

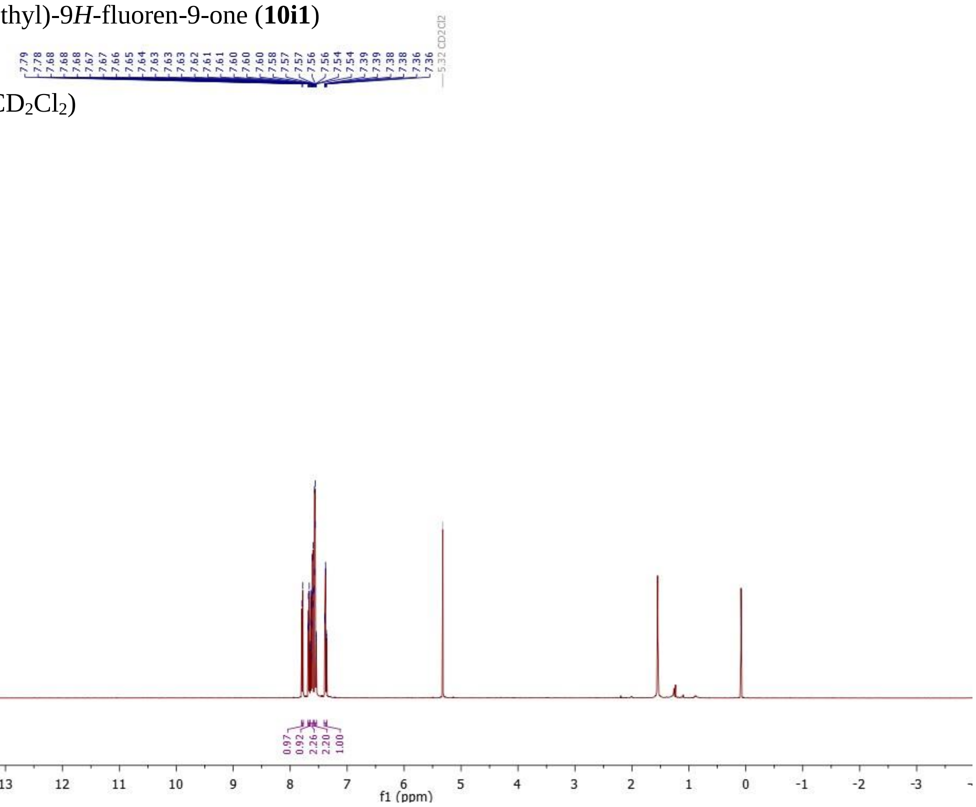

δ$_C$(126 MHz, CD_2Cl_2)

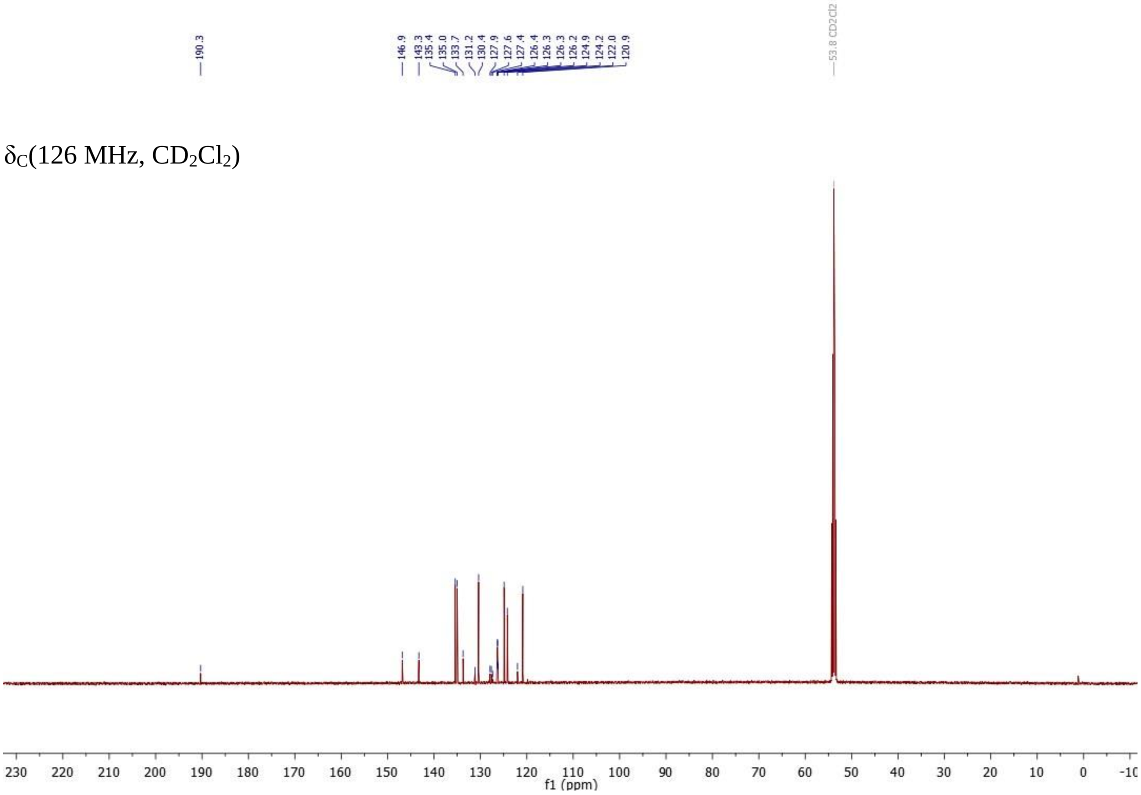

3-(Trifluoromethyl)-9*H*-fluoren-9-one (**10i2**)

δ_H(500 MHz, CD_2Cl_2)

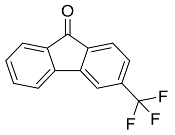

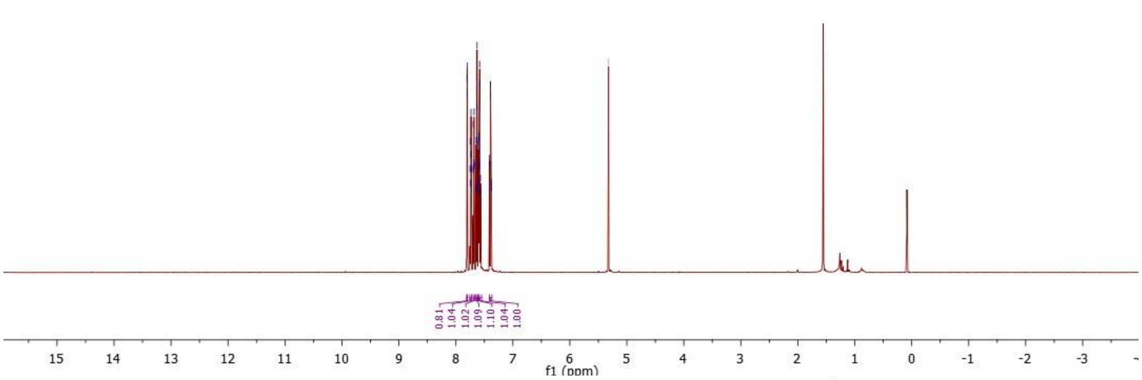

δ_C(126 MHz, CD_2Cl_2)

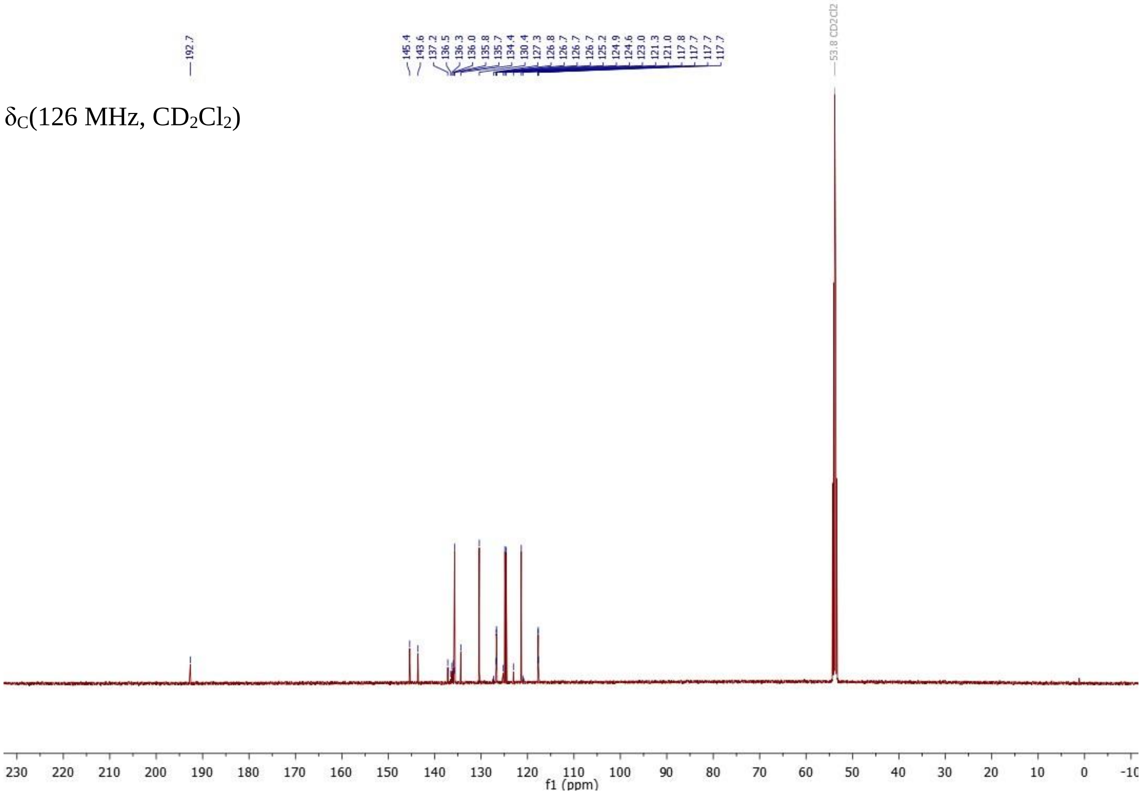

2-Chloro-9*H*-fluoren-9-one (**10j**)

δ_H(400 MHz, CD_2Cl_2)

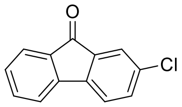

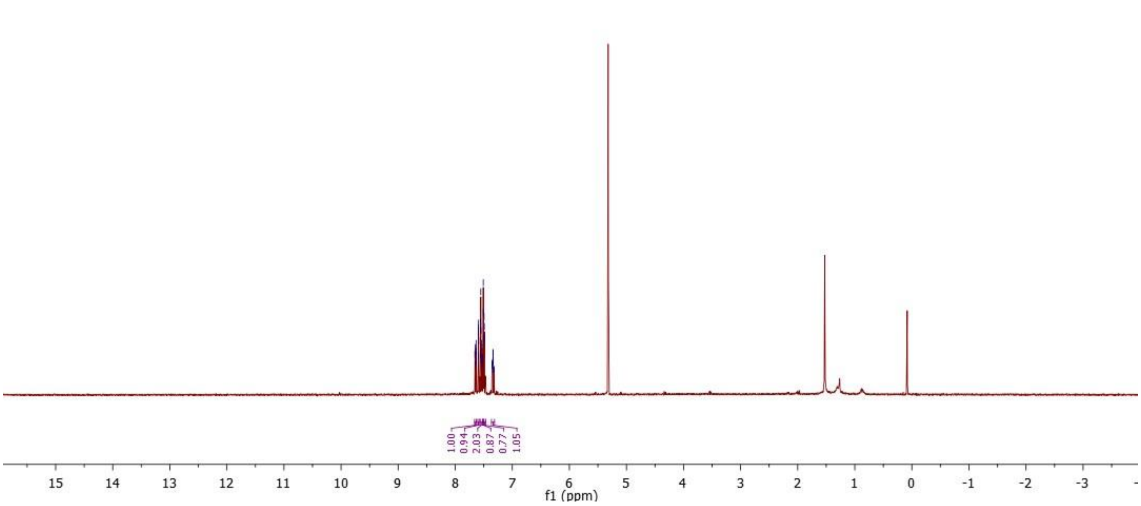

δ_C(101 MHz, CD_2Cl_2)

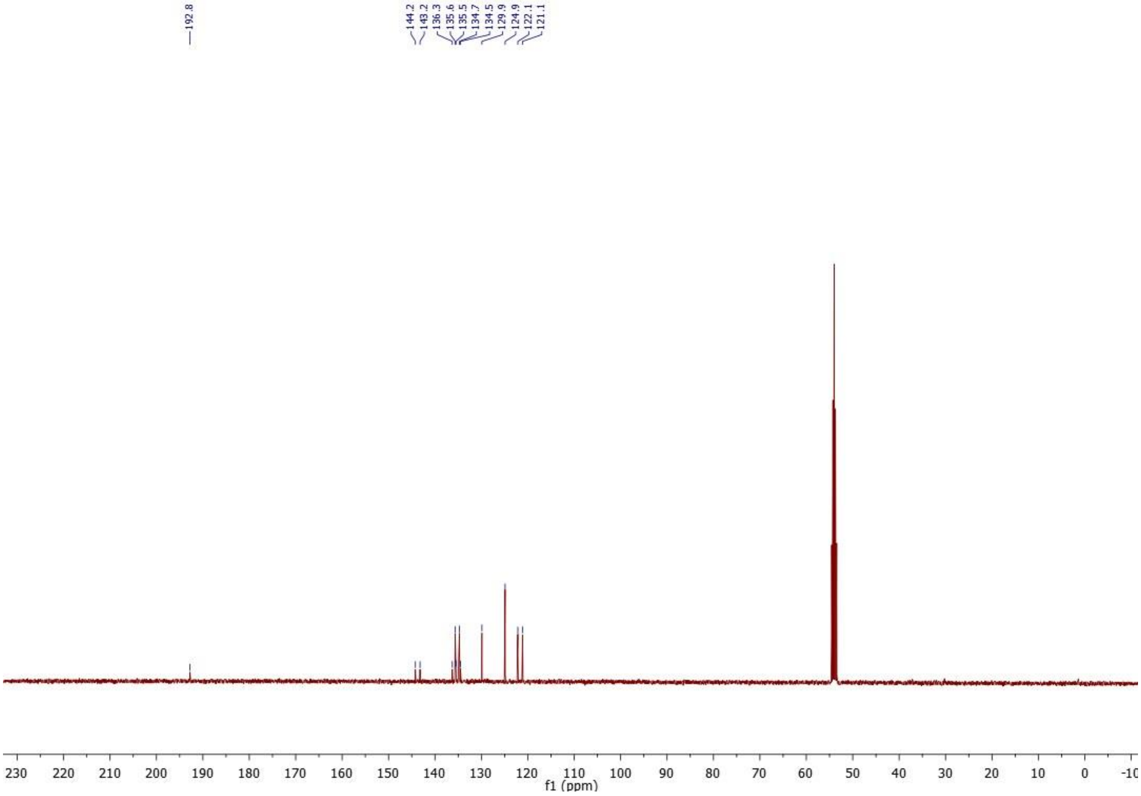

9-Oxo-9*H*-fluorene-2-carbonitrile (**10k**)

δ_H(400 MHz, CD_2Cl_2)

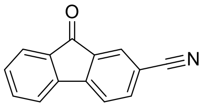

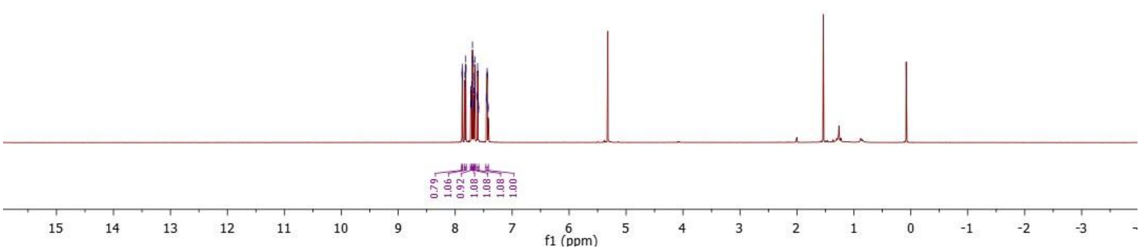

δ_C(101 MHz, CD_2Cl_2)

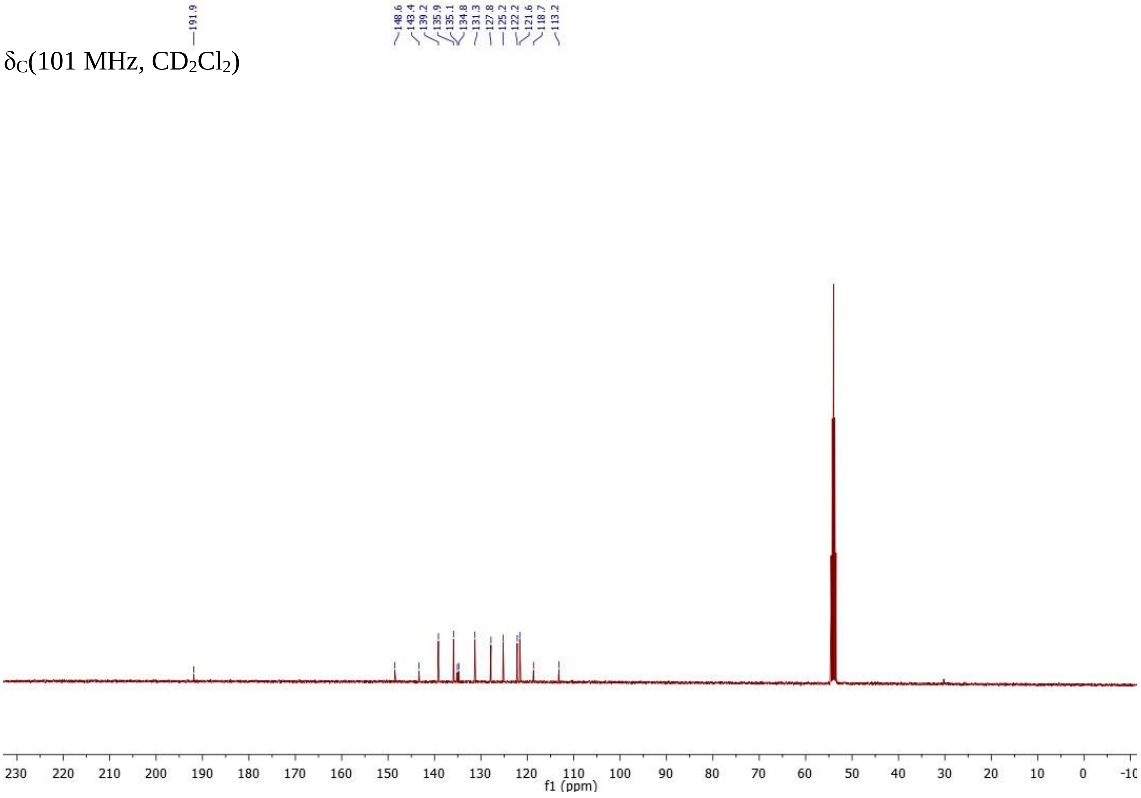

1,3,7-Trimethoxy-9*H*-fluoren-9-one (**10l**)

δ_H(400 MHz, CD_2Cl_2)

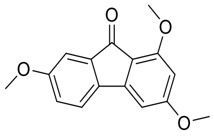

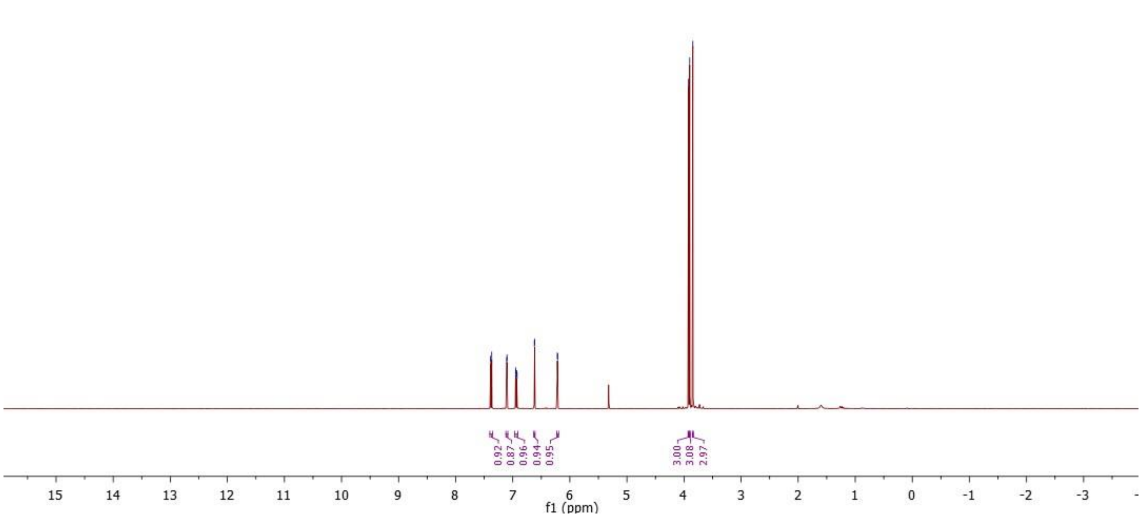

δ_C(101 MHz, CD_2Cl_2)

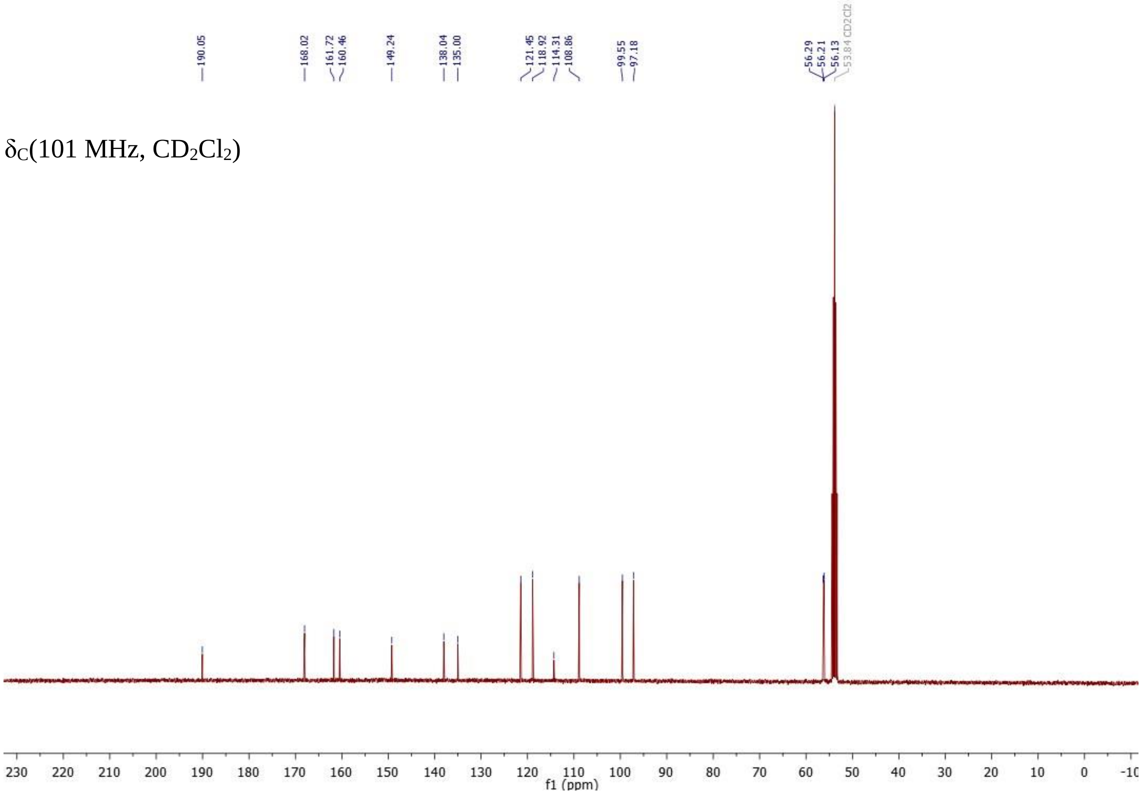

Phenanthridine (**10m**)

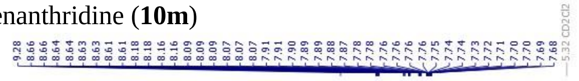

δ$_H$(400 MHz, CD_2Cl_2)

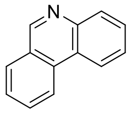

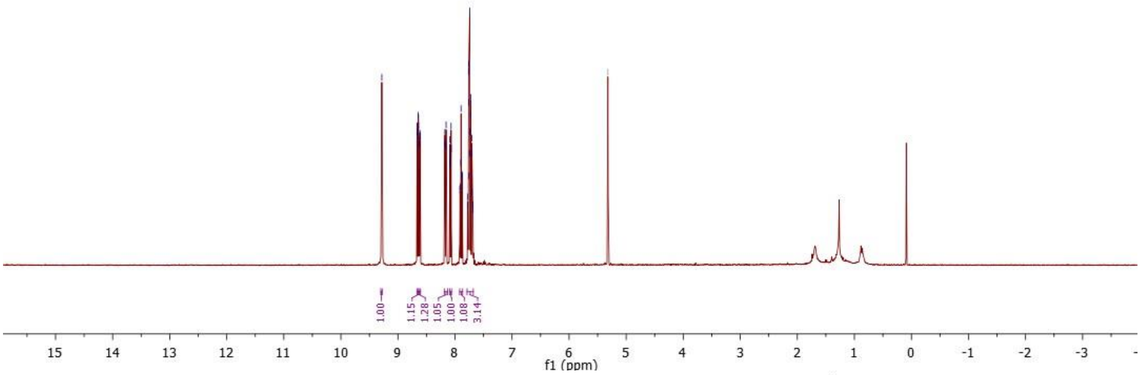

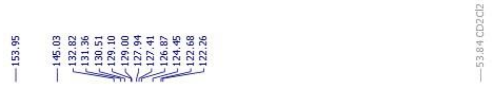

δ$_C$(101 MHz, CD_2Cl_2)

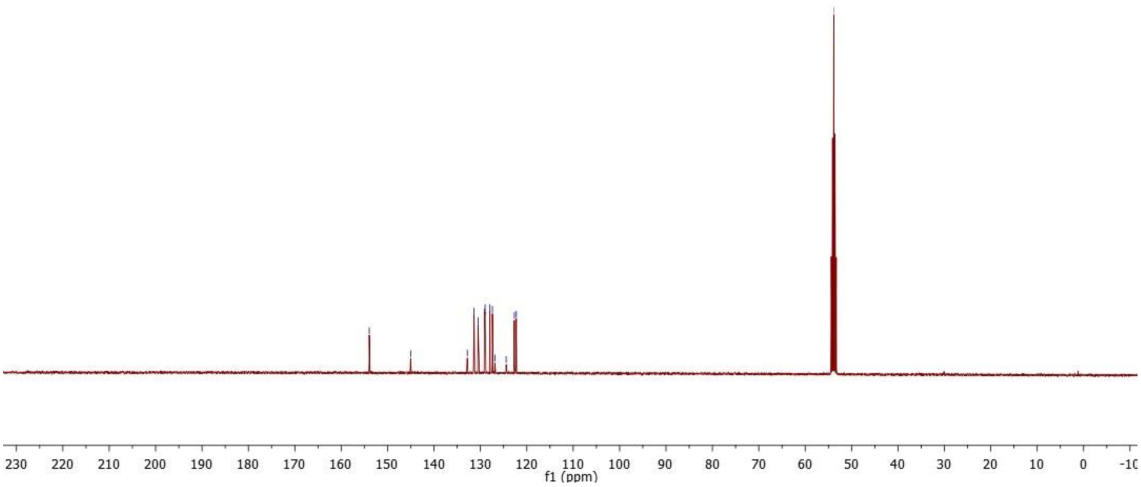

2,3-Dimethoxy-9*H*-fluoren-9-one (**10n1**)

δ$_H$(500 MHz, CD_2Cl_2)

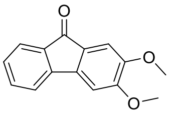

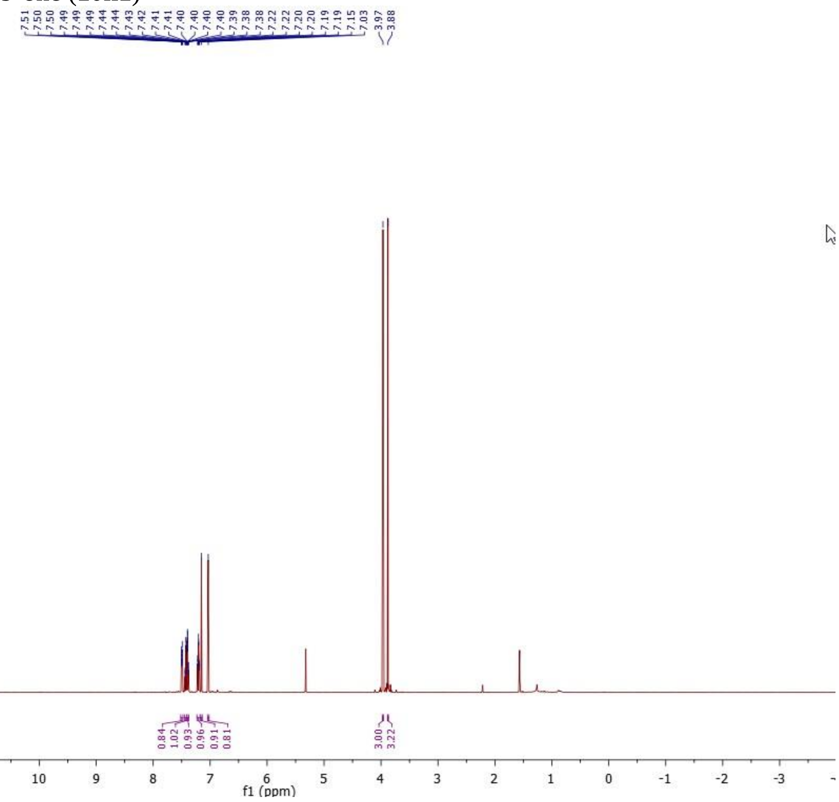

δ$_C$(126 MHz, CD_2Cl_2)

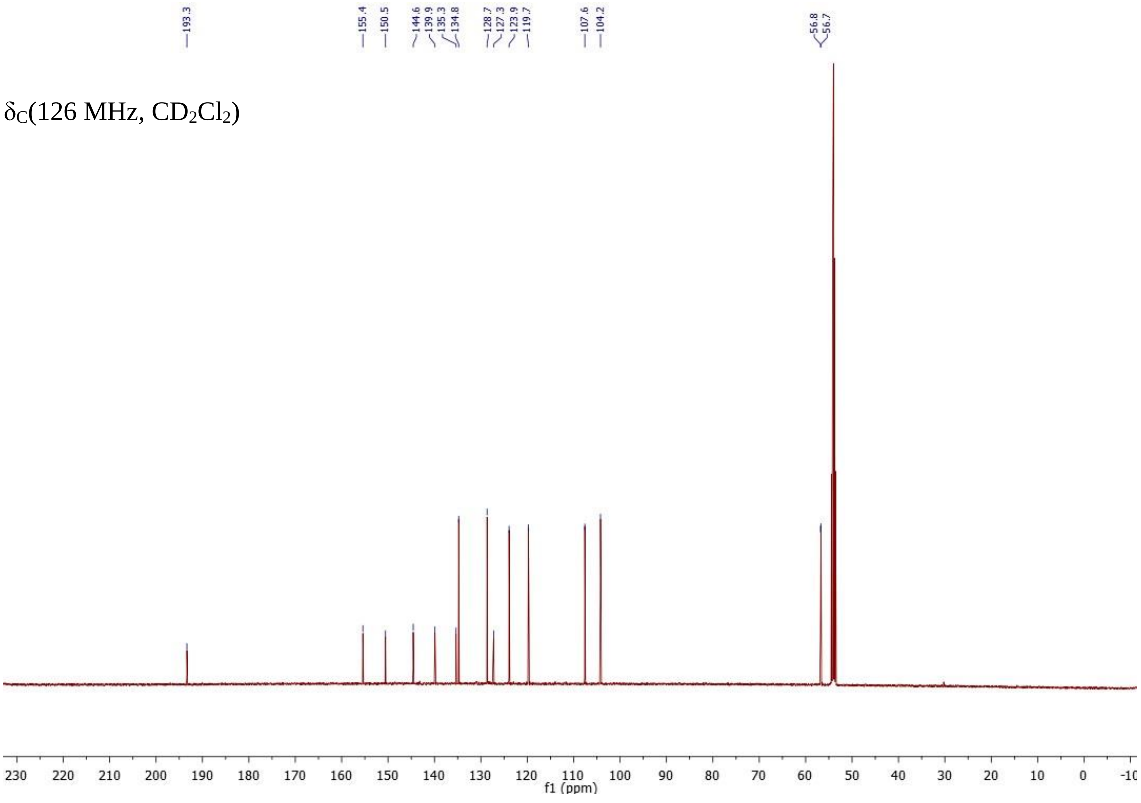

2-Methyl-1-nitro-9*H*-fluoren-9-one (**10o1**)

δ_H(400 MHz, CD_2Cl_2)

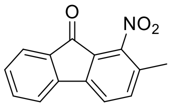

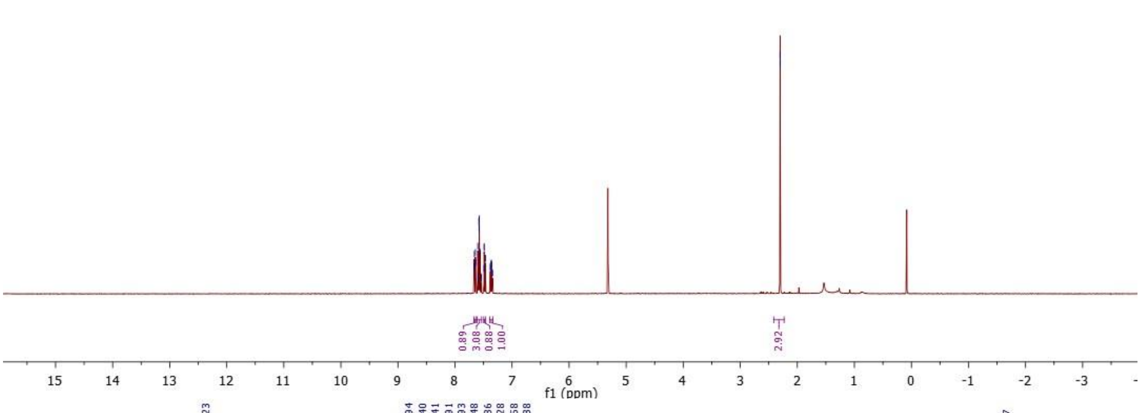

δ_C(101 MHz, CD_2Cl_2)

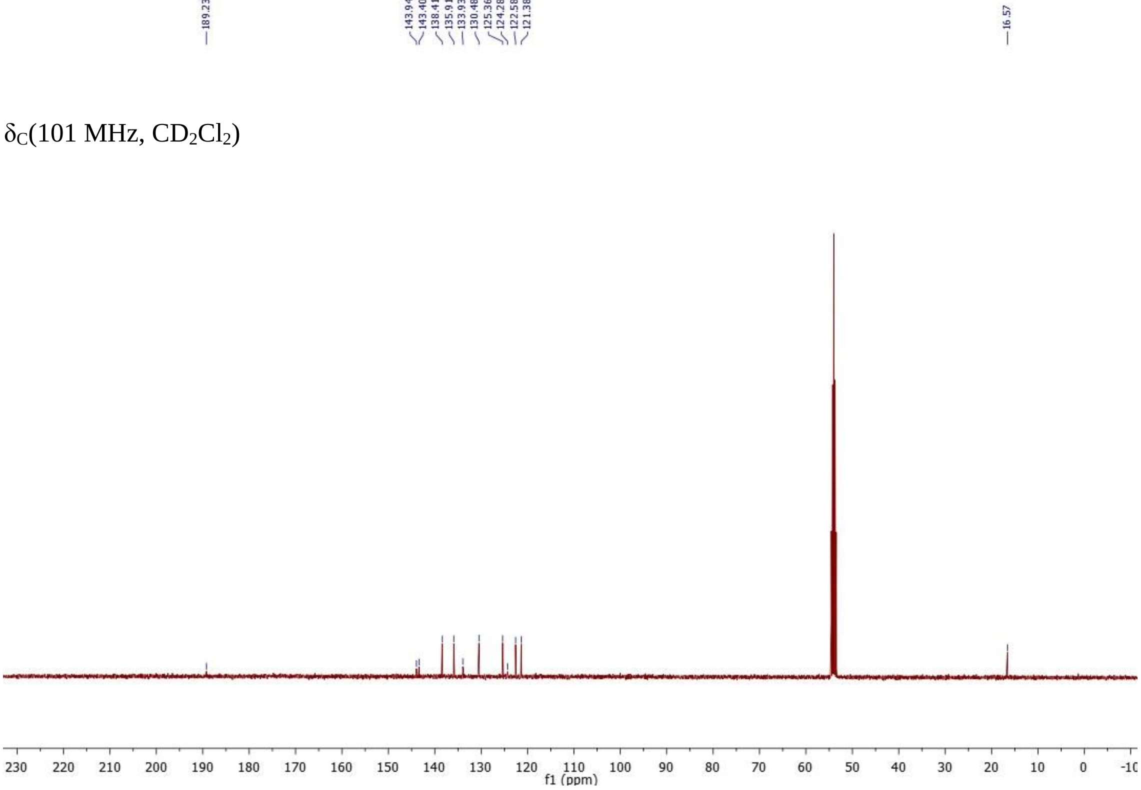

2-Methyl-3-nitro-9*H*-fluoren-9-one (**10o2**)

δ$_H$(400 MHz, CD_2Cl_2)

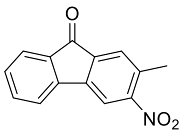

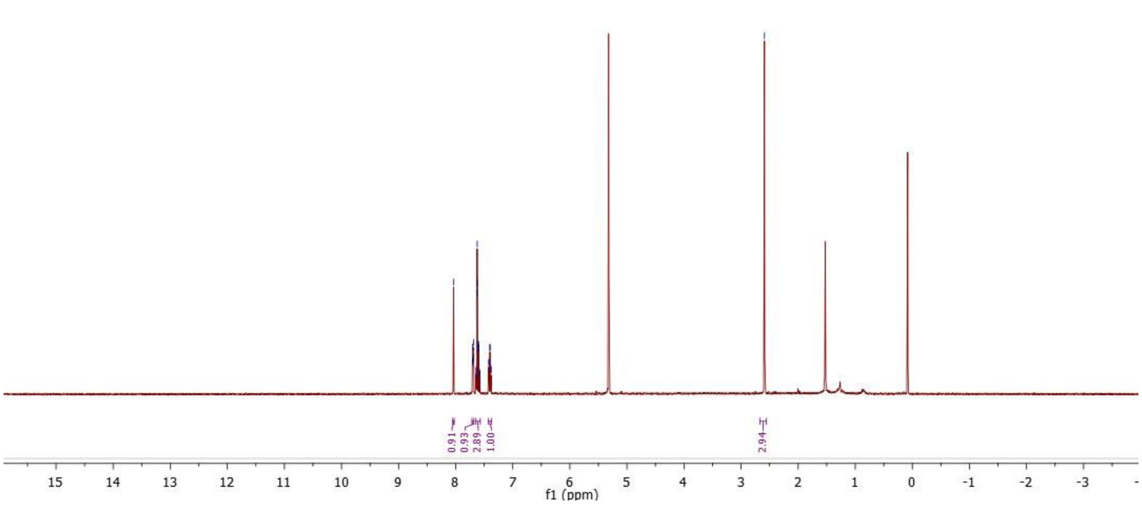

δ$_C$(101 MHz, CD_2Cl_2)

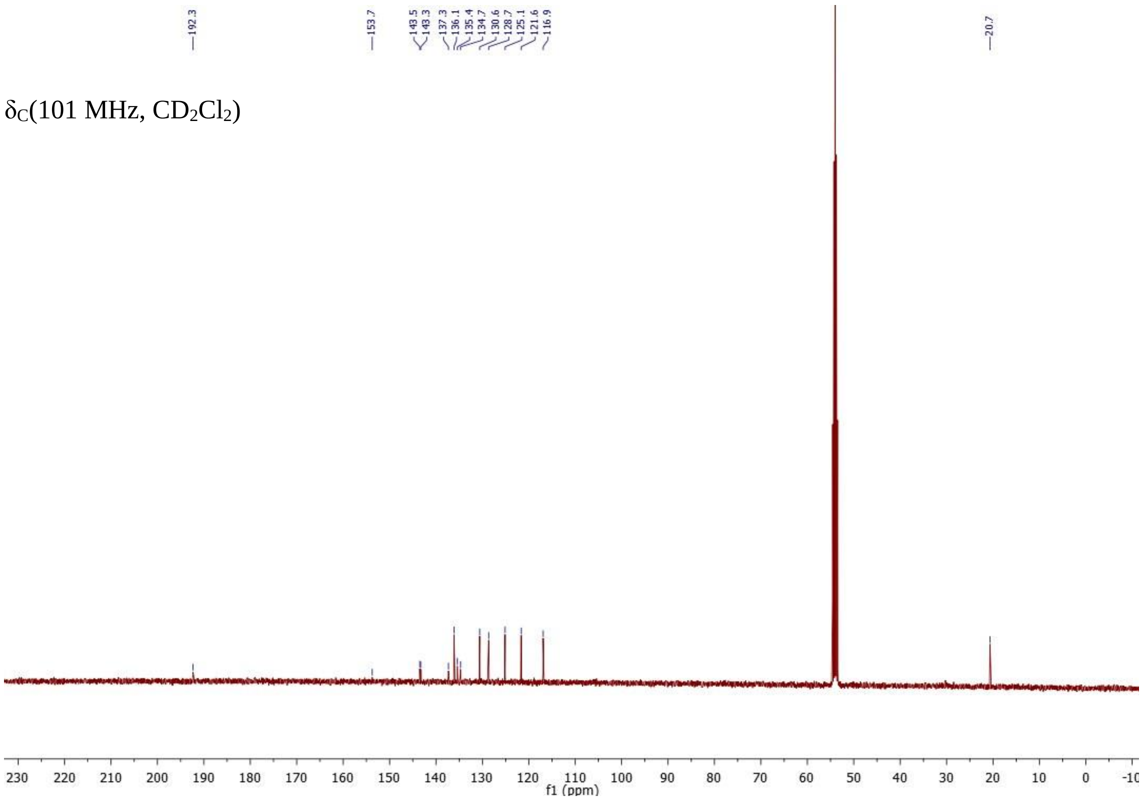

9*H*-fluoreno[2,3-d][1,3]dioxol-9-one (**10p1**)

δ_H(400 MHz, CD_2Cl_2)

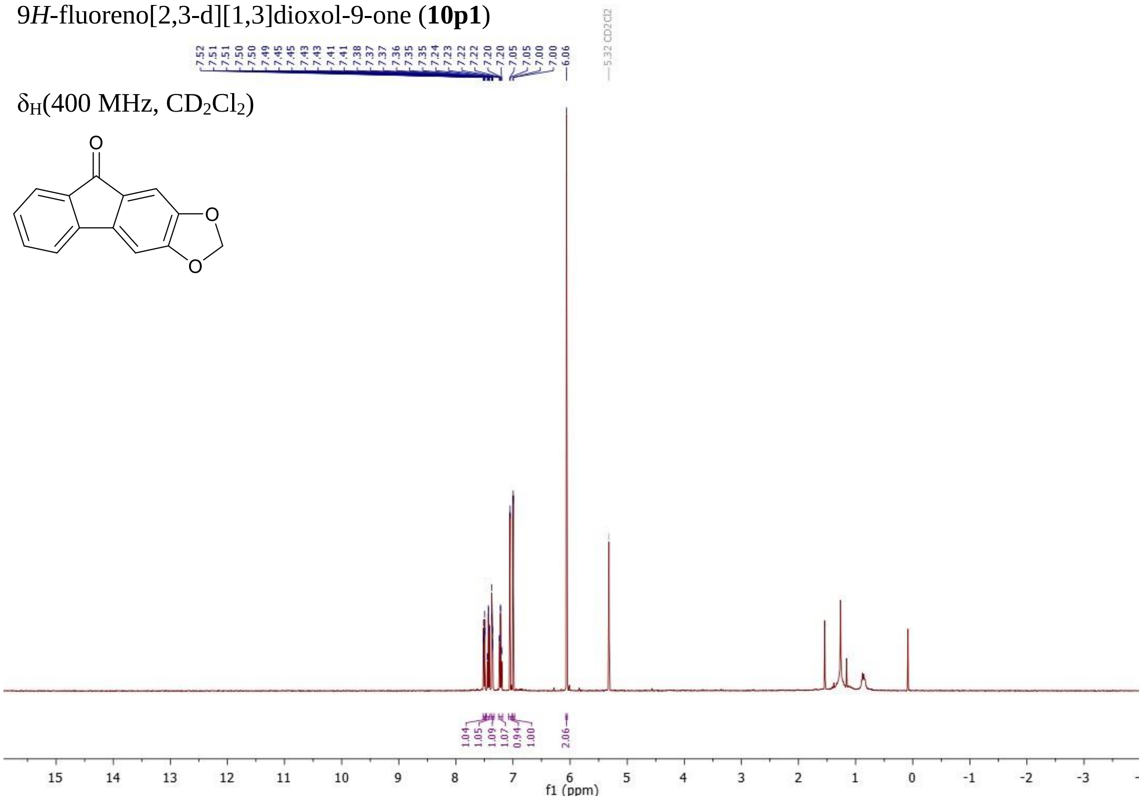

δ_C(101 MHz, CD_2Cl_2)

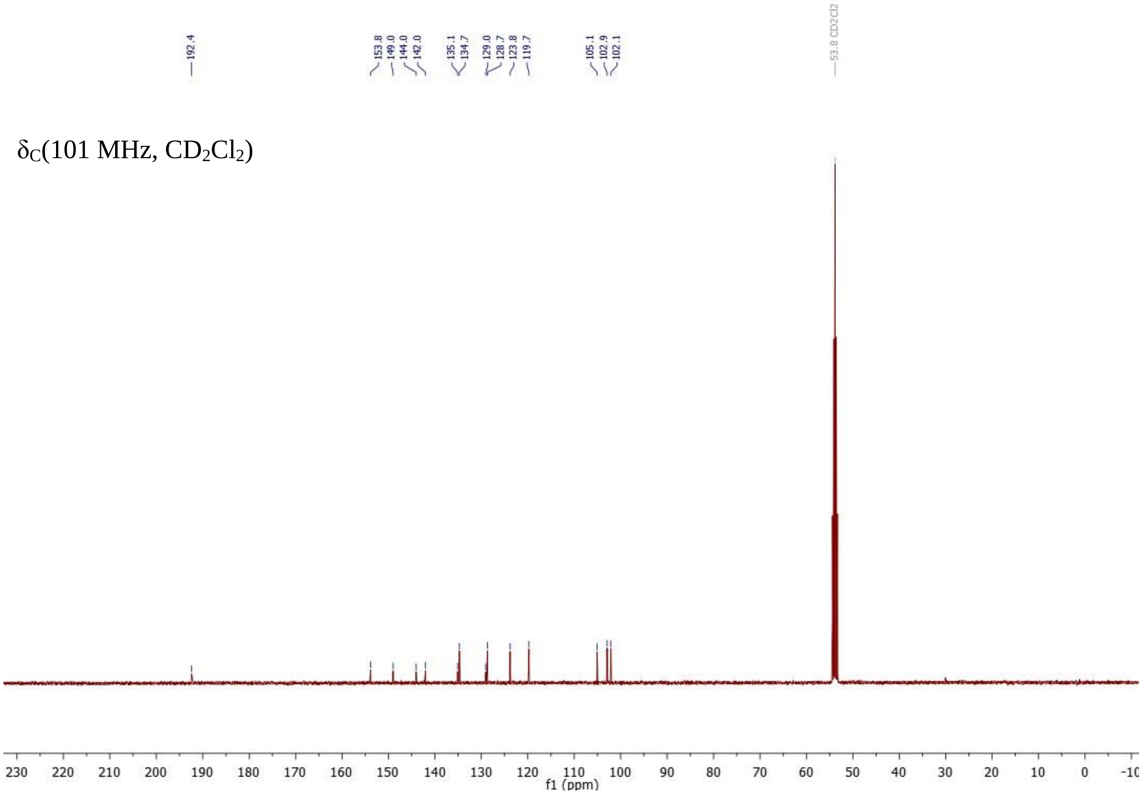

2-Methoxy-1-methyl-9*H*-fluoren-9-one (**10q1**)

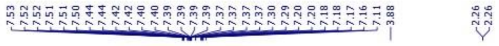

δ_H(400 MHz, CD_2Cl_2)

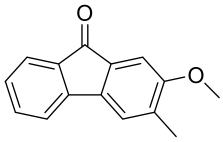

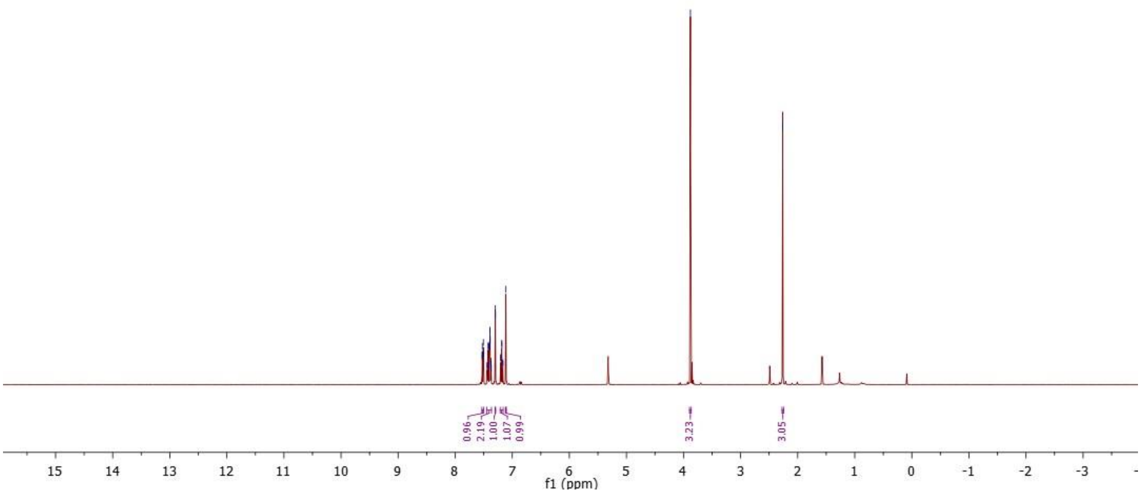

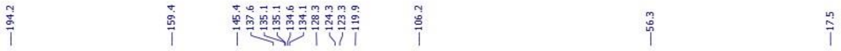

δ_C(101 MHz, CD_2Cl_2)

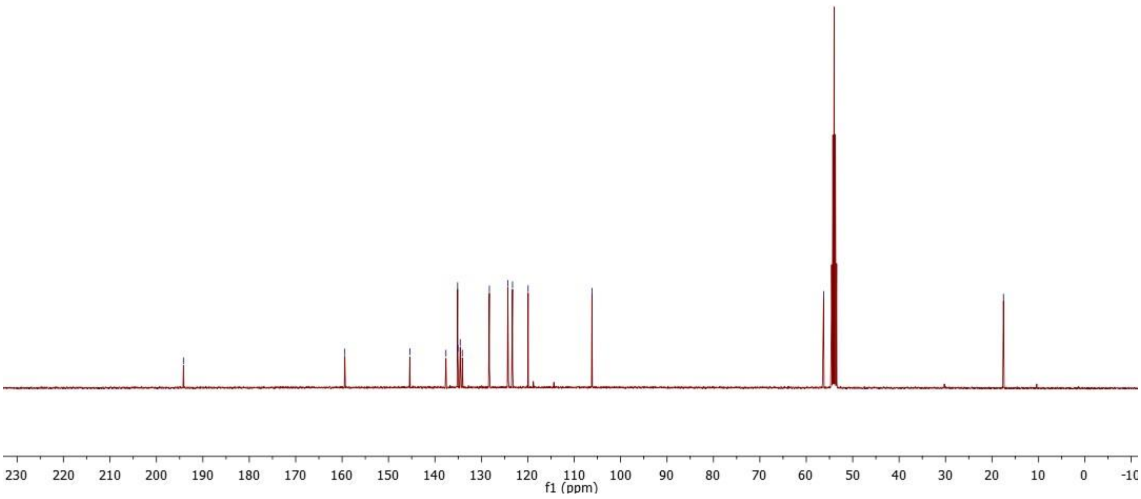

2-Methoxy-3-methyl-9*H*-fluoren-9-one (**10q2**)

δ_H(400 MHz, CD_2Cl_2)

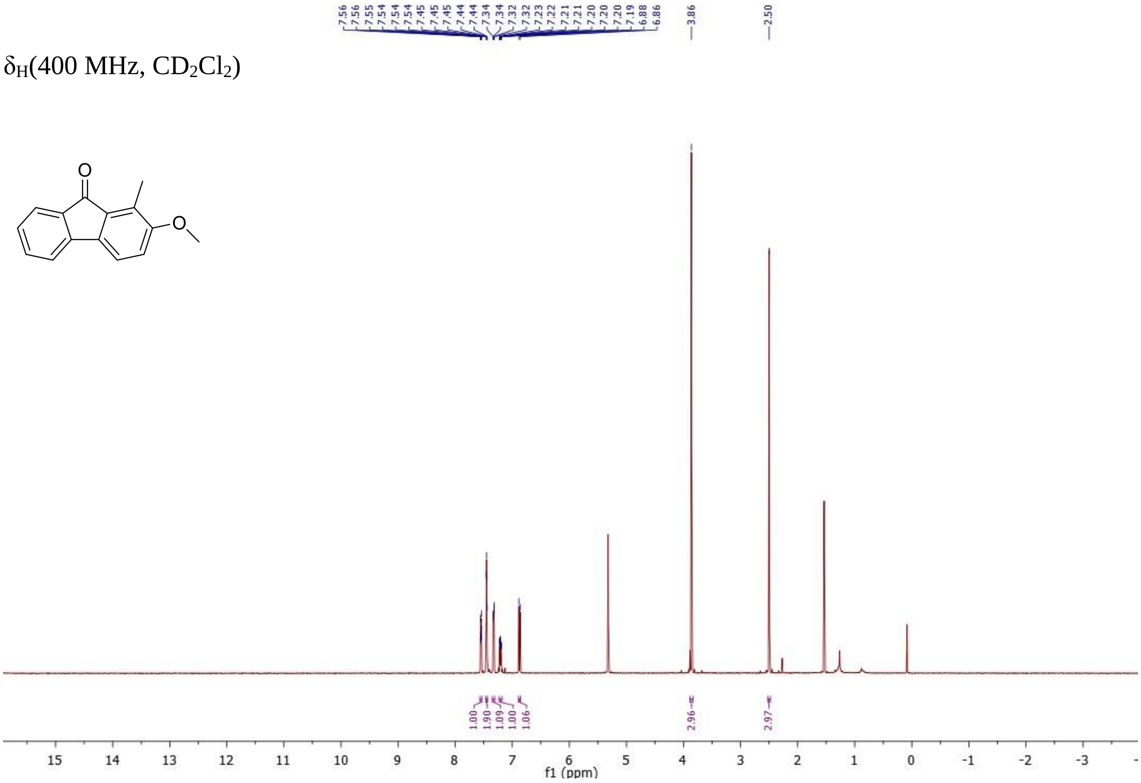

δ_C(101 MHz, CD_2Cl_2)

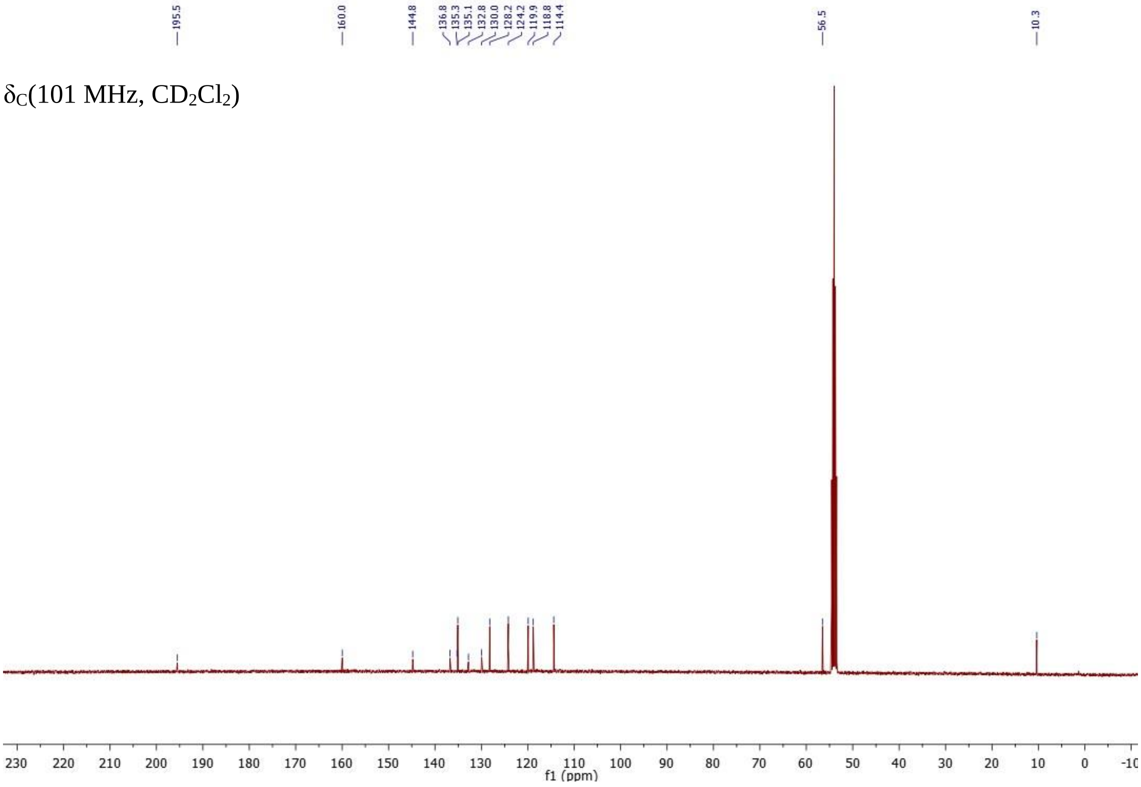

2’-Phenyl-9*H*-fluoren-9-one (**10r**)

7.88 7.88 7.77 7.77 7.75 7.75 7.66 7.66 7.64 7.64 7.64 7.62 7.60 7.60 7.60 7.59 7.58 7.58 7.55 7.55 7.53 7.53 7.51 7.51 7.50 7.49 7.49 7.48 7.47 7.46 7.46 7.41 7.41 7.41 7.40 7.39 7.39 7.38 7.37 7.34 7.34 7.33 7.32 7.31 7.31 5.32 CD2Cl2

δ_H(400 MHz, CD_2Cl_2)

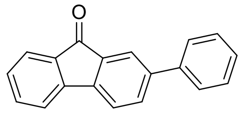

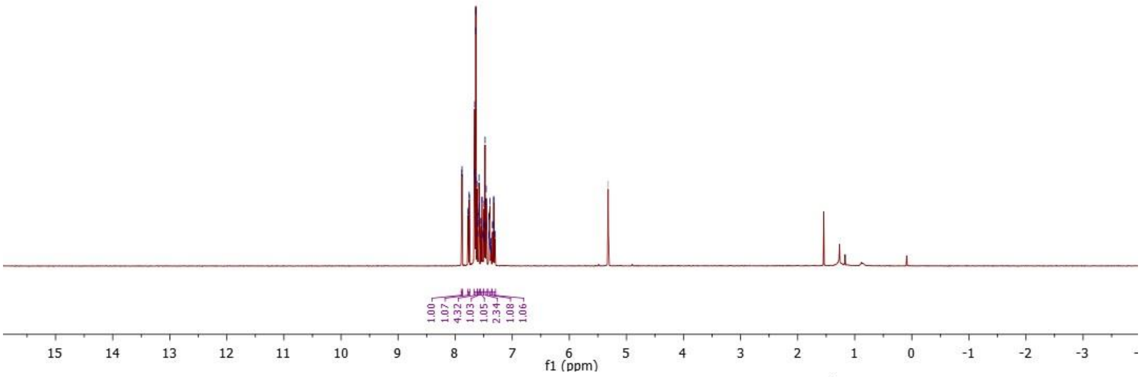

193.86 144.65 143.63 142.60 140.22 135.30 135.24 134.87 133.59 129.44 129.33 128.31 127.15 124.49 123.00 121.20 120.91 53.84 CD2Cl2

δ_C(101 MHz, CD_2Cl_2)

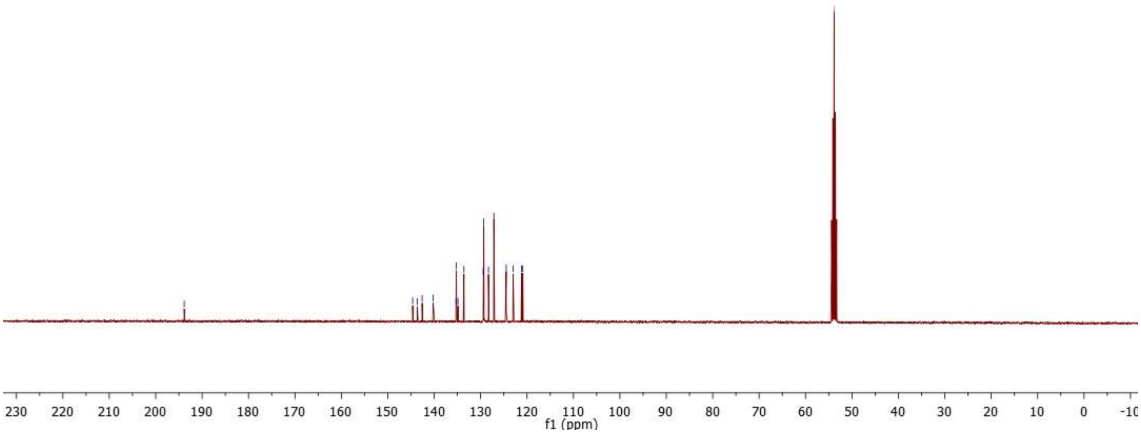

2-Methoxy-1,3-dimethyl-9*H*-fluoren-9-one (**10s**)

δ$_H$(400 MHz, CD_2Cl_2)

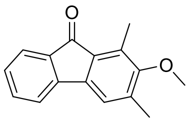

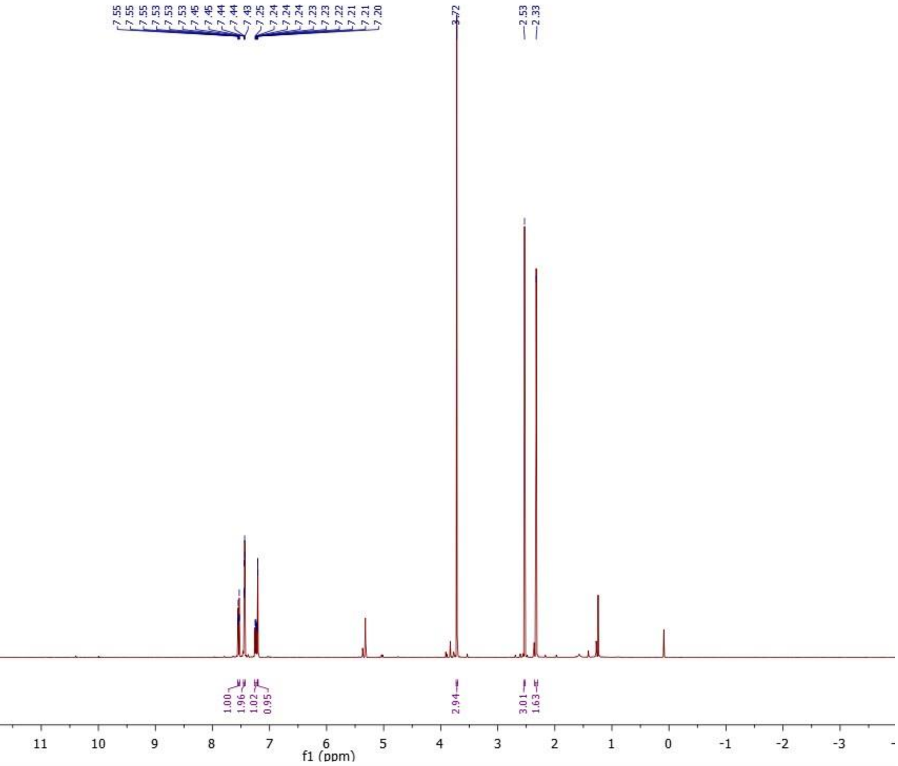

δ$_C$(101 MHz, CD_2Cl_2)

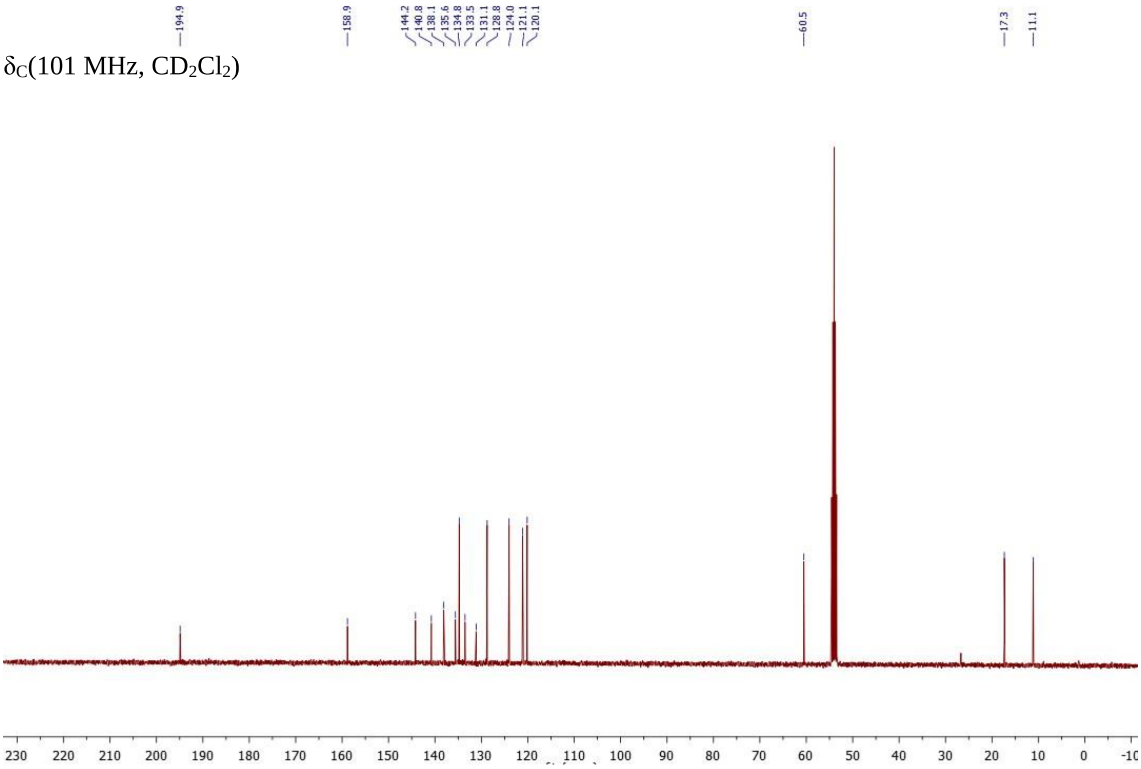

2-((Tert-butyldimethylsilyl)oxy)-9*H*-fluoren-9-one (**10u**)

δ_H(400 MHz, CD_2Cl_2)

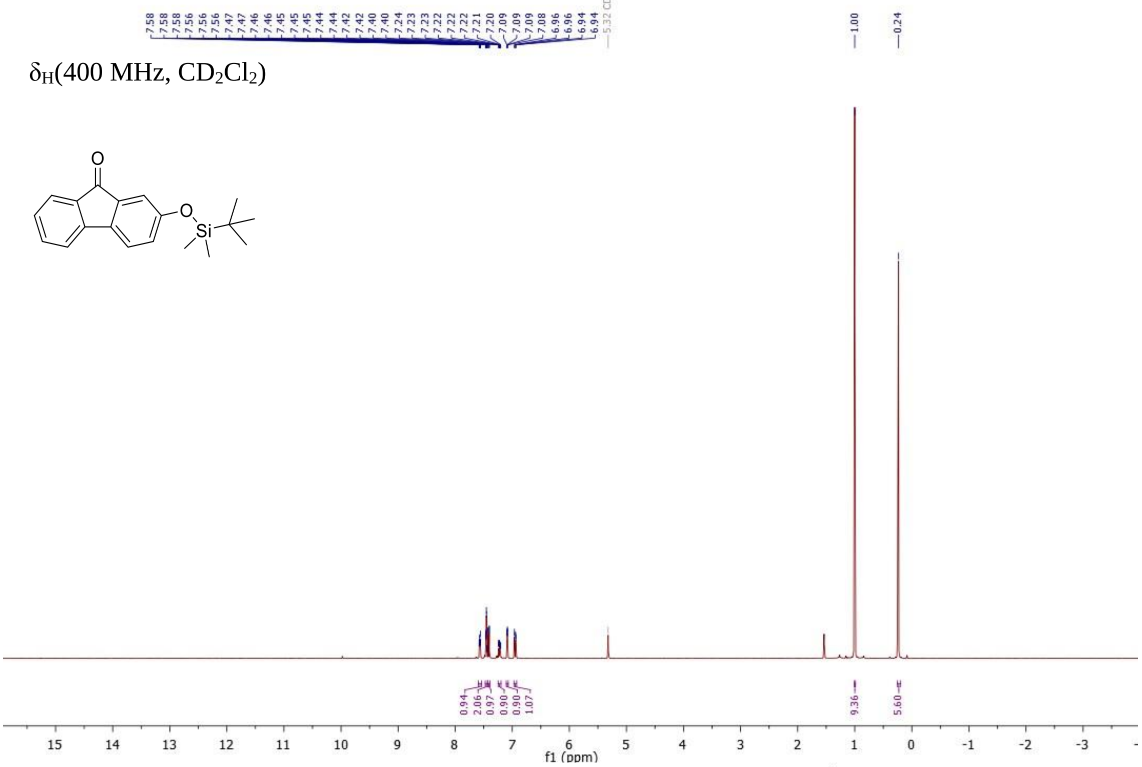

δ_C(101 MHz, CD_2Cl_2)

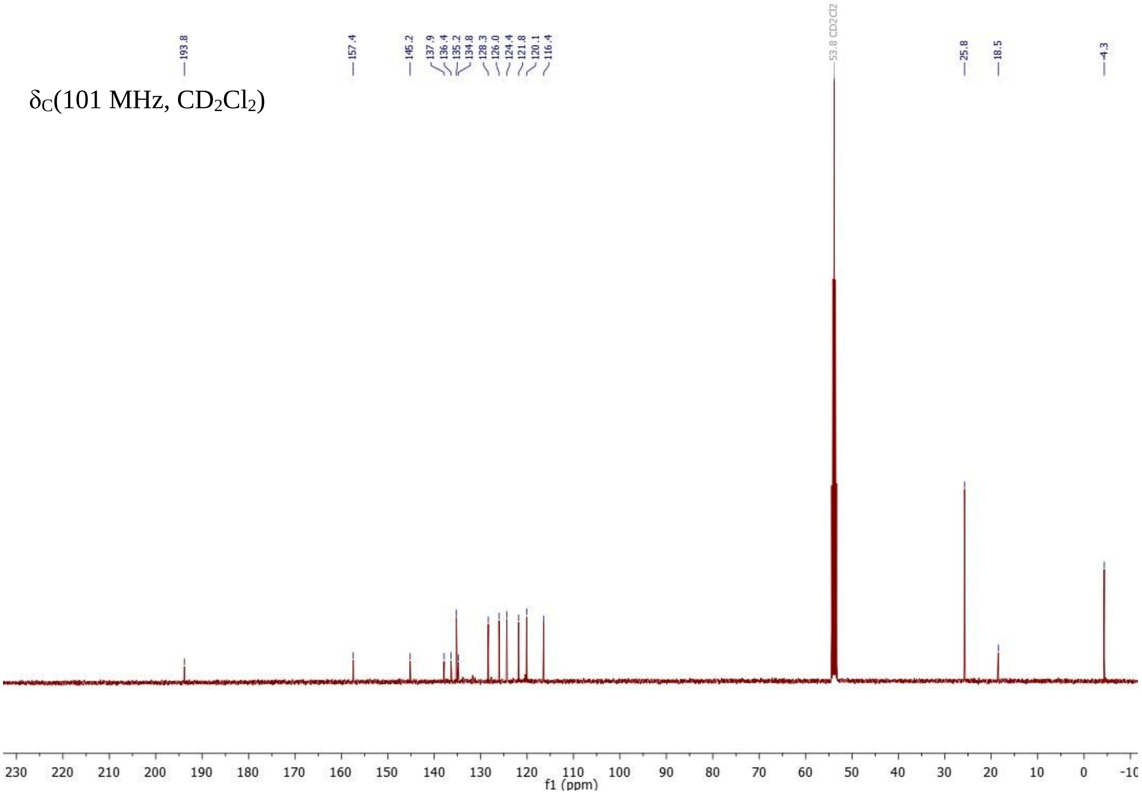

2-((2-(Trimethylsilyl)ethoxy)methoxy)-9*H*-fluoren-9-one (**10v**)

δH(400 MHz, $CDCl_3$)

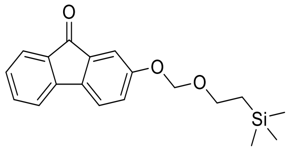

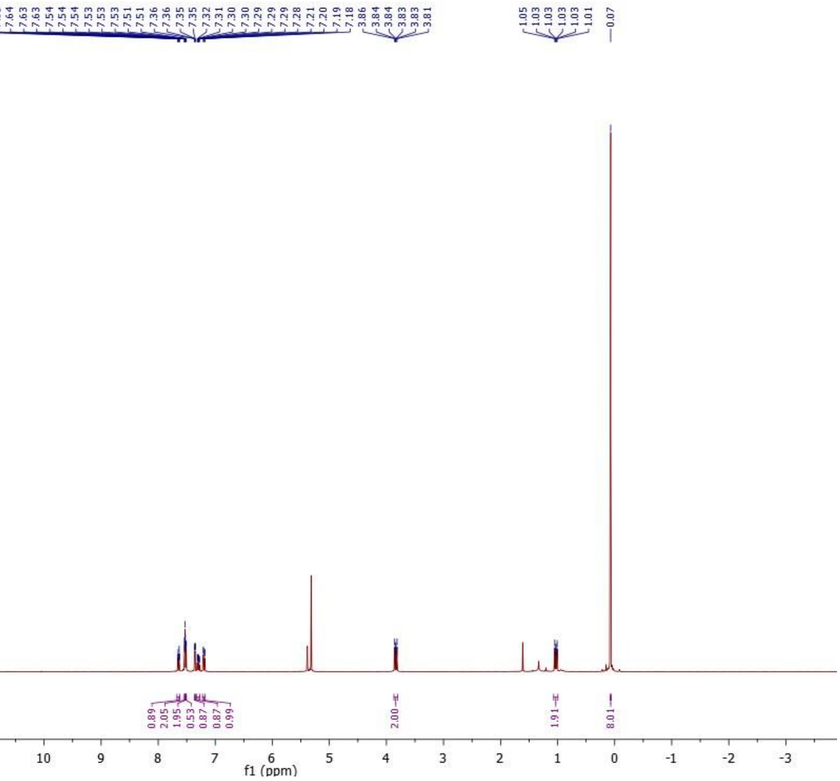

δC(101 MHz, $CDCl_3$)

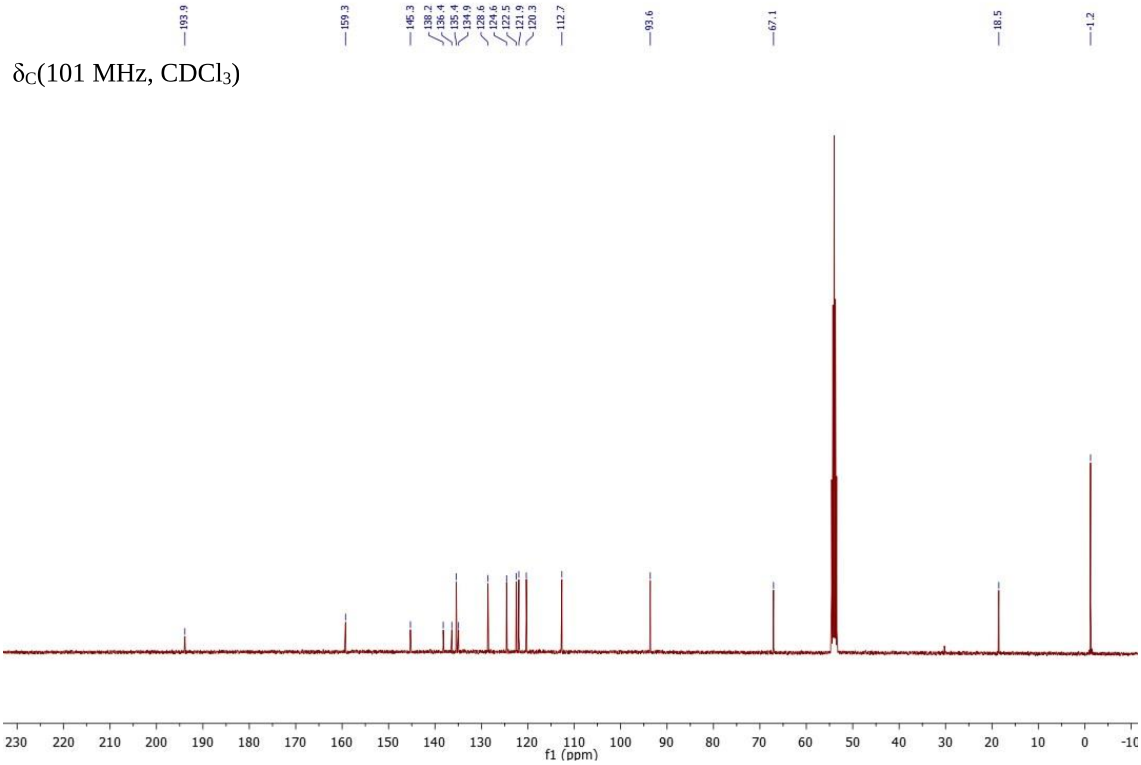

3. References

1. Zhang, X.; Xu, J.-K.; Wang, J.; Wang, N.-L.; Kurihara, H.; Kitanaka, S.; Yao, X.-S. *Journal of Natural Products* **2007**, *70*, 24-28.
2. Ravi Kumar, D.; Satyanarayana, G. *Organic Letters* **2015**, *17*, 5894-5897.
3. Boudreault, P.-L.; Cardinal, S.; Voyer, N. *Synlett* **2010**, *2010*, 2449-2452.
4. Luo, Y.; Wen, Q.; Wu, Z.; Jin, J.; Lu, P.; Wang, Y. *Tetrahedron* **2013**, *69*, 8400-8404.
5. Seo, S.; Slater, M.; Greaney, M. F. *Organic Letters* **2012**, *14*, 2650-2653.
6. Soni, R.; Jolley, K. E.; Gosiewska, S.; Clarkson, G. J.; Fang, Z.; Hall, T. H.; Treloar, B. N.; Knighton, R. C.; Wills, M. *Organometallics* **2018**, *37*, 48-64.
7. Cong, X.; Tang, H.; Zeng, X. *Journal of the American Chemical Society* **2015**, *137*, 14367-14372.
8. Wan, J.-C.; Huang, J.-M.; Jhan, Y.-H.; Hsieh, J.-C. *Organic Letters* **2013**, *15*, 2742-2745.
9. Chen, X.-Y.; Ozturk, S.; Sorensen, E. J. *Organic Letters* **2017**, *19*, 1140-1143.
10. Ruzi, R.; Zhang, M.; Ablajan, K.; Zhu, C. *The Journal of Organic Chemistry* **2017**, *82*, 12834-12839.
11. Bhadra, S.; Dzik, W. I.; Gooßen, L. J. *Angewandte Chemie International Edition* **2013**, *52*, 2959-2962.
12. da Frota, L. C. R. M.; Schneider, C.; de Amorim, M. B.; da Silva, A. J. M.; Snieckus, V. *Synlett* **2017**, *28*, 2587-2593.
13. Truong, T.; Mesgar, M.; Le, K. K. A.; Daugulis, O. *Journal of the American Chemical Society* **2014**, *136*, 8568-8576.
14. Konishi, H.; Futamata, S.; Wang, X.; Manabe, K. *Advanced Synthesis & Catalysis* **2018**, *360*, 1805-1809.
15. Fukuyama, T.; Maetani, S.; Miyagawa, K.; Ryu, I. *Organic Letters* **2014**, *16*, 3216-3219.
16. Moorthy, J. N.; Samanta, S. *The Journal of Organic Chemistry* **2007**, *72*, 9786-9789.
17. Borah, A.; Gogoi, P. *European Journal of Organic Chemistry* **2016**, *2016*, 2200-2206.
18. Zhang, X.; Larock, R. C. *Organic Letters* **2005**, *7*, 3973-3976.
19. Tang, J.; Luo, M.; Zeng, X. *Synlett* **2017**, *28*, 2577-2580.
20. Yamamoto, Y.; Nakanishi, Y.; Yamada, K.-i.; Tomioka, K. *Tetrahedron* **2018**, *74*, 5309-5318.
21. Zhou, C.; Liu, Q.; Li, Y.; Zhang, R.; Fu, X.; Duan, C. *The Journal of Organic Chemistry* **2012**, *77*, 10468-10472.
22. Chen, W.-L.; Chen, C.-Y.; Chen, Y.-F.; Hsieh, J.-C. *Organic Letters* **2015**, *17*, 1613-1616.
23. Li, Y.; Dong, Y.; Wei, Y.-L.; Jv, J.-J.; Chen, Y.-Q.; Ma, J.-P.; Yao, J.-J.; Dong, Y.-B. *Organometallics* **2018**, *37*, 1645-1648.
24. Salamanca, V.; Toledo, A.; Albéniz, A. C. *Journal of the American Chemical Society* **2018**, *140*, 17851-17856.
25. Ahmed, J.; Chakraborty, S.; Jose, A.; P, S.; Mandal, S. K. *Journal of the American Chemical Society* **2018**, *140*, 8330-8339.
26. Sarkar, S.; Jana, M.; Narender, T. *European Journal of Organic Chemistry* **2013**, *2013*, 6491-6495.
27. Kang, K.; Huang, L.; Weix, D. J. *Journal of the American Chemical Society* **2020**, *142*, 10634-10640.
28. Li, X.; Feng, F.; Ren, C.; Teng, Y.; Hu, Q.; Yuan, Z. *Synlett* **2019**, *30*, 2131-2135.
29. Bag, S.; Patra, T.; Modak, A.; Deb, A.; Maity, S.; Dutta, U.; Dey, A.; Kancherla, R.; Maji, A.; Hazra, A.; Bera, M.; Maiti, D. *Journal of the American Chemical Society* **2015**, *137*, 11888-11891.
30. Chen, X.; Martinez, J. S.; Mohr, J. T. *Organic Letters* **2015**, *17*, 378-381.
31. Asai, N.; Iwasaki, F.; Kurosaki, T.; Negoro, K.; Ohnuki, K.; Soga, T.; Yonetoku, Y.; Yoshida, S. Oxadiazolidinedione Compound. Pat. Appl. JP2007058694W, Pat. WO2007123225A1, 2007/11/01/, 2007.
32. Oberhauser, T. *The Journal of Organic Chemistry* **1997**, *62*, 4504-4506.
